# Two decades of progress in C4-unsubstituted3-acetylcoumarins: synthesis, reactivity, heterocyclic diversity, and applications

**DOI:** 10.1039/d6ra05738a

**Published:** 2026-11-06

**Authors:** Moaz M. Abdou, Luai Z. Hasoun, Noura M. Hammad, Mohammad Albayari, Eslam A. Ghaith, Mahmoud M. Fayad, Ahmed I. Khodair

**Affiliations:** a Egyptian Petroleum Research Institute Cairo 11727 Egypt moaz.chem@gmail.com; b Chemistry Department, Faculty of Science, Applied Science Private University Amman 11931 Jordan; c Chemistry Department, Faculty of Science, Kafrelsheikh University Kafrelsheikh 33516 Egypt khodair2020@yahoo.com; d Department of Chemistry, Faculty of Science, Mansoura University Mansoura 31556 Egypt; e Chemistry Department, Faculty of Science, New Mansoura University New Mansoura 35712 Egypt

## Abstract

C4-unsubstituted 3-acetylcoumarins represent a versatile class of coumarin-based heterocycles that combine a reactive acetyl group at the C-3 position with an accessible C4–H site. This structural arrangement makes them valuable synthons for functionalization, annulation and the construction of diverse heterocyclic frameworks. This review summarizes the progress reported from 2006 to 2026 in the synthesis, reactivity and applications of C4-unsubstituted 3-acetylcoumarin derivatives. The main synthetic strategies include Knoevenagel condensation, diketene-based routes, alkynoate-mediated synthesis, hydrodehalogenation, cross-dehydrogenative coupling and carbonyl–alkyne metathesis. Their reactivity covers halogenation, reduction, thionation, phosphorylation, Mannich reactions, condensation with nitrogen nucleophiles, C–H functionalization and cycloaddition reactions. These transformations provide access to a broad range of coumarin-containing heterocycles, including pyrazoles, thiazoles, oxazoles, isoxazoles, pyridines, pyrimidines, thiophenes, triazoles, quinoxalines, diazepines and fused polycyclic systems. Beyond synthetic chemistry, many derivatives show promising biological activities, including antibacterial, antioxidant, anti-inflammatory, anti-Alzheimer, antitumor, antidiabetic and enzyme-inhibitory effects. Their photophysical properties have also enabled their application as fluorescent chemosensors for ions, reactive species and biologically relevant molecules. Finally, this review highlights future opportunities in greener synthesis, structure–activity relationship analysis and advanced biological evaluation.

## Abstract 2

C4-unsubstituted 3-acetylcoumarins represent a versatile class of coumarin-based heterocycles that combine a reactive acetyl group at the C-3 position with an accessible C4–H site.

## Graphical abstract

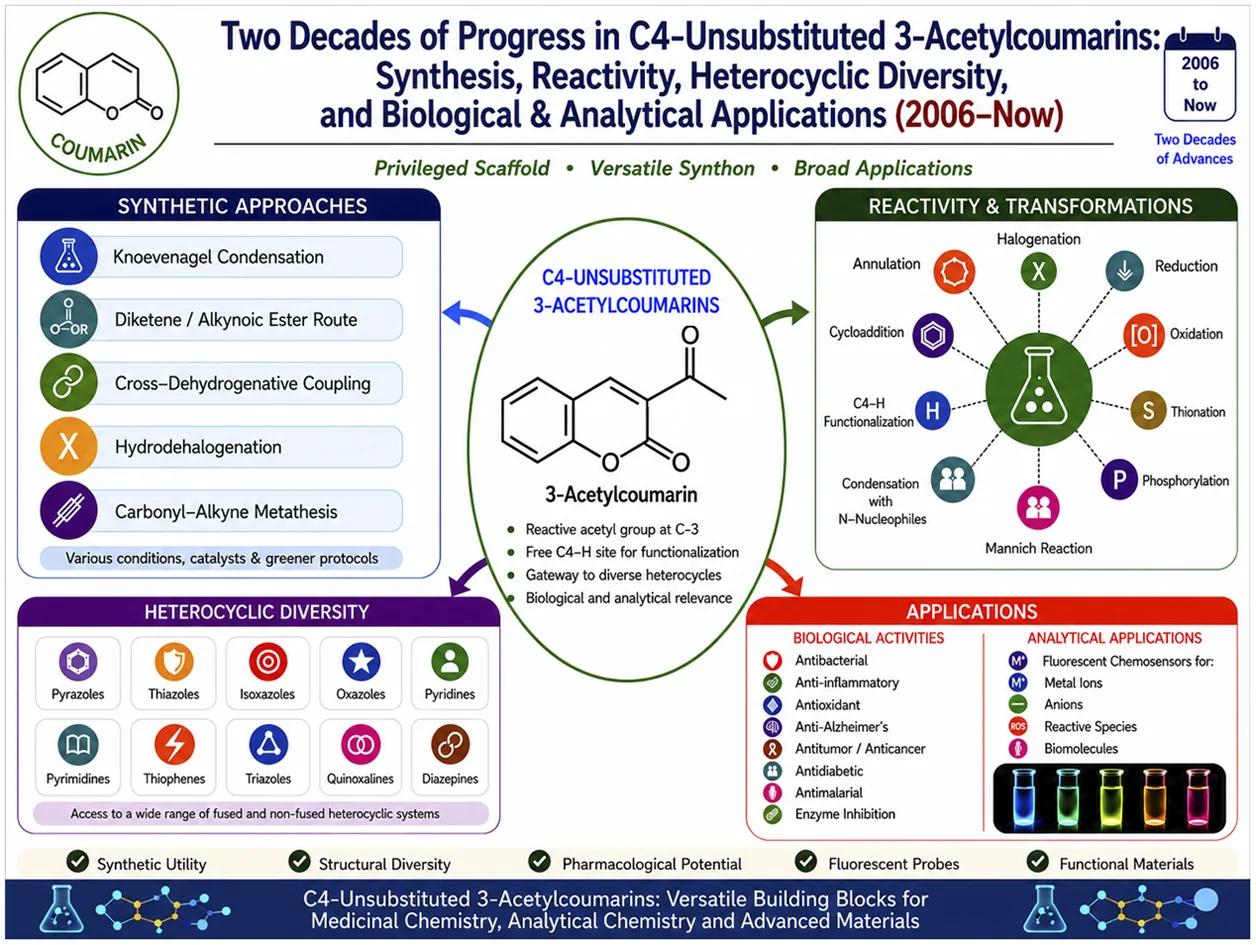

## 1. Introduction

Coumarins, widely recognized as benzopyranone derivatives, constitute one of the most important families of oxygen-containing heterocyclic compounds. Structurally, they consist of a benzene ring fused to a pyran-2-one nucleus, a framework that provides remarkable chemical versatility and broad functional value.^1–3^ These naturally occurring compounds are widely distributed in fungi, bacteria, fruits, and numerous edible and medicinal plants, including *Pastinaca sativa* and *Heracleum lanatum* seeds. To date, nearly 1300 coumarin derivatives have been isolated from natural sources, reflecting the structural richness and biological significance of this class.^4,5^ Owing to their diverse substitution patterns and tunable physicochemical properties, coumarins have become privileged scaffolds in natural products, pharmaceuticals, agrochemicals, fluorescent probes, synthetic dyes, and advanced functional materials.^6–12^

Among the different coumarin subclasses, 3-substituted coumarins bearing an unsubstituted C-4 position have attracted considerable attention. This structural arrangement preserves the reactive C4–H site, allowing further functionalization and enabling the construction of diverse molecular architectures. Moreover, this substitution pattern is frequently associated with valuable biological and chemical properties. Several pharmacologically important compounds, including warfarin, dicoumarol, phenprocoumon, pachyrrhizine, and AP2238, contain coumarin-based scaffolds and illustrate the medicinal relevance of this heterocyclic core, as shown in Fig. 1.^13–16^ Consequently, the development of efficient strategies for the synthesis and transformation of 3-substituted, C4-unsubstituted coumarins has remained an active and attractive research area in synthetic organic chemistry, medicinal chemistry, and materials science.^17–20^

**Fig. 1:**
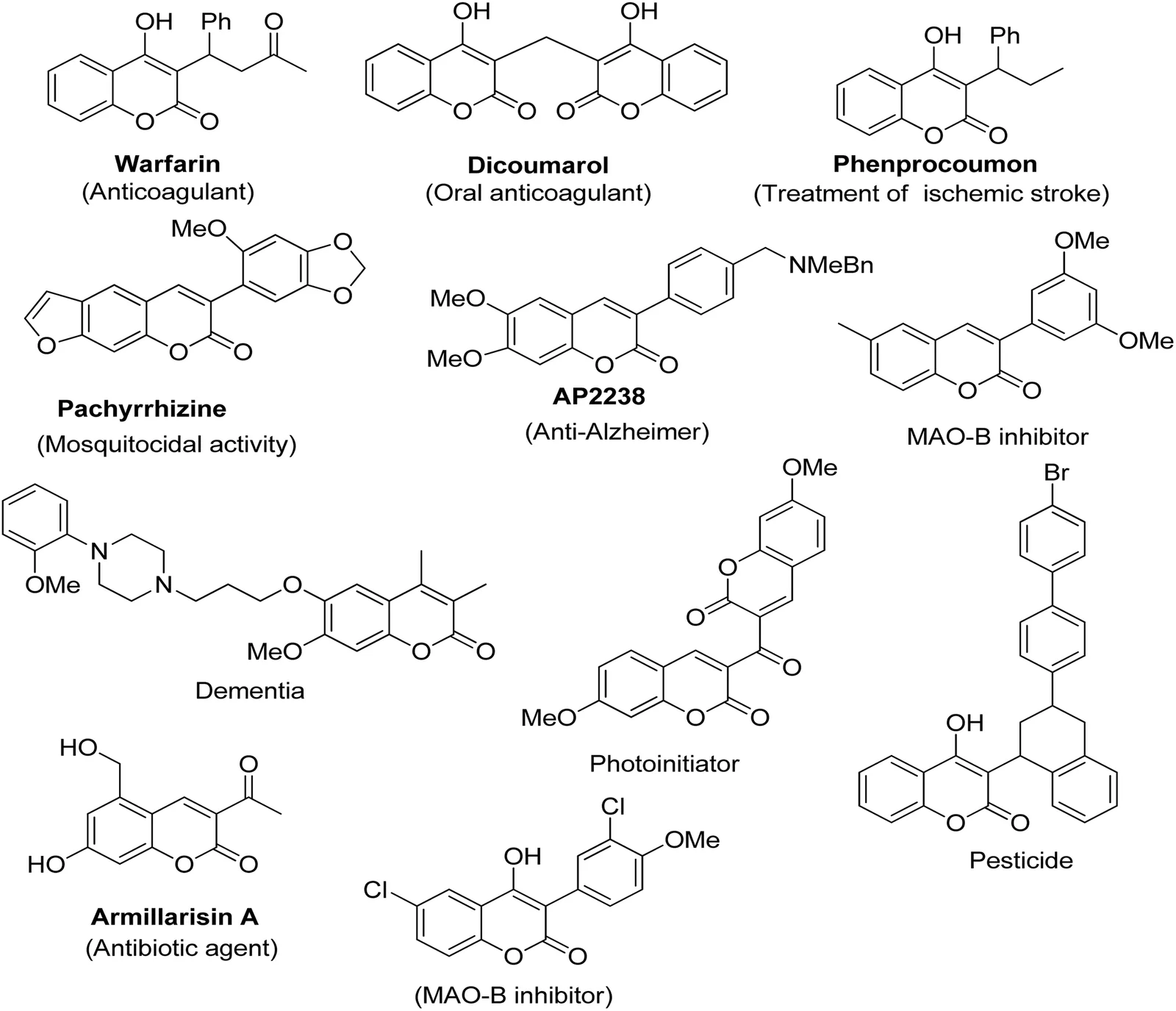
Representative coumarin-based scaffolds with pharmacological relevance, illustrating the structural diversity and medicinal importance of the coumarin nucleus in drug discovery and heterocyclic chemistry.

Within this important family, C4-unsubstituted 3-acetylcoumarins occupy a distinctive position because they combine the coumarin lactone system with a highly reactive acetyl functionality at the C-3 position. The presence of a free C4–H site further enhances their synthetic utility by enabling direct C–H functionalization, condensation, annulation, and cyclization reactions. This unique structural combination provides multiple electrophilic and nucleophilic reaction centers, making 3-acetylcoumarins versatile building blocks for the preparation of a wide variety of heterocyclic and fused heterocyclic systems.^21–23^ In addition to their synthetic importance, 3-acetylcoumarin derivatives have demonstrated promising pharmacological activities, including antibacterial, anti-inflammatory, antioxidant, anti-Alzheimer's, and enzyme-inhibitory effects.^24–28^ Furthermore, selected 3-acetylcoumarin-based derivatives have been employed as fluorescent chemosensors, such as boronic ester derivatives used for the detection of hydrogen peroxide in living cells, highlighting their potential in bioimaging, analytical chemistry, and medicinal diagnostics.^24^

The chemistry of 3-acetylcoumarins has enabled a broad range of transformations, including condensation, halogenation, reduction, oxidation, thionation, phosphorylation, Mannich reactions, reactions with nitrogen nucleophiles, C4–H functionalization, and heterocyclic annulation. Through these transformations, numerous structurally diverse coumarin-based systems have been accessed, including pyrazoles, thiazoles, oxazoles, isoxazoles, pyridines, pyrimidines, thiophenes, diazepines, benzoxocins, and other annulated frameworks.^25–31^ Accordingly, C4-unsubstituted 3-acetylcoumarins have emerged not only as biologically relevant scaffolds but also as strategic synthons for the design of functional molecules, fluorescent dyes, bioactive heterocycles, and advanced organic materials.

The rationale for focusing specifically on C4-unsubstituted 3-acetylcoumarins arises from their unique structural features and versatile synthetic potential. The coexistence of a reactive C3-acetyl group and an accessible C4–H position provides complementary reactive sites that enable diverse transformations, including selective C–H functionalization, condensation, cyclization, and heterocyclic annulation. These characteristics distinguish this scaffold from other coumarin subclasses and highlight its importance in modern synthetic, medicinal, and materials chemistry.

In continuation of previous studies on coumarin chemistry and related azo/thio isosteric analogues,^32–42^ the present review aims to provide a critical and systematic overview of advances in C4-unsubstituted 3-acetylcoumarin chemistry from 2006 to 2026. Although coumarin chemistry has been extensively investigated, no previous review has specifically focused on C4-unsubstituted 3-acetylcoumarins. Earlier reviews have mainly addressed general coumarin derivatives, 4-hydroxycoumarins, 3-bromoacetylcoumarins, or broader classes of 3-substituted coumarins, leaving this structurally distinctive and synthetically valuable subclass underrepresented. This gap is particularly significant because C4-unsubstituted 3-acetylcoumarins uniquely combine a reactive C-3 acetyl group with a free C4–H site, thereby enabling a wide range of transformations, including condensation, C–H functionalization, cyclization, and heterocyclic annulation.

To provide a comprehensive and well-organized account of this rapidly expanding field, a structured literature search was conducted covering publications from 2006 to 2026. The literature search was performed using SciFinder, Reaxys, Scopus, Web of Science, Google Scholar, and PubMed. Different combinations of keywords were employed, including “3-acetylcoumarin”, “3-acetyl-2*H*-chromen-2-one”, “C4-unsubstituted 3-acetylcoumarin”, “synthesis”, “reactions”, “heterocyclic synthesis”, “biological activity”, “fluorescence”, and related terms. Original research articles, review papers, and relevant book chapters were screened according to their relevance to the synthesis, chemical reactivity, structural characterization, heterocyclic transformations, biological evaluation, photophysical properties, and analytical applications of C4-unsubstituted 3-acetylcoumarins. Studies were included when they directly addressed the preparation, transformation, characterization, or application of the target scaffold, whereas publications in which 3-acetylcoumarins were only incidentally mentioned, insufficiently characterized, or structurally unrelated to the scope of this review were excluded. In addition, the reference lists of relevant original articles and review papers were manually examined to identify further pertinent publications that were not retrieved during the initial database search.

Accordingly, this review classifies the available literature according to synthetic strategy, reaction type, structural outcome, and application area. Particular emphasis is placed on synthetic methodologies, reactivity patterns, heterocyclic transformations, biological relevance, photophysical properties, and analytical applications. By linking synthetic developments with functional applications, this review provides a focused and useful reference for researchers working in synthetic organic chemistry, heterocyclic chemistry, medicinal chemistry, fluorescence sensing, and functional materials science.

## 2. Synthesis

### 2.1. Knoevenagel condensation of salicylaldehydes with β-keto esters

Knoevenagel condensation remains the most widely employed strategy for the synthesis of 3-acetylcoumarin derivatives 3a–af through the reaction of substituted salicylaldehydes 1 with β-keto ester derivatives 2. This transformation is commonly promoted by catalytic or stoichiometric bases, including piperidine, pyridine, triethylamine, diethylamine, piperidinium acetate, dimethylacetamide and DBU. In addition to these classical protocols, considerable attention has recently been directed towards greener and operationally simpler variants, such as heterogeneous catalysis, transition-metal catalysis, solid-supported methods, ionic-liquid-mediated reactions, solvent-free microwave irradiation and grinding techniques (Scheme 1).^43–157^ These approaches offer important advantages, including shorter reaction times, easier work-up procedures and reduced solvent consumption. However, many reported methods are still assessed mainly in terms of isolated yields, while aspects such as substrate scope, catalyst recyclability, scalability and environmental impact remain insufficiently discussed. Therefore, a more systematic comparison of these protocols is needed to clarify their practical value and guide future development of sustainable routes to C4-unsubstituted 3-acetylcoumarins.

**Scheme 1:**
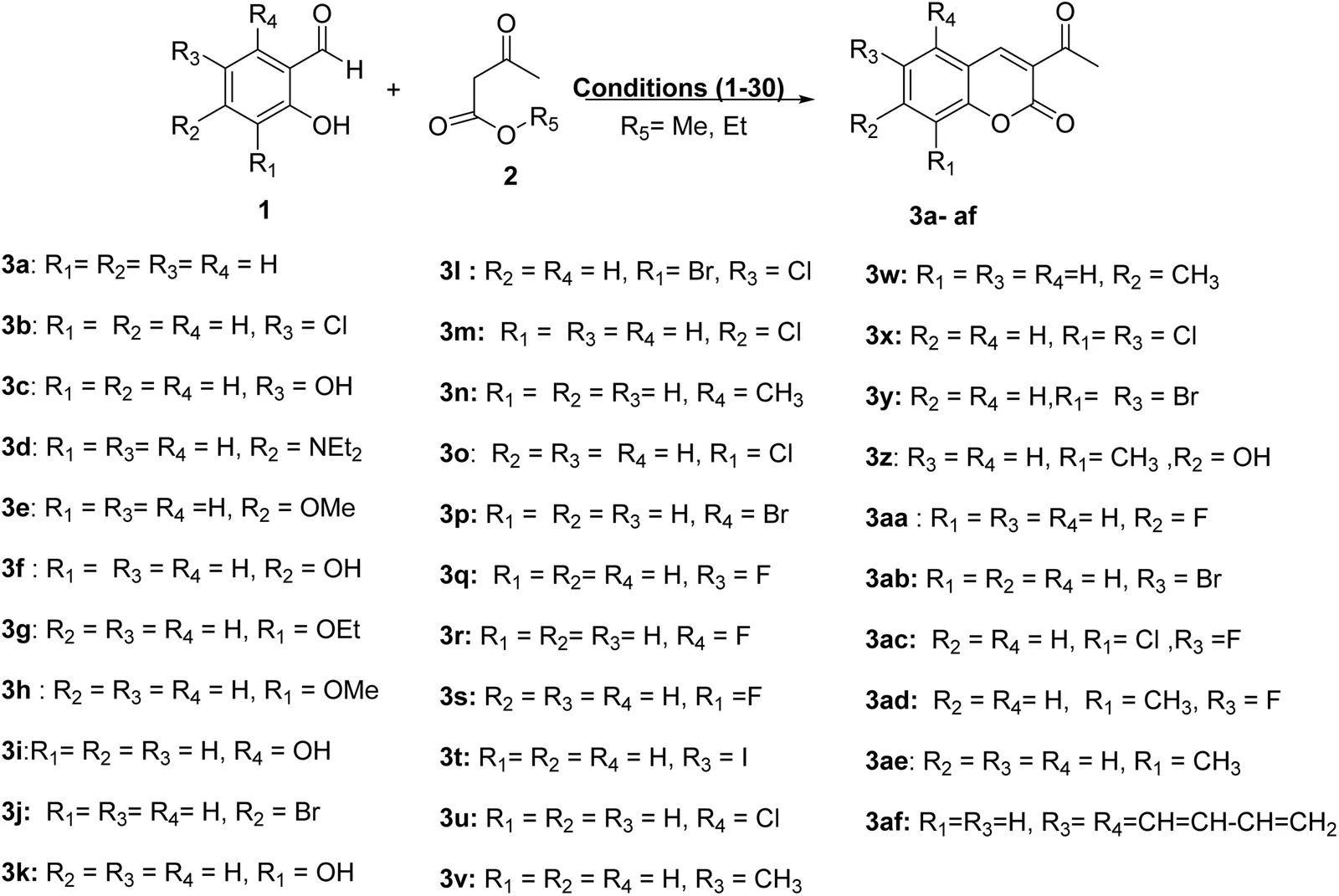
Representative synthetic approaches for the preparation of C4-unsubstituted 3-acetylcoumarins 3a–af through Knoevenagel condensation of salicylaldehydes 1 with β-keto ester derivatives 2.

As shown in Table 1, the Knoevenagel route has evolved from conventional base-mediated protocols towards greener and more operationally attractive methods, including solvent-free reactions, aqueous media, organocatalysis, heterogeneous catalysis and microwave-assisted synthesis. However, direct comparison among these methods remains difficult because several reports emphasise isolated yields while providing limited information on substrate scope, catalyst recyclability, scalability and environmental performance.

**Table 1:** Representative conditions reported for the Knoevenagel synthesis of C4-unsubstituted 3-acetylcoumarins 3a–af

Entry	Catalyst/base system	Solvent/medium	Temperature	Time	Methodological note	Ref.
1	Piperidine	Solvent-free	RT/reflux	2–3 h	Classical base-mediated protocol; solvent-free variant	43–76
2	Piperidine	EtOH	RT/reflux	24 h	Conventional and widely used ethanolic protocol	77–114
3	Piperidine	MeOH	RT	1 h	Mild alcoholic medium	115–118
4	Piperidine	AcOH/EtOH	Reflux	6 h	Acidic ethanolic medium	119–125
5	Piperidine	EtOAc	130–140 °C	9 min	High-temperature protocol	126–128
6	Piperidine	DMF	RT	1 h	Mild and relatively rapid reaction	129
7	Piperidine	DCM	RT	4 h	Mild chlorinated-solvent protocol	130
8	l-Proline	Solvent-free or triethanolamine	RT	0.5 h	Organocatalytic and operationally simple method	131–134
9	DMA	H_2_O/EtOH	RT	10 h	Aqueous/alcoholic medium	135 and 136
10	Pyridine	DMA	0–10 °C	3 h	Low-temperature protocol	137
11	NaOH	EtOH	MW	10 min	Microwave-assisted rapid synthesis	138
12	Fe_3_O(BPDC)_3_/*t*-BuOOH	H_2_O	60 °C	180 min	Transition-metal-based catalytic system	139
13	Fe_3_O_4_-DA-Phe	Solvent-free	130 °C	6–10 min	Magnetic/reusable catalyst under microwave irradiation	140
14	ZrOCl_2_·8H_2_O	Solvent-free	RT	Milling	Solvent-free mechanochemical protocol	141
15	2-PP-SDS	H_2_O	30 °C	6 h	Aqueous surfactant-assisted method	142
16	Bi(NO_3_)_3_–Al_2_O_3_/ZnCl_2_	Solvent-free	120 °C	30 min	Heterogeneous Lewis-acid system	143
17	K_2_CO_3_/[bmim]Br	Ionic-liquid medium	MW	1.5 min	Ionic-liquid-mediated microwave protocol	144
18	ZnO	Solvent-free	MW or thermal	6 min	Rapid catalyst-assisted method	145
19	Taurine	H_2_O	Reflux	65–85 min	Aqueous amino-acid-derived promoter	146
20	Triethanolamine/l-proline	Solvent-free	RT	0.5 h	Combined base/organocatalytic system	147
21	Pyridine	EtOH	RT	16.5 h	Mild conventional protocol	148
22	Piperazine	Solvent-free	25–30 °C	1 h	Mild amine-promoted method	149
23	l-Lysine	H_2_O	60 °C	6 h	Aqueous organocatalytic protocol	150
24	PS-TBD	Solvent-free	30 °C	10 h	Polymer-supported base; solvent-free conditions	151
25	Thiourea dioxide	Solvent-free	80 °C	3.5 h	Alternative organosulfur-based promoter solvent-free	152
26	*rac*-Pro-OH	Solvent-free	60 °C	1–3 h	Organocatalytic protocol	153
27	InCl_3_	EtOH	80 °C	6.5 h	Lewis-acid-catalysed method recyclable	154
28	Amberlyst 26A	EtOH	Reflux	30 min	Heterogeneous resin catalyst	155
29	PhI(OAc)_2_	EtOH	35–40 °C	0.5–1 h	Hypervalent iodine-mediated protocol	156
30	KF–Al_2_O_3_	Solid-supported medium	MW	6–9 h	Solid-supported microwave-assisted method	157

Using a modified Knoevenagel approach, 3-acetylcoumarin 3 was prepared from salicylaldehyde 1 and polymer-supported acetoacetate reagent 4 in the presence of piperidine as the catalyst. The reaction proceeded through intramolecular transesterification involving the phenolic group, followed by elimination of the polymer-bound hydroxyl terminus to afford the desired product (Scheme 2).^158–160^

**Scheme 2:**
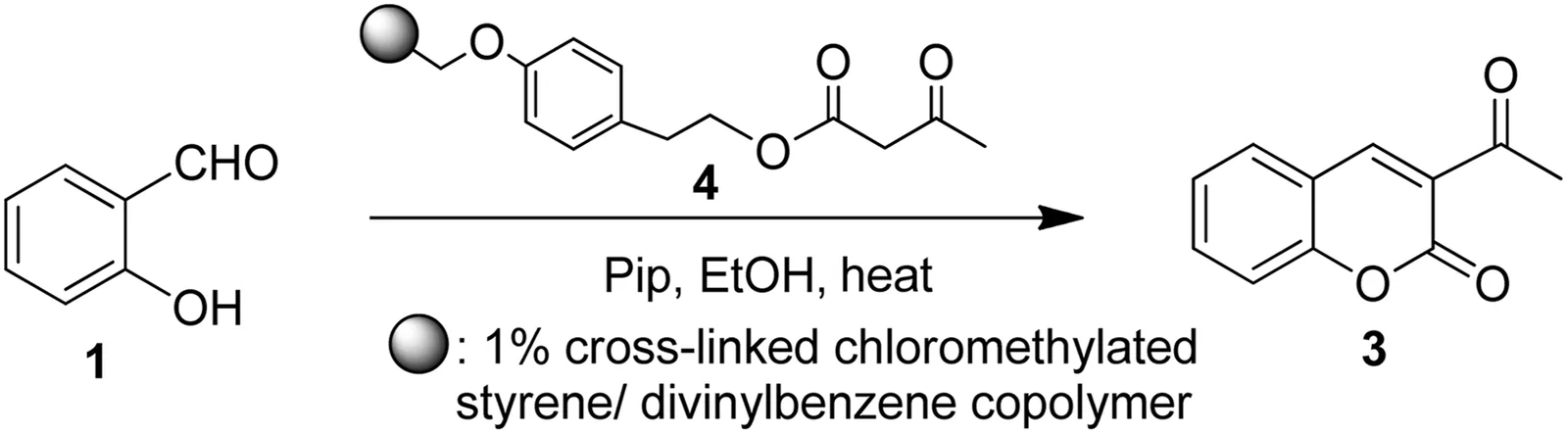
Synthesis of 3*via* polymer-supported.

### 2.2. Synthesis from salicylaldehydes and substituted acetoacetanilides

Another elegant approach to attain acetylcoumarin derivatives 3a–d was straightforward, one-pot reaction of salicylaldehydes 1 and acetoacetanilide 5 in DMF at room temperature catalyzed by 1,4-diazabicyclo[2.2.2]octane (DABCO)/tetrabutylammonium bromide (TBAB) system (Scheme 3).^161^

**Scheme 3:**
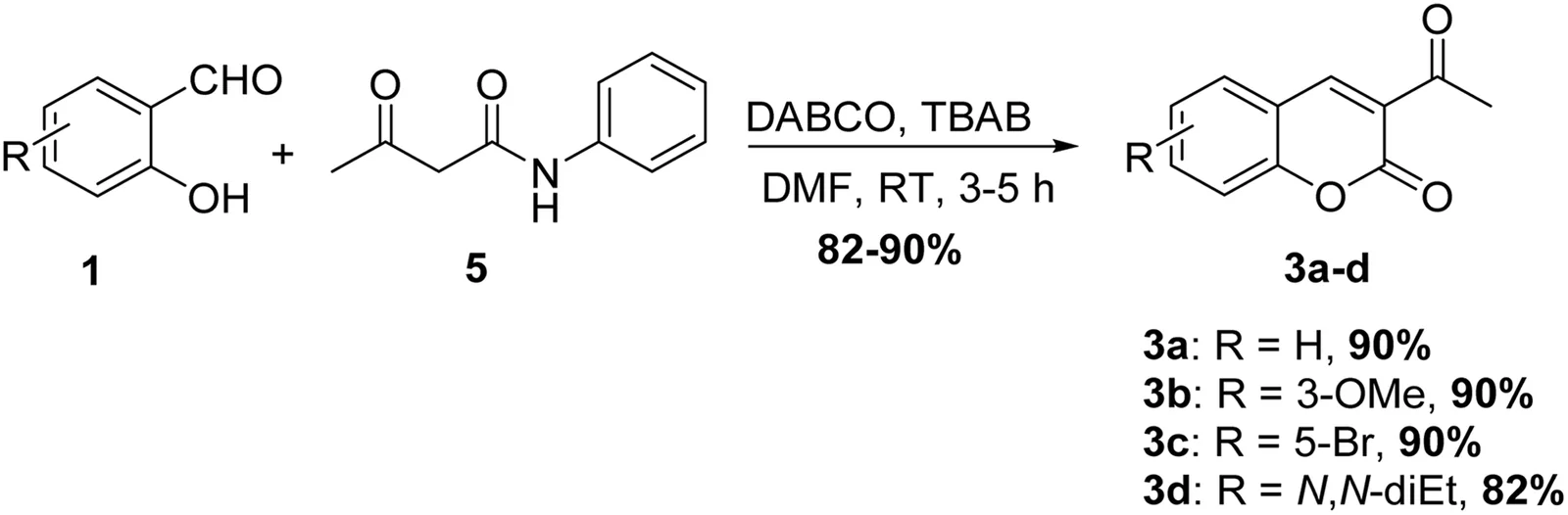
DABCO/TBAB-mediated synthesis of 3-acetylcoumarin derivatives 3a–d in DMF.

### 2.3. Diketene-mediated synthesis

An alternative route to 3-acetylcoumarins 3a, b was achieved *via* titanium tetraisopropoxide-promoted Knoevenagel condensation of salicylaldehydes 1 with diketene 6, followed by spontaneous Pinner cyclization in i-PrOH (Scheme 4).^162^

**Scheme 4:**
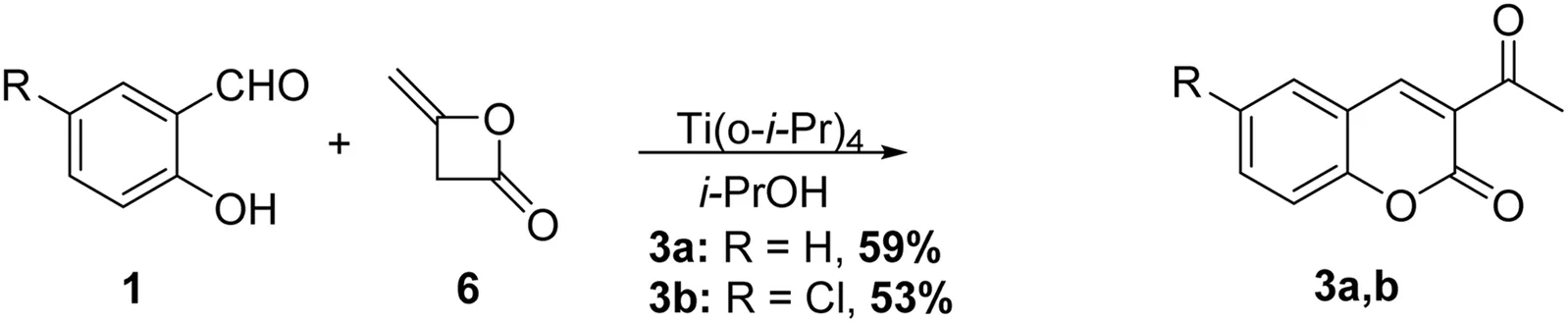
Condensation of salicylaldehyde 1 with diketene 6 to afford 3-acetylcoumarin 3.

### 2.4. Cross-dehydrogenative coupling approaches

Adib *et al.*^163^ reported the regioselective synthesis of 3-acetylcoumarin 3*via* dehydrogenative cross-coupling of parent coumarin 7 with acetaldehyde 8 catalyzed using a catalytic mixture of dipotassium peroxodisulfate and Aliquat 336 in chlorobenzene at 100 °C for 8 h (Scheme 5).

**Scheme 5:**
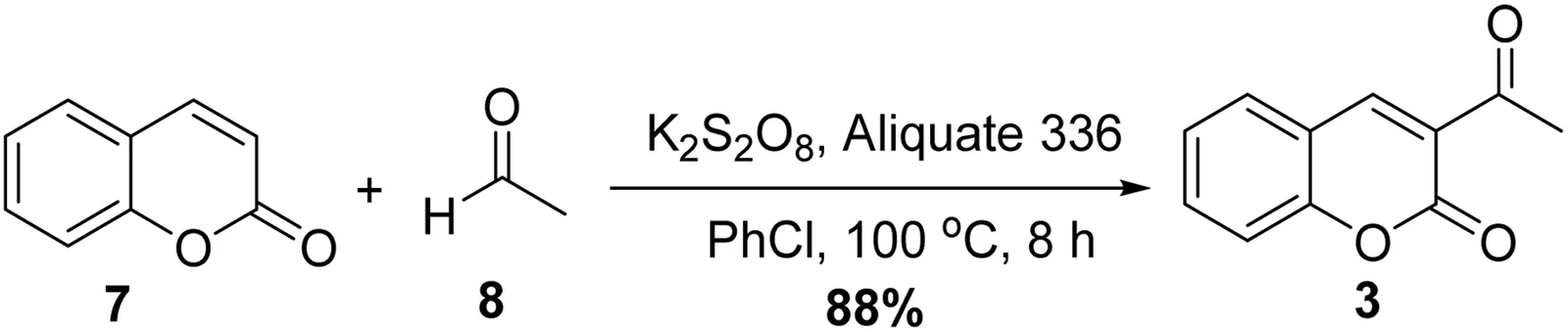
Dipotassium peroxodisulfate as a catalyst for the synthesis of 3-acetylcoumarin.

### 2.5. Synthesis *via* CuI-catalysed hydrodehalogenation of 4-chloro-3-acetylcoumarins

Series of acetylcoumarins 3a–f was achieved *via* copper(i)iodide catalyzed the regioselective hydrodehalogenation of 4-chloro-3-acetylcoumarins 9 in isopropanol under basic condition (Scheme 6).^164^

**Scheme 6:**
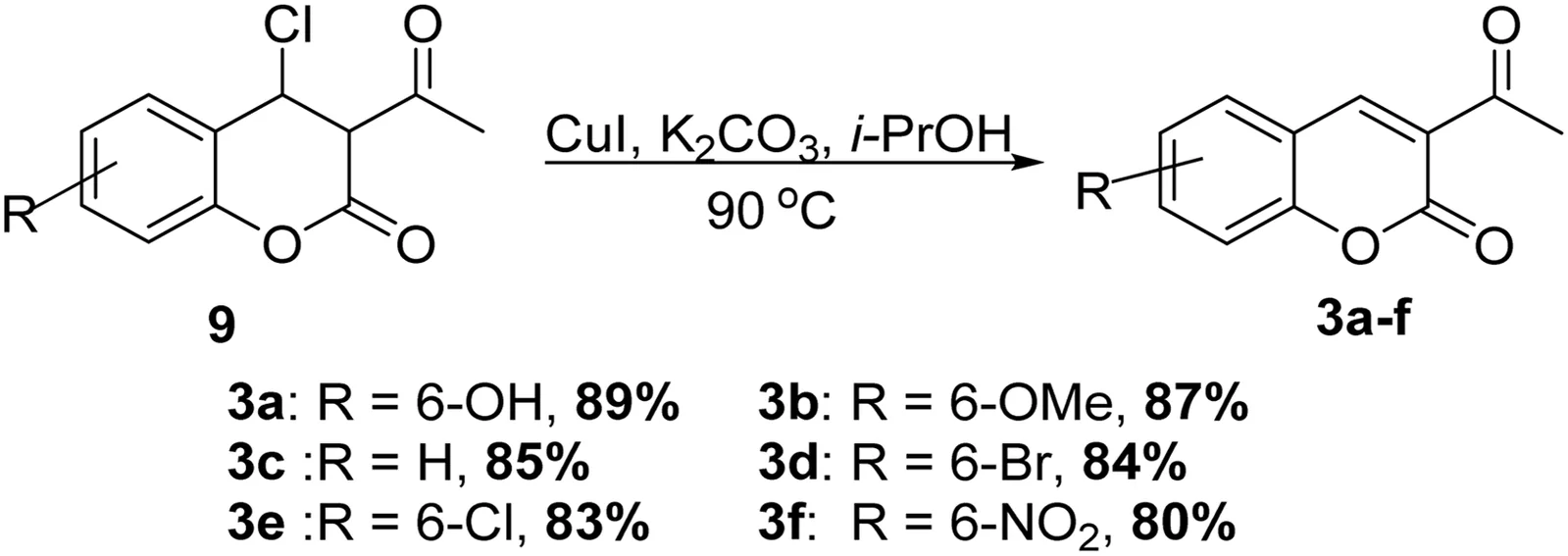
Synthesis of 3-acetylcoumarins 3 from 4-chloro-3-acetylcoumarins 9.

### 2.6. Direct synthesis from alkynoic esters

An alternative direct approach to acetylcoumarin derivatives 3a–k involves reacting salicylaldehydes 1 with ethyl but-2-ynoate 10 in acetonitrile at 75 °C. The process uses a dual organocatalytic system composed of *p*-toluenesulfonic acid and piperidine, proceeding through an enamino ester intermediate. This metal-free method affords the target products in good yields with high selectivity under relatively mild conditions (Scheme 7).^165^

**Scheme 7:**
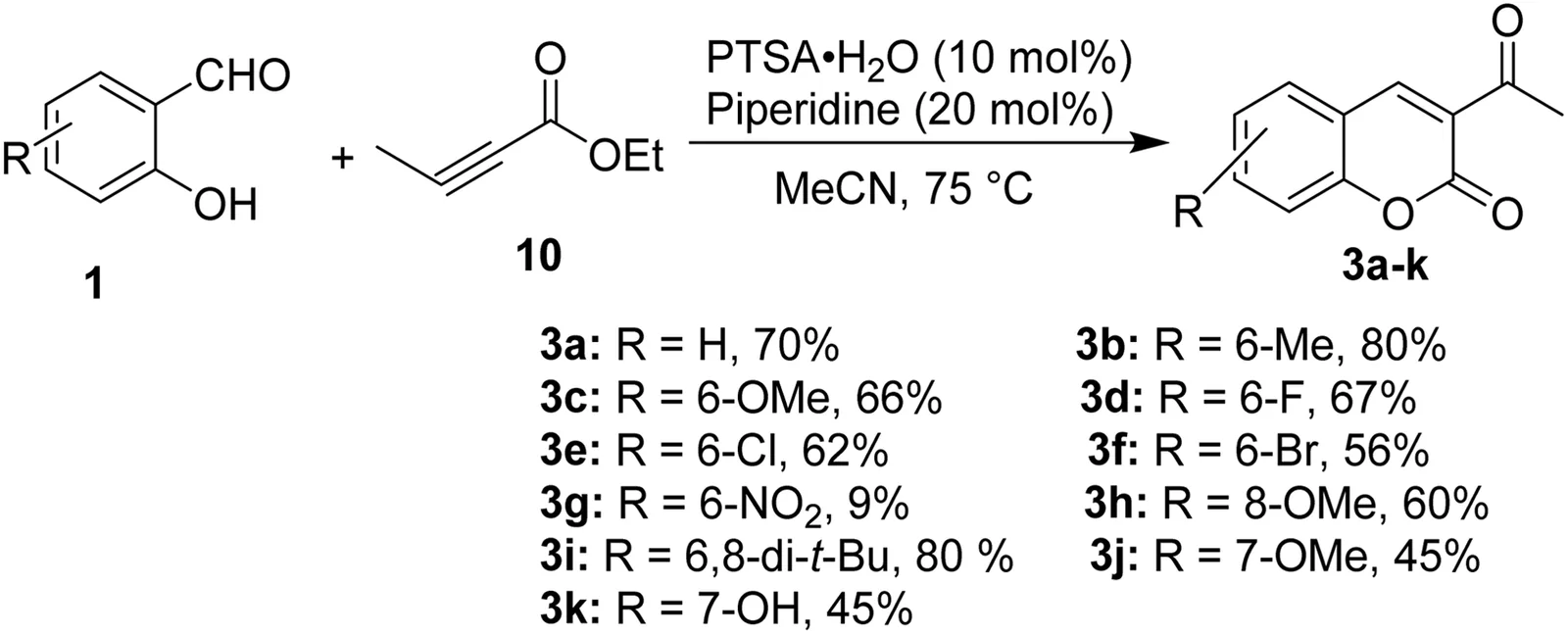
Direct organocatalytic synthesis of coumarin from alkynoic esters.

### 2.7. TMSOTf-catalyzed carbonyl–alkyne metathesis

Trimethylsilyl trifluoromethanesulfonate (TMSOTf) efficiently catalyzes intramolecular and intermolecular carbonyl-alkyne metathesis of 2-formylphenyl but-2-ynoate 11 in 1,1-dichloroethylene (DCE), giving acetylcoumarin 3 in moderate yield (Scheme 8).^166^

**Scheme 8:**
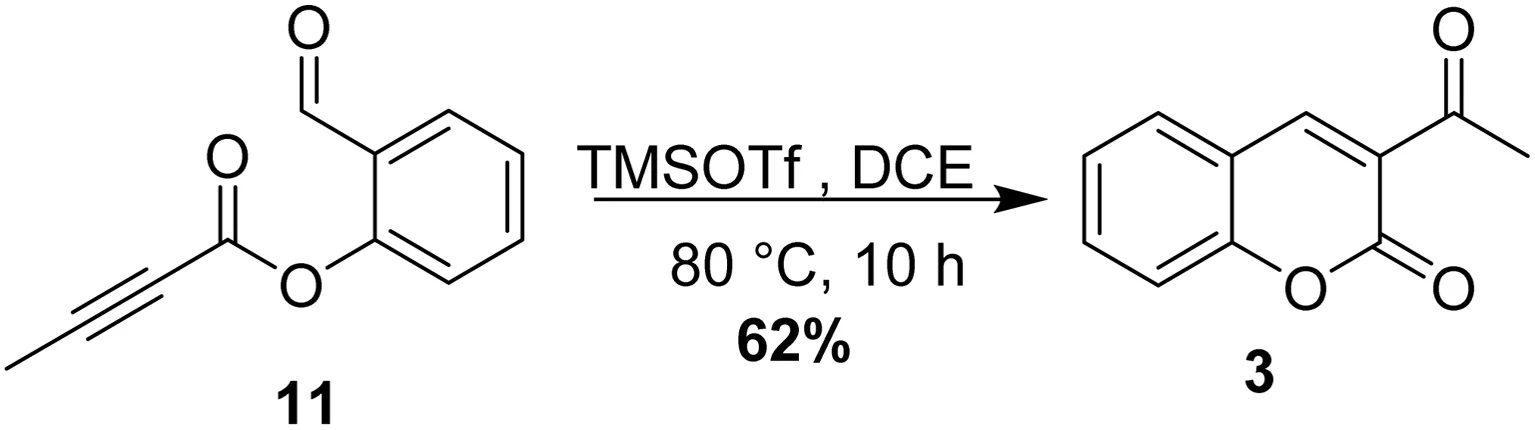
Preparation of 3 through a TMSOTf-promoted carbonyl–alkyne metathesis reaction.

## 3. Reactivity patterns, spectroscopic characteristics, and structural features

### 3.1. Reactivity centres of 3-acetylcoumarins

The characteristic reactivity of 3-acetylcoumarins originates from the conjugation of the α,β-unsaturated lactone system with the C3-acetyl substituent. The two carbonyl groups strongly polarize the π-system through inductive and resonance effects, generating several electronically differentiated reaction centres. The lactone carbonyl carbon (C-2) and the acetyl carbonyl carbon (C-9) are intrinsically electrophilic because of polarization of the corresponding C=O bonds. In addition, conjugation within the α,β-unsaturated coumarin system renders C-4 electron-deficient, accounting for its susceptibility to nucleophilic or radical addition and its participation in several regioselective C4-functionalization reactions.

In contrast, the carbon atoms adjacent to the carbonyl functions may acquire nucleophilic character through resonance or enolization. In particular, the methyl group of the C3-acetyl substituent (C-10) contains acidic α-hydrogens and can generate an enol or enolate-type nucleophilic centre under basic or catalytic conditions. This behavior accounts for its participation in condensation, Mannich, and related carbon–carbon bond-forming reactions. The C-3 position is also involved in extensive π-conjugation between the lactone and acetyl functionalities, and its electron density can be redistributed through resonance depending on the reaction conditions. Therefore, the designation of individual centres as electrophilic or nucleophilic should be considered in the context of resonance, tautomerization, and the reaction medium rather than as fixed localized charges.

This electronic model is consistent with the experimentally observed chemistry summarized in the following sections: electrophilic carbonyl centres undergo reactions with nitrogen and oxygen nucleophiles, the acetyl methyl group participates in condensation and Mannich-type transformations after enolization, and the accessible C4–H position undergoes regioselective acylation, arylation, alkenylation, and other C–H functionalization processes. Thus, the broad synthetic versatility of C4-unsubstituted 3-acetylcoumarins can be rationalized by the interplay between carbonyl polarization, π-conjugation, resonance stabilization, and enolization of the C3-acetyl group (Fig. 2).

**Fig. 2:**
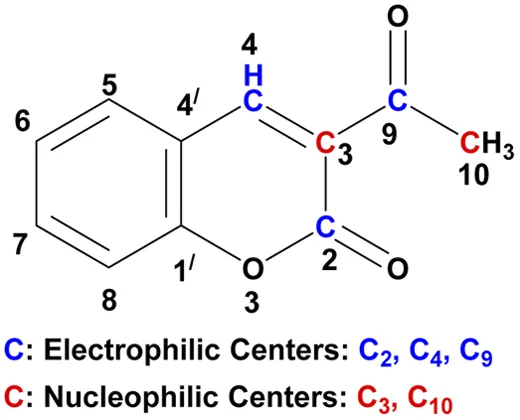
Reactivity centers of 3-acetylcoumarin 3.

### 3.2. Spectroscopic and crystallographic characterisation

The structure of C4-unsubstituted 3-acetylcoumarin 3 has been established by complementary spectroscopic methods rather than by relying on individual spectral values. In the ^1^H NMR spectrum, the acetyl methyl group appears as a characteristic singlet in the upfield region, while the isolated H-4 proton of the coumarin ring gives a diagnostic downfield singlet. For example, 3-acetylcoumarin has been reported to display signals at *δ* 1.98 ppm for the COCH_3_ group and *δ* 8.55 ppm for H-4, together with the four aromatic protons in the *δ* 7.33–7.91 ppm region. The simultaneous observation of the acetyl methyl resonance and the characteristic H-4 signal is consistent with formation of the C3-acetylated coumarin framework.^167^

Further structural support is provided by IR and ^13^C NMR spectroscopy. The IR spectrum exhibits two distinct carbonyl absorptions attributable to the conjugated acetyl ketone and the coumarin lactone functions. Reported spectra of 3-acetylcoumarin show strong carbonyl absorptions in the approximately 1705–1759 cm^−1^ region. In the ^13^C NMR spectrum, the acetyl carbonyl carbon appears strongly deshielded at approximately *δ* 197 ppm, whereas the lactone carbonyl resonates at approximately *δ* 157–159 ppm. These two chemically distinct carbonyl resonances provide direct evidence for the coexistence of the C3-acetyl group and the coumarin lactone system.^48^

Mass spectrometric data provide additional confirmation of the molecular composition. The molecular ion of 3-acetylcoumarin (C_11_H_8_O_3_) has been reported at *m*/*z* 188, consistent with its molecular mass and supporting the proposed elemental composition.^167^ Thus, the combined ^1^H NMR, ^13^C NMR, IR, and mass-spectrometric data establish the presence of the acetyl substituent, the intact α,β-unsaturated lactone system, and the expected coumarin aromatic framework.^167^

Photophysical behavior is strongly dependent on substitution of the coumarin chromophore and therefore should not be considered a primary criterion for structural assignment. Studies of 3-acetylcoumarin derivatives have shown that electron-donating substituents, particularly at C-7, can enhance intramolecular charge-transfer character and markedly influence fluorescence emission. Notably, the unsubstituted 3-acetylcoumarin analogue has been reported to show little or no detectable emission under some solution conditions, whereas appropriately substituted derivatives display substantially stronger fluorescence.^168^

Crystallographic evidence further supports the structural features of this class. Single-crystal X-ray diffraction of 3-acetyl-6-chloro-2*H*-chromen-2-one revealed a nearly planar coumarin system, with the acetyl group forming only a small dihedral angle with the heterocyclic framework. The crystal structure is additionally stabilized by intermolecular C–H⋯O contacts and π–π interactions (Fig. 3).^169^

**Fig. 3:**
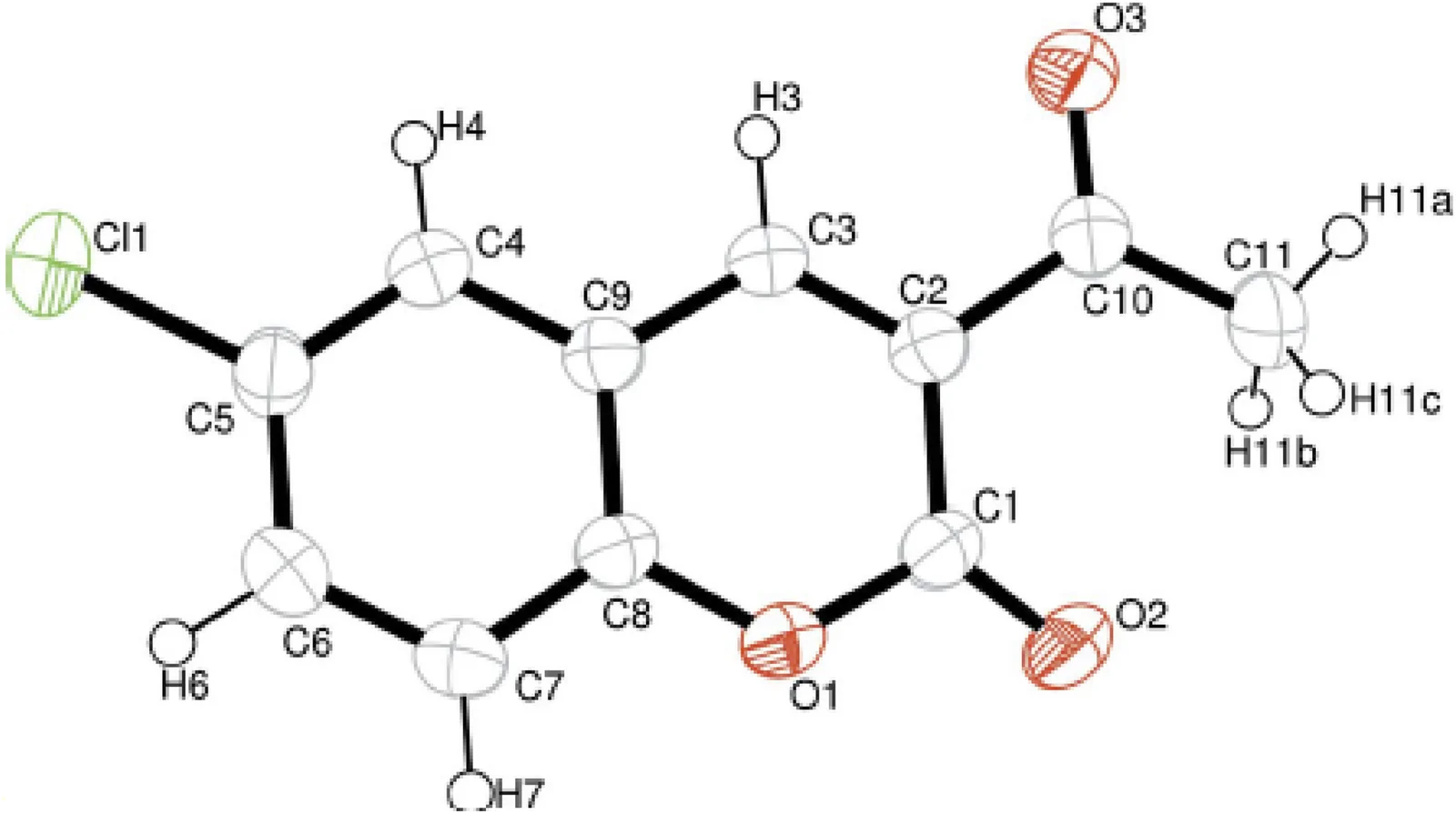
ORTEP diagram of 3-acetyl-6-chlorocoumarin at 50% probability level. [Reprinted from ref. 169].

## 4. Chemical transformations of C4-Unsubstituted 3-acetylcoumarins

### 4.1. Ring-opening reaction

Oxidative ring cleavage of 3-acetylcoumarin 3 was employed using carbon quantum dots (CQDs) to give salicylaldehyde 1 in a moderate yield (Scheme 9).^170^

**Scheme 9:**
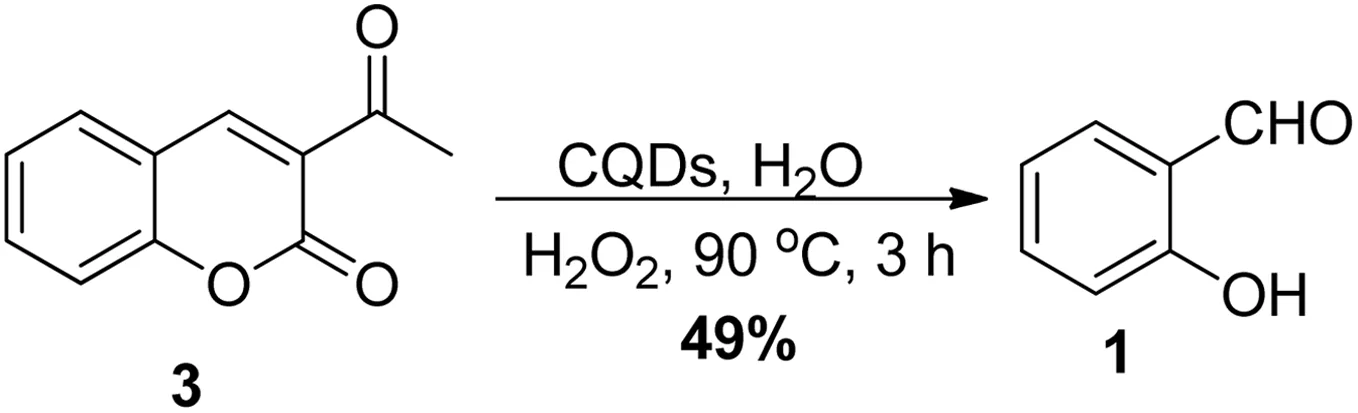
Various ring-opening transformations of acetylcoumarin 3.

### 4.2. C4-carbonylation and acylation reactions

Transition metal-free cross-dehydrogenative coupling C_4_–H acylation of acetylcoumarins 3 with aldehydes 8 as acylating agents at moderate temperature using K_2_S_2_O_8_/Aliquat 336 catalytic system has been reported as a versatile and economical approach towards 4-aroylcoumarin derivatives 12 (Scheme 10).^171^

**Scheme 10:**
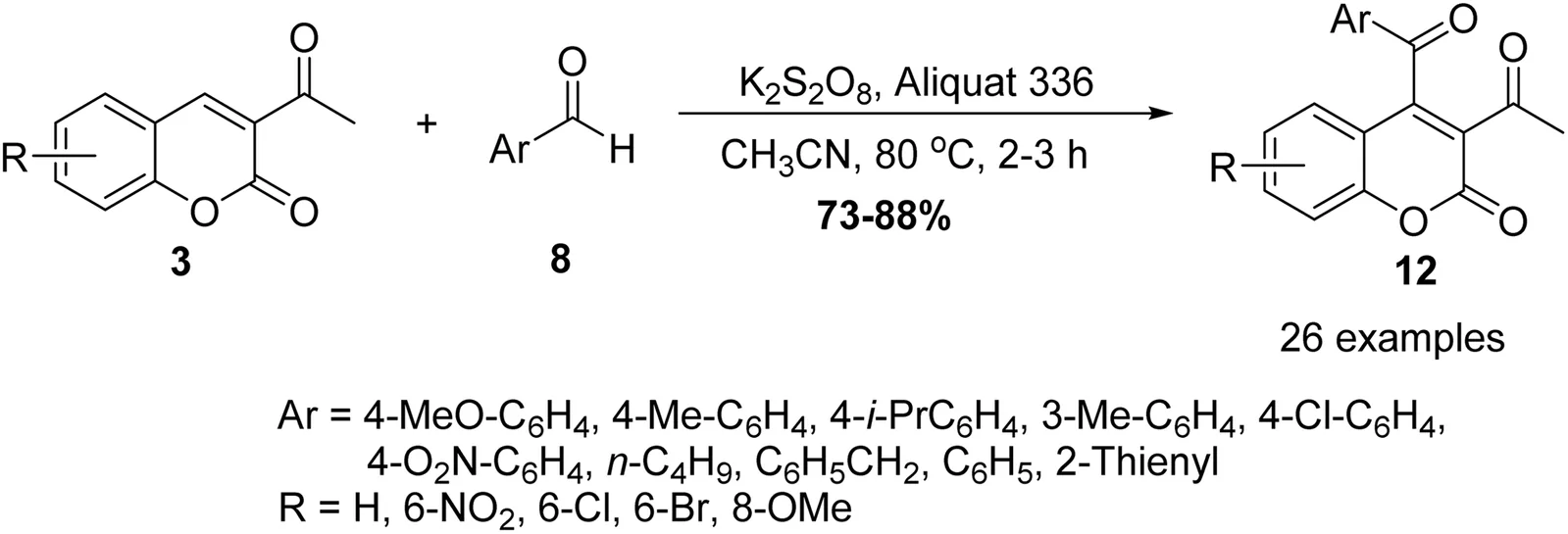
Treatment of acetylcoumarins 3 with aldehyde derivatives 8.

Iodine promoted selective oxidative cross coupling of 3 and imidazo[1,2-*a*]pyridine 13 for construction of imidazo[1,2-*a*](pyridin-3-yl)ethane-1,2-diones 14 with a new C–C bond formation *via* using *p*-toluene sulphonic acid (*p*-TSA) in DMSO can be envisioned in (Scheme 11).^172^

**Scheme 11:**
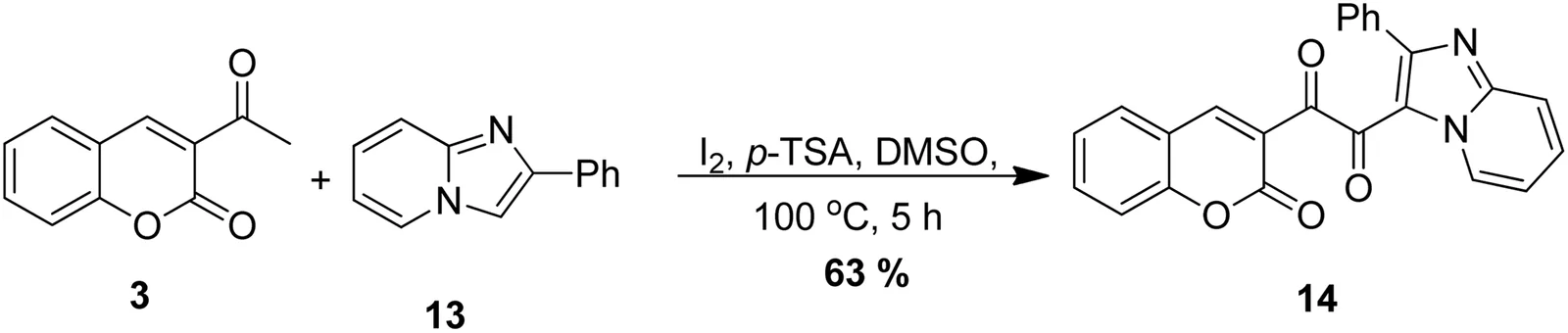
Selective oxidative cross-coupling of acetylcoumarins 3.

Alternatively, a highly efficient and selective palladium-catalyzed carbonylation of 3-acetylcoumarins 3 was allowed for the transformation of aryl halides 15 into the corresponding aryl coumarin ketones 17a–q under atmospheric carbon monoxide 16 pressure in the presence of AgOAc as a driving force (Scheme 12).^173^

**Scheme 12:**
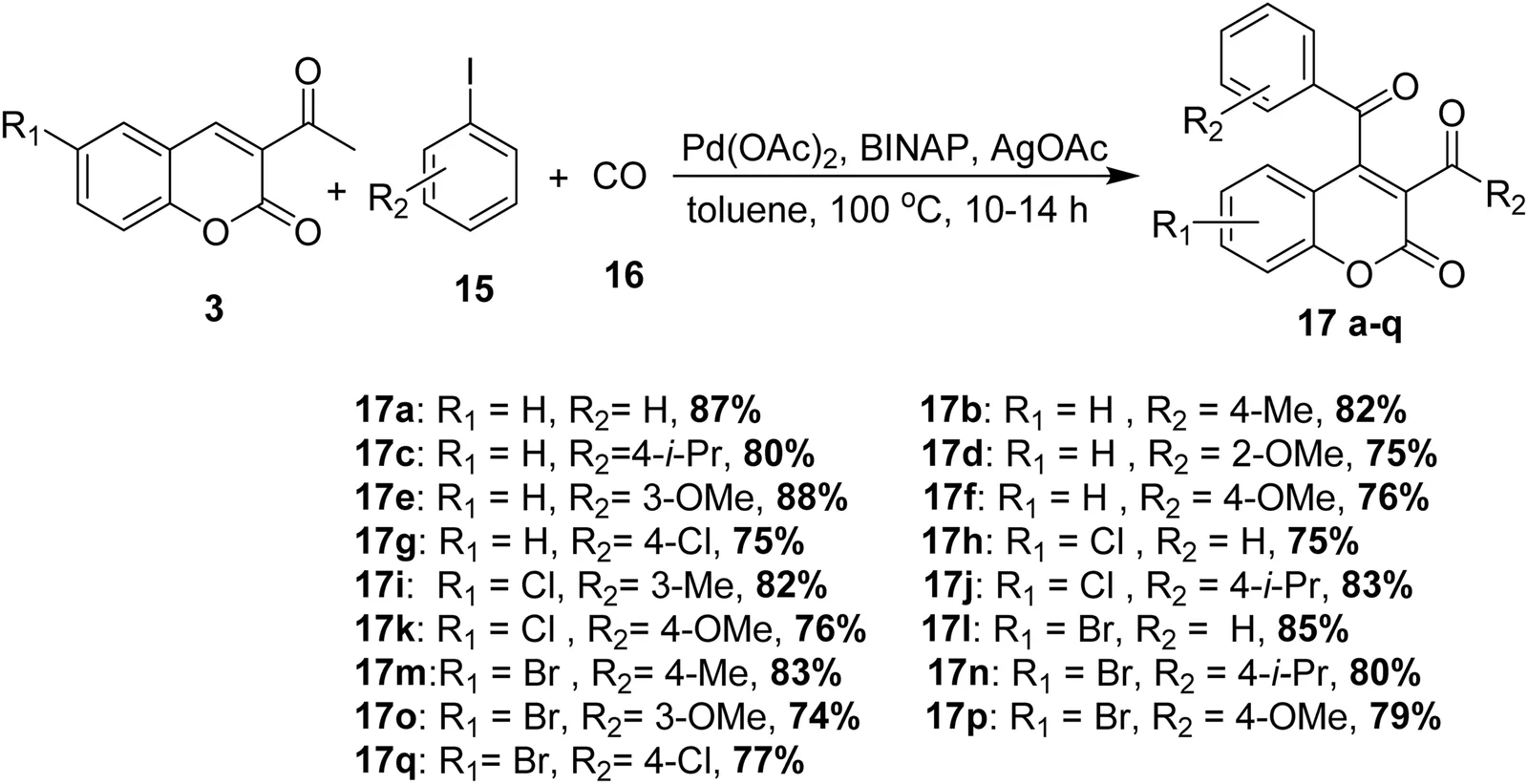
The transformation of 3 into the corresponding aryl coumarin ketones 17a–q.

### 4.3. Homodimerization reactions

3-Acetylcoumarin derivatives 3 undergo ultrasound-promoted homodimerization under Zn/chloroacetic anhydride or Zn/Zn(OAc)_2_ conditions, forming biscoumarin products 18*via* a radical pathway (Scheme 13).^174^

**Scheme 13:**
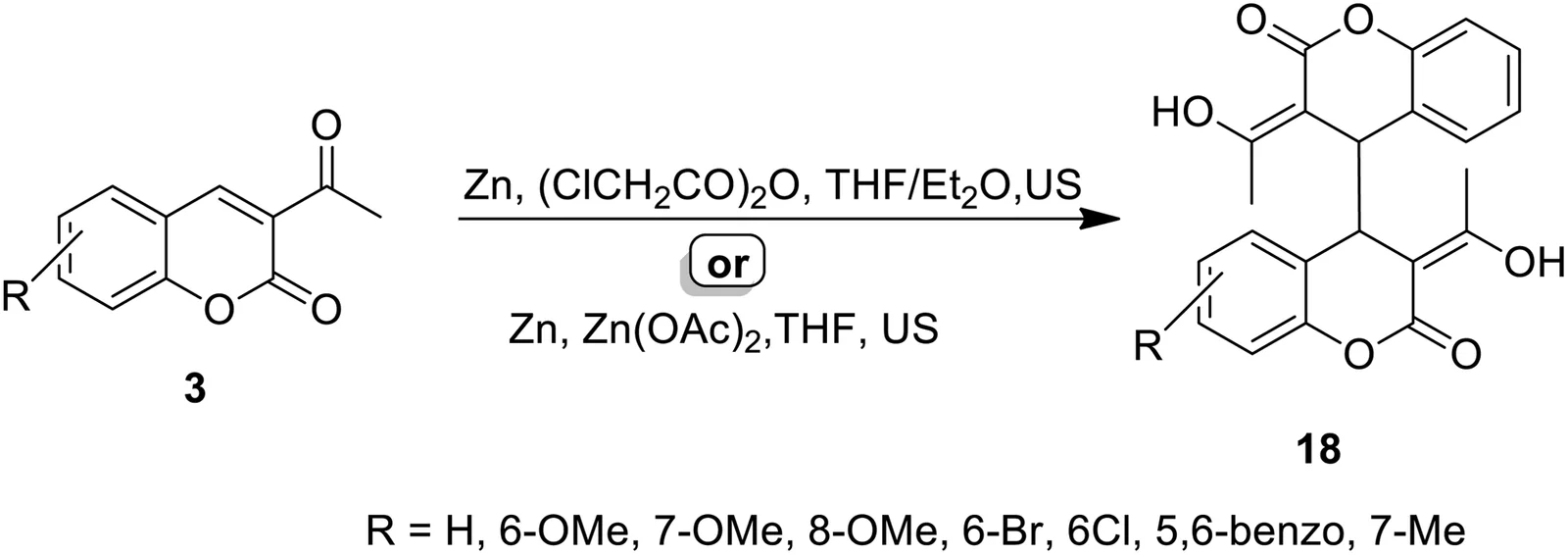
Ultrasound-promoted homodimerization of 3 to biscoumarin derivatives 18.

### 4.4. Indolylation reactions

FeCl_3_ catalyzed a highly regioselective 1,2-addition/substitution reaction between 3 and indoles 19, affording coumarin-substituted bis(indolyl)methanes 20 in yields ranging from 56 to 99% (Scheme 14).^175^

**Scheme 14:**
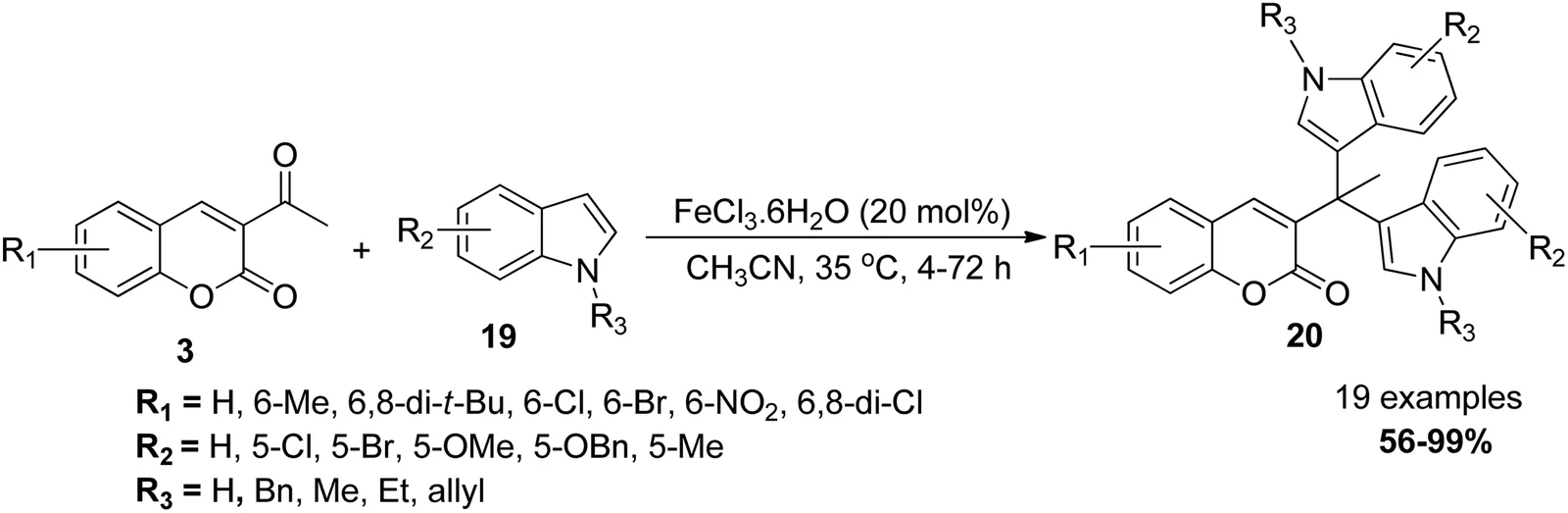
Indolylation of 3-acetylcoumarins 3.

### 4.5. C_4_-selective arylation reaction

Yuan *et al.*^176^ developed a mild, metal-free KMnO_4_/AcOH-mediated dehydrogenative radical arylation of 3 with arylboronic acids 21 to afford 3-arylcoumarins 22 (Scheme 15), providing an alternative to Pd-catalyzed Suzuki/Heck methods. Similarly, Banik *et al.*^177^ reported a visible-light-induced, metal-free arylation of coumarins 3 with arylboronic acids in MeOH/H_2_O (25 °C) under an inert atmosphere, using (NH_4_)_2_S_2_O_8_ as the oxidant and rose bengal as the photocatalyst (Scheme 15).

**Scheme 15:**
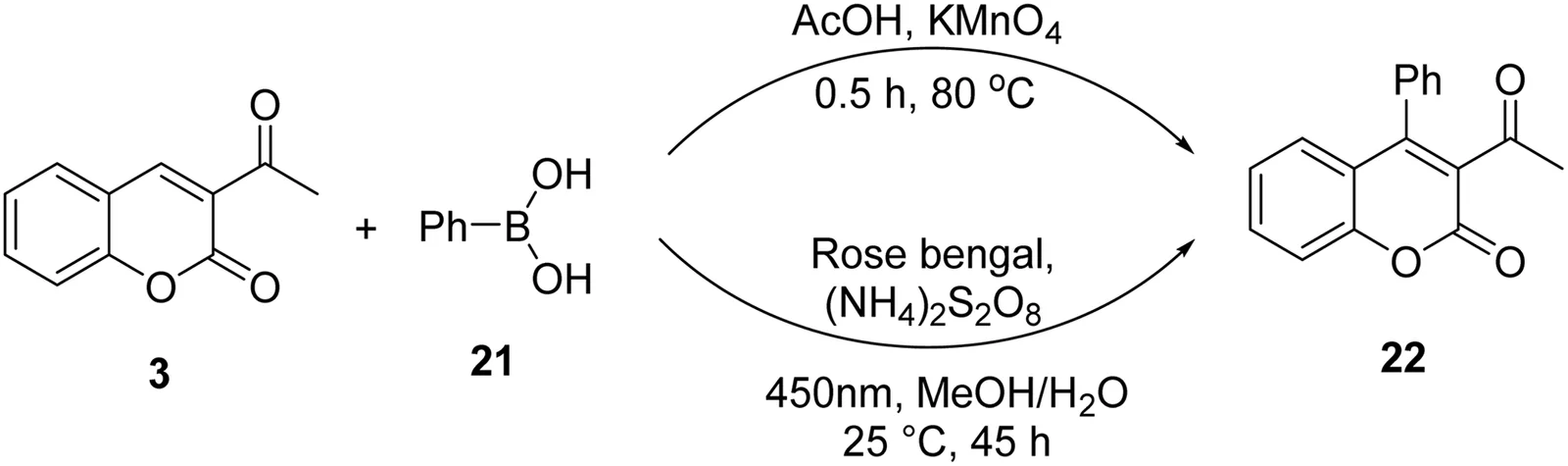
Treatment of 3-acetylcoumarin 3 with aryl boronic acid 21.

### 4.6. C_4_-alkenylation reaction

The triphenylphosphine catalyzed proton transfer process reaction of 3-acetylcoumarins 3 to dimethyl acetylenedicarboxylates 23 was achieved in dichloromethane producing the desired functionalized 3-acetyl-4-(substituted ethylenyl)coumarins 24 (Scheme 16).^178^

**Scheme 16:**
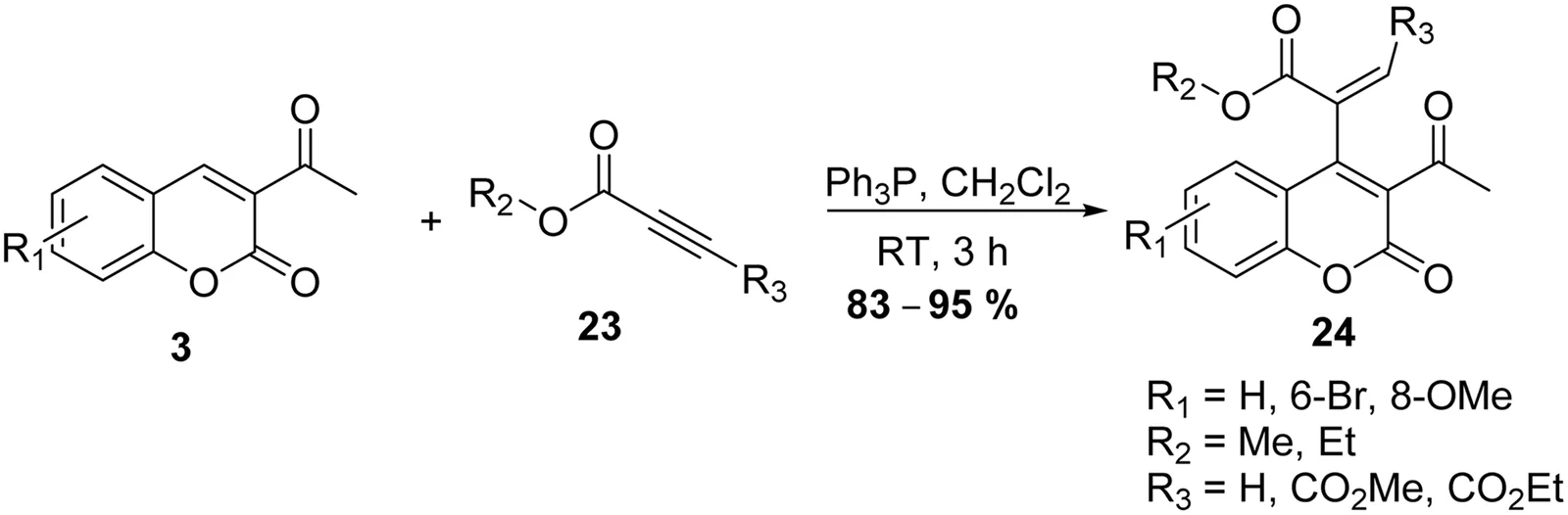
Synthesis of 3-acetyl-4-(substituted ethylenyl)coumarins 24.

Wang *et al.*^179^ described a Ru(ii)-catalyzed C–H alkenylation reactions of 3-acetylcoumarins 3 with acrylates 25 under mild conditions afforded the corresponding 4-alkenylated coumarins 26a–t (Scheme 17).

**Scheme 17:**
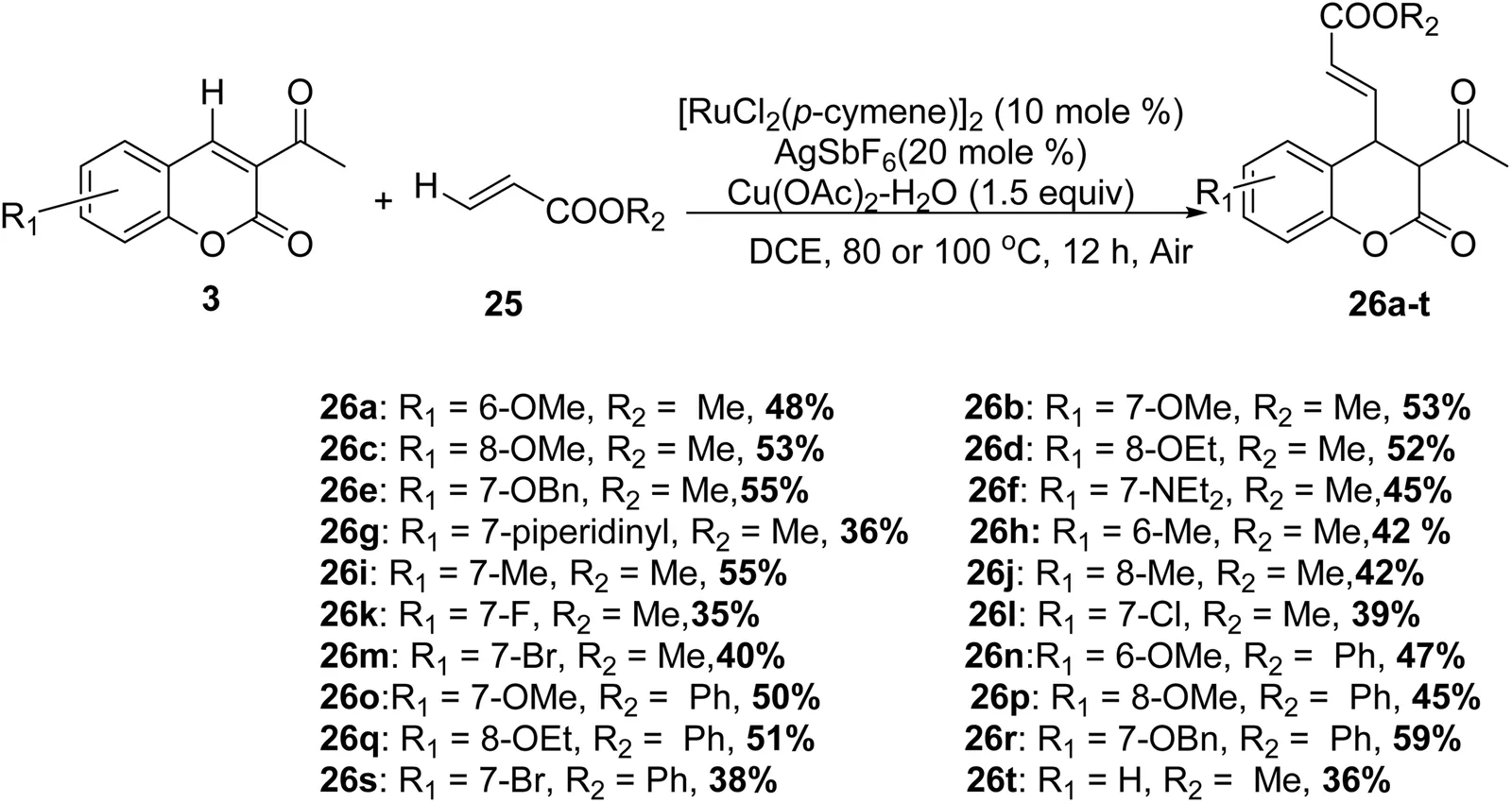
C–H alkenylation reactions of 3-acetylcoumarins 3.

### 4.7. C_4_ cycloalkylation–C_3_ peroxidation

Cycloalkylation–peroxidation of 3-acetylcoumarins 3 has been accomplished *via* multicomponent reactions (MCRs) involving cycloalkanes 27, *tert*-butyl hydroperoxide (TBHP) 28 in the presence of copper(i) oxide^180,181^ or under metal-free conditions, furnished peroxide substrates 29a–g (Scheme 18).^182^

**Scheme 18:**
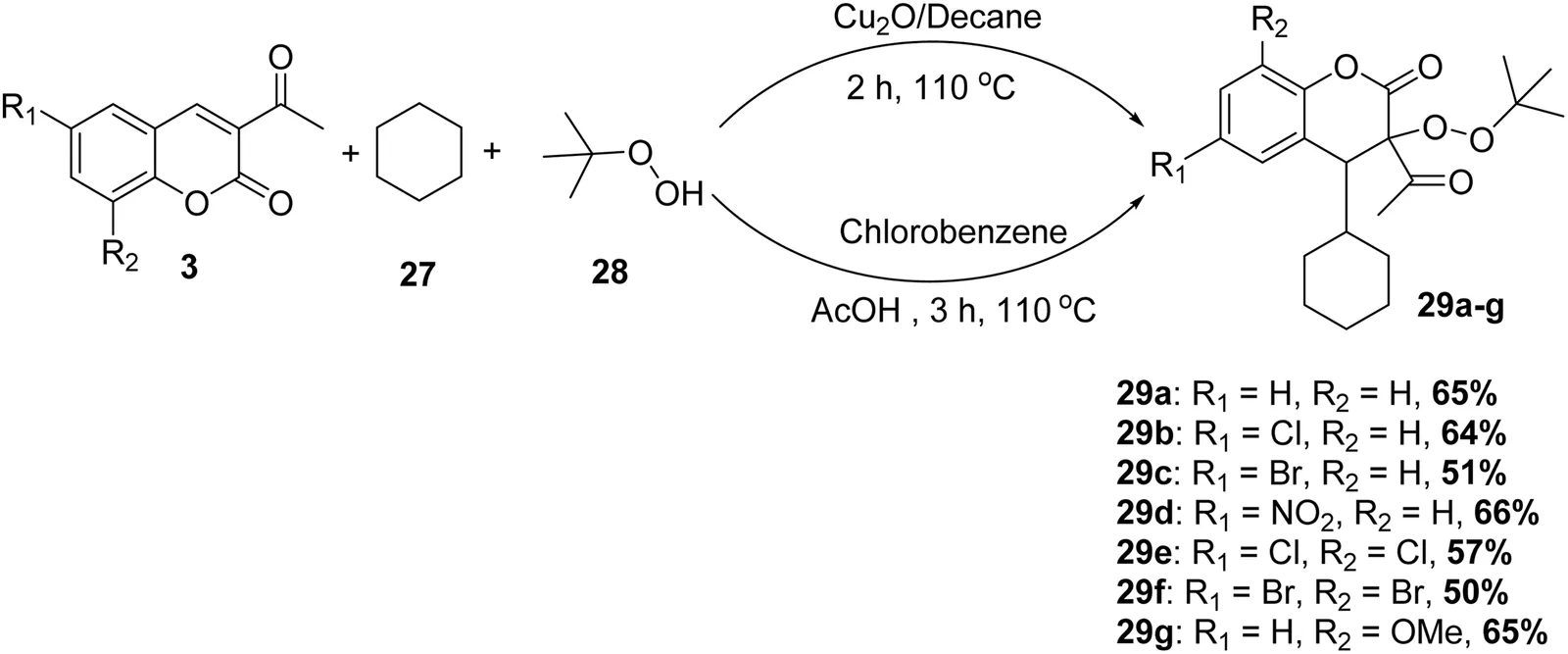
Cycloalkylation peroxidation of 3-acetylcoumarins 3.

### 4.8. Asymmetric Aldol reaction

The asymmetric Aldol reaction between 3-acetylcoumarins 3 and isatin derivatives 30, catalyzed by bifunctional quinidine urea 31, afforded the corresponding 3-hydroxyoxindole derivatives 32 in good yields with high enantioselectivity (Scheme 19).^183^

**Scheme 19:**
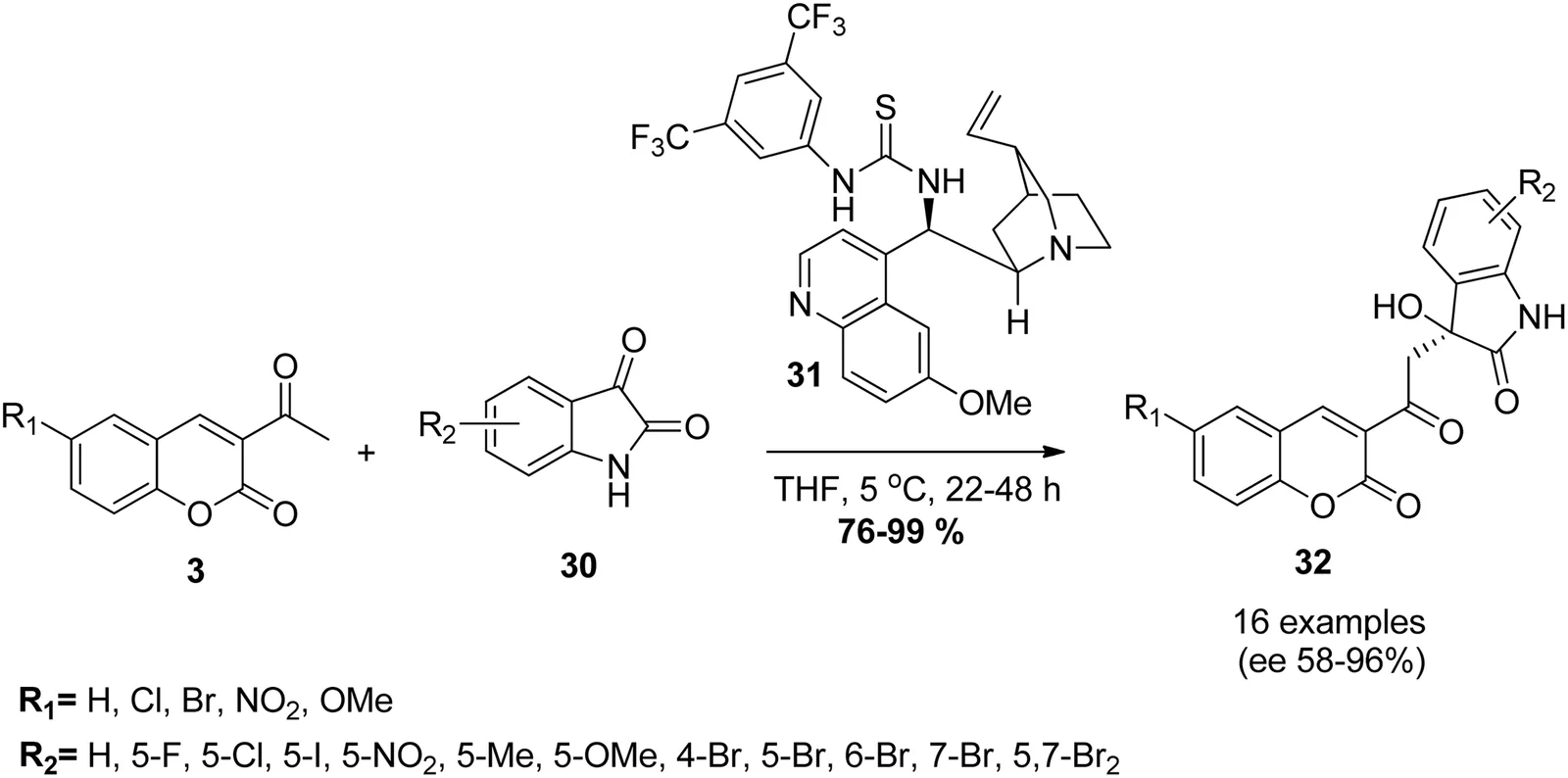
Aldol condensation of 3-acetylcoumarins 3 and isatin derivatives 30.

### 4.9. Cycloaddition reactions

#### 4.9.1. Formal (4 + 2) cycloaddition reactions

Mou *et al.*^153^ reported a rapid new methodology to access benzo[*c*]coumarins 33a–m through base-mediated [4 + 2] of the reaction of unsaturated aldehydes 8 and acetylcoumarin 3 under passing direct air as the green oxidant (Scheme 20).

**Scheme 20:**
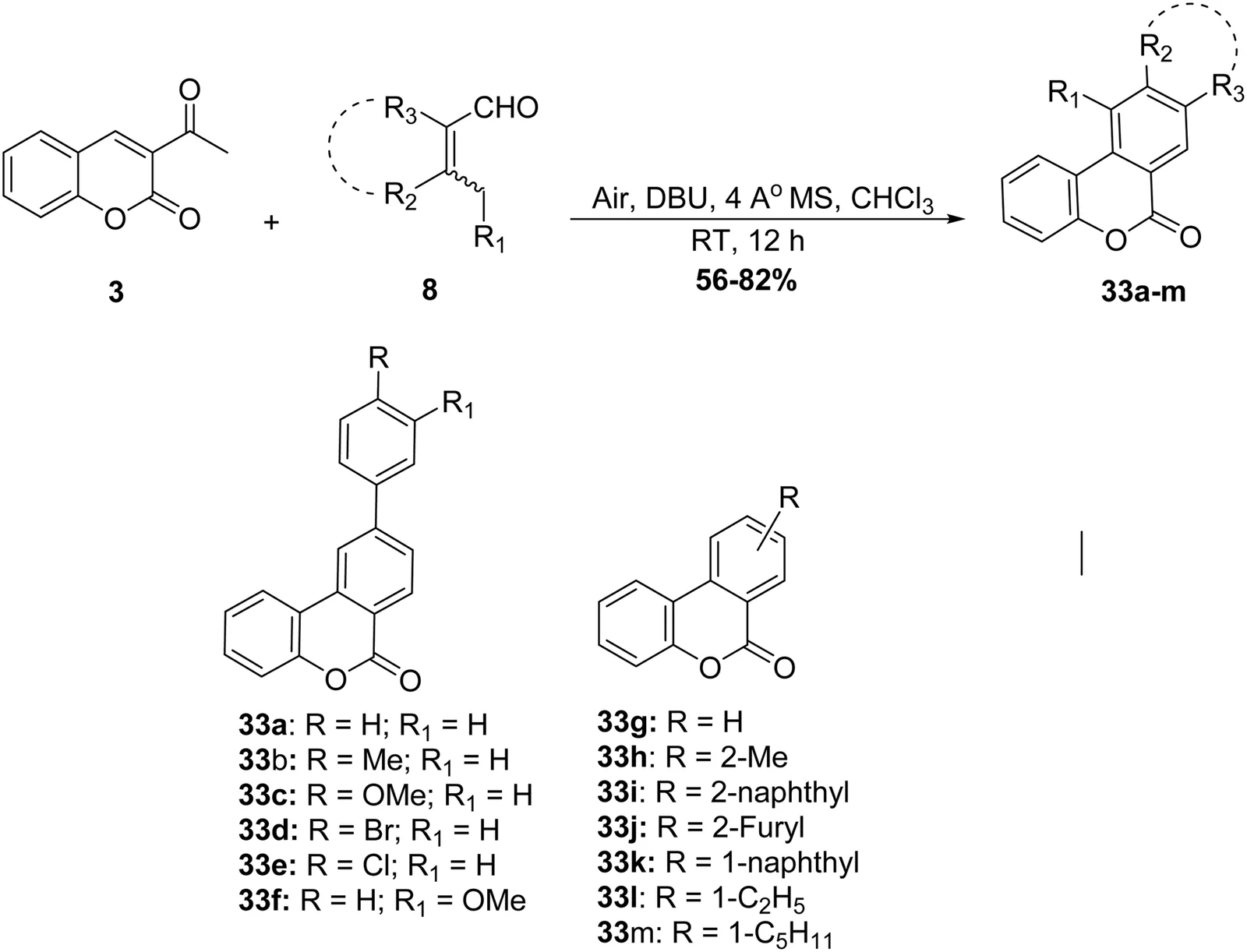
Construction of benzo[*c*]coumarins 33a–m.

#### 4.9.2. Stereoselective cyclopropanation

Zhao *et al.*^184^ developed a catalyst-free cyclopropanation reaction of 3-acylcoumarin 3 with α-bromoketones 34 under mild room-temperature conditions using NaOH as the base. This protocol provided coumarin-based cyclopropane derivatives 35 in moderate yields with excellent *cis*-selectivity (Scheme 21).

**Scheme 21:**
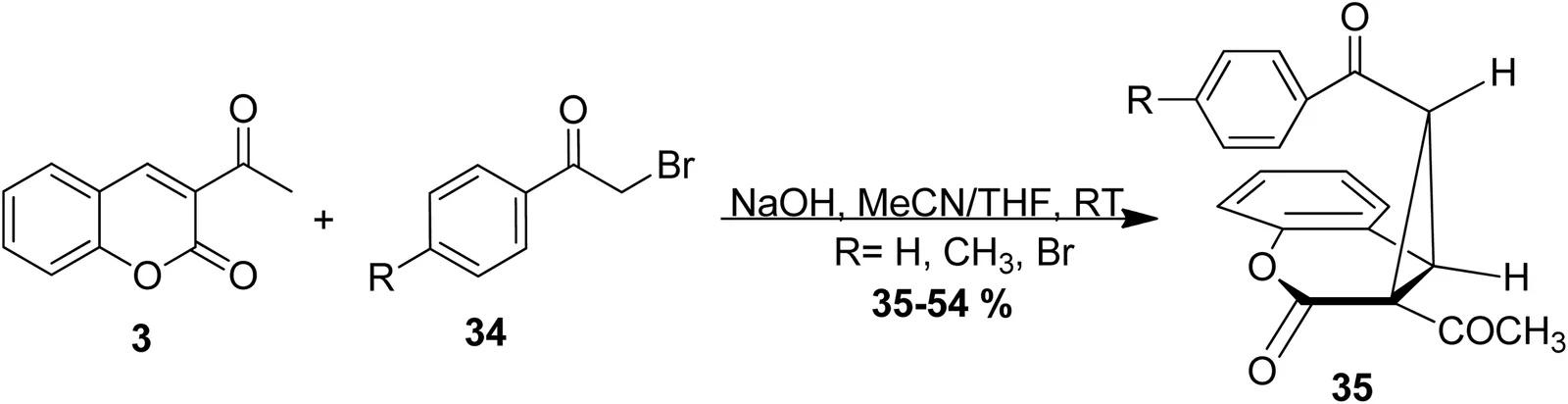
Cycloaddition reaction of 3 with phenacyl bromide 34.

#### 4.9.3. Cross‐dehydrogenative coupling

Copper(ii) mediated cross-dehydrogenative coupling of 8-methylquinoline 36 with 3-acetylcoumarin 3 to afford the corresponding (*E*)-(quinolin-8-yl)enone 37 in pivalic acid (PivOH) (Scheme 22).^185^

**Scheme 22:**
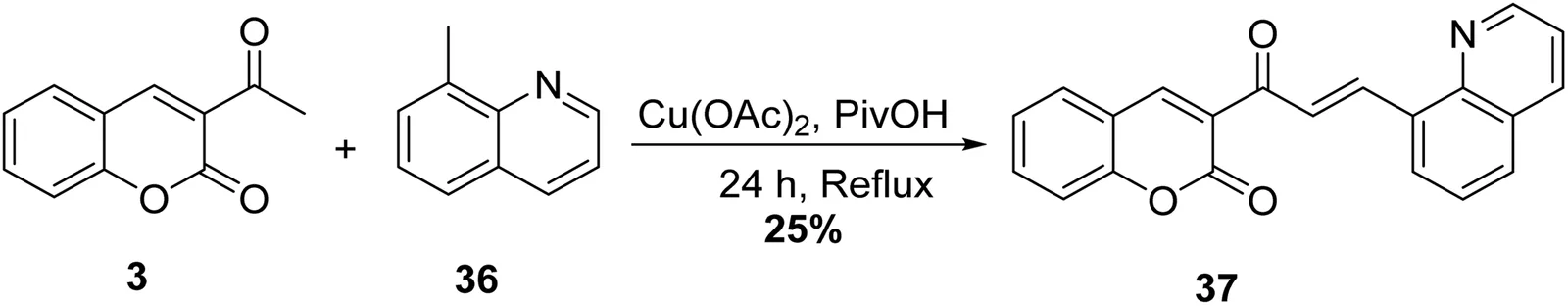
Treatment of acetylcoumarin 3 with 8-methylquinoline 36.

### 4.10. Reaction with active methylene components

3-Acetylcoumarin derivatives 3 were reacted with 1,3-diphenylpropane-1,3-dione 38 in DMSO in the presence of an iodine/cupric oxide catalytic system, leading to the formation of the corresponding 1,4-enedione derivatives 39a–f (Scheme 23).^186^

**Scheme 23:**
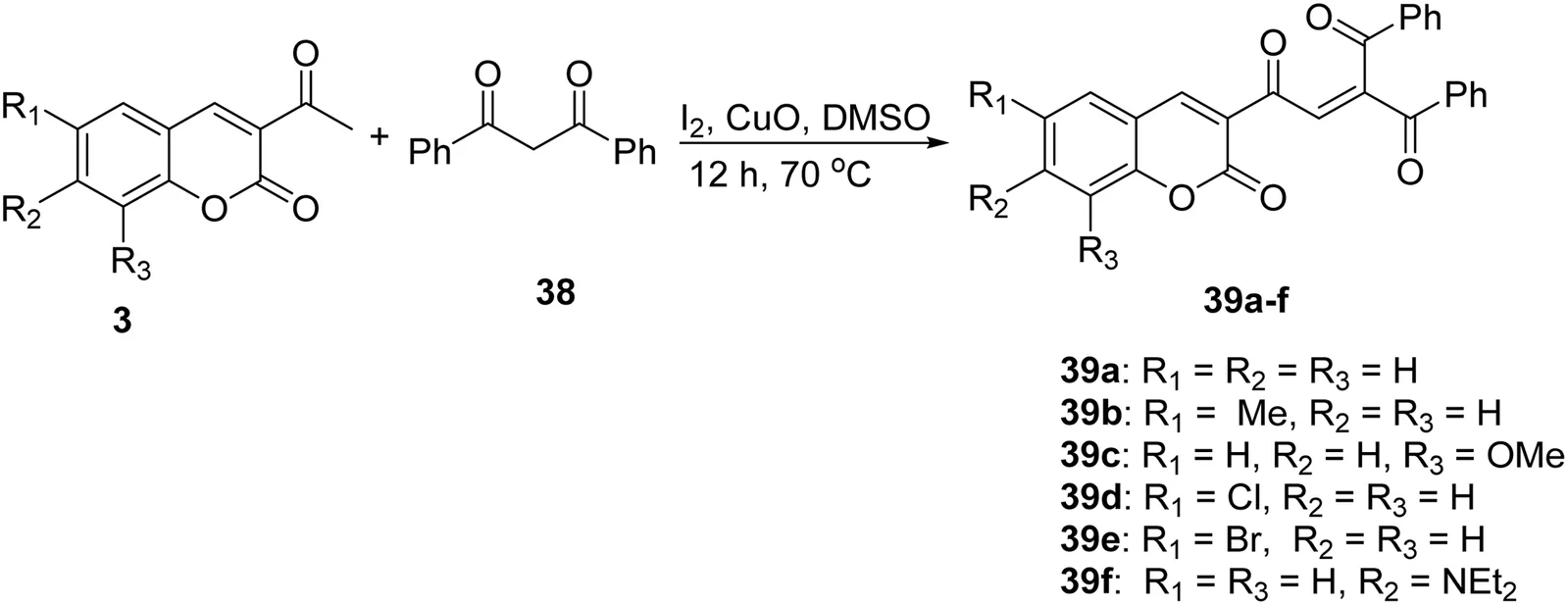
Condensation of 3 with 1,3-diphenylpropane-1,3-dione 38.

3-(2,2-Dicyano-1-methylvinyl)coumarins 42a–j were synthesized through the condensation reaction of 3, malononitrile 40, and ammonium acetate 41 in benzene, acetic acid, solvent-free condition, or diethylamine (DEA)^187–189^ were completed efficiently with good to excellent yield at ambient temperature (Scheme 24).

**Scheme 24:**
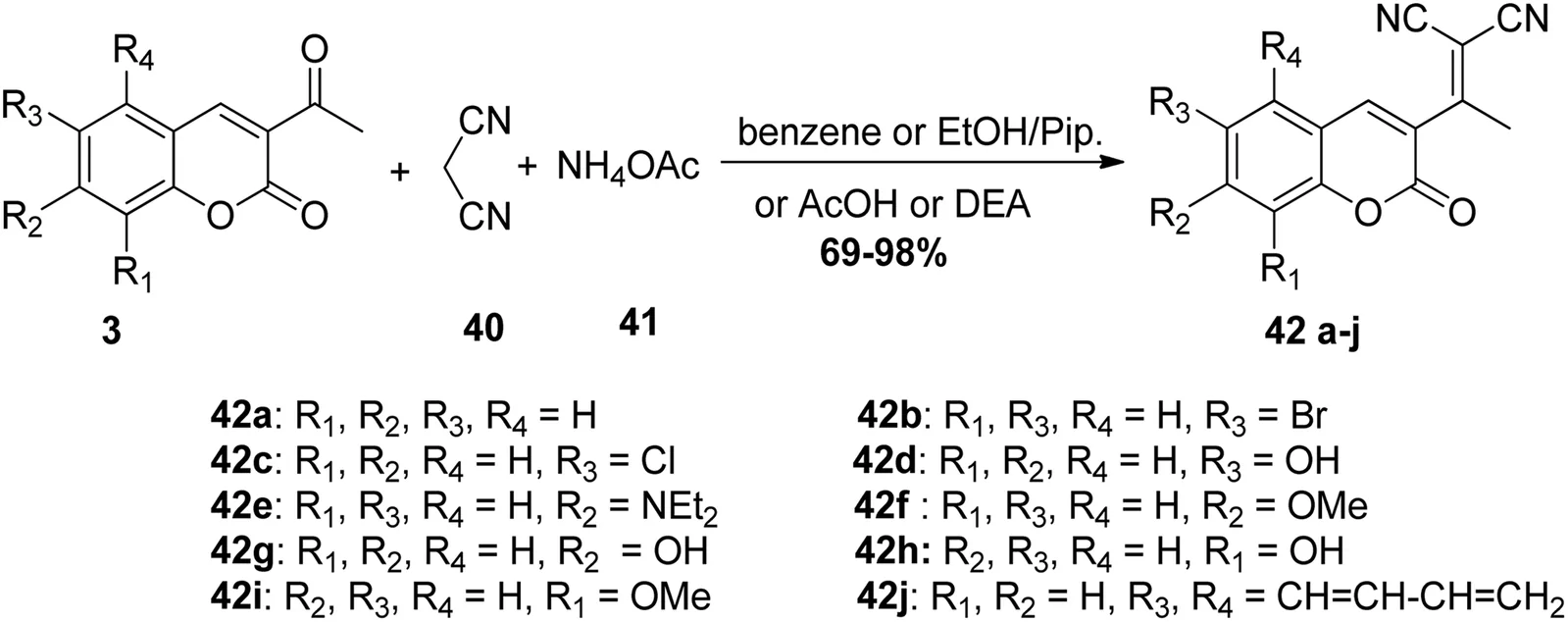
One-pot reaction of 3, malononitrile 40, and ammonium acetate 41.

### 4.11. Halogenation

#### 4.11.1. Iodination reaction

Selective and efficient aerobic oxidative α-iodination of 3-acetylcoumarin 3 using a catalytic system containing ammonium nitrate (NH_4_NO_3_) and drops of conc. H_2_SO_4_ to afford 3-(2-iodoacetyl)-2*H*-chromen-2-one 43 (Scheme 25).^190^

**Scheme 25:**
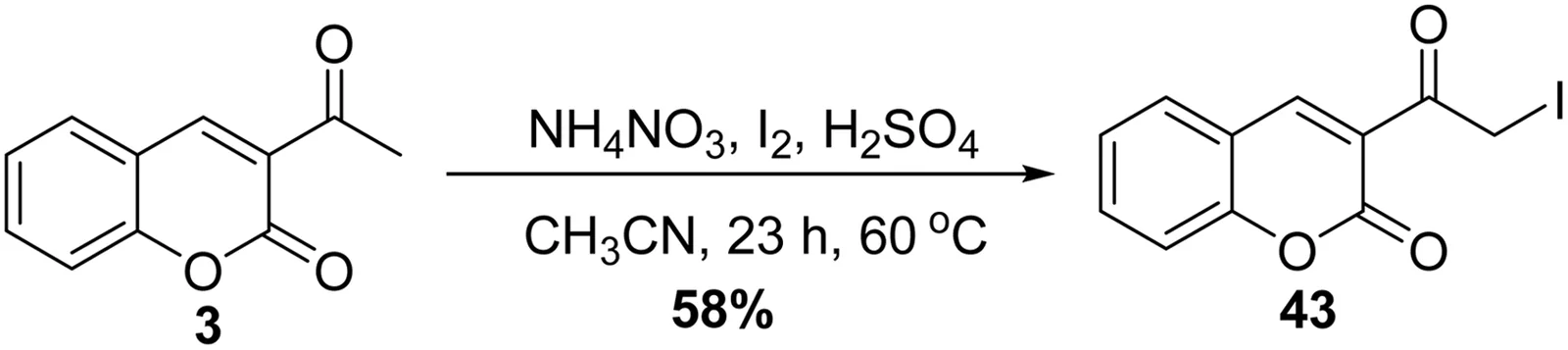
Iodination of 3-acetylcoumarin 3.

#### 4.11.2. Chlorination reaction

α-Chlorination of 3 under acidic aerobic oxidative conditions, using catalytic I_2_ and ammonium nitrate as an inexpensive source of NO/NO_2_, produced the corresponding chlorinated derivative 44 (Scheme 26).^191^

**Scheme 26:**
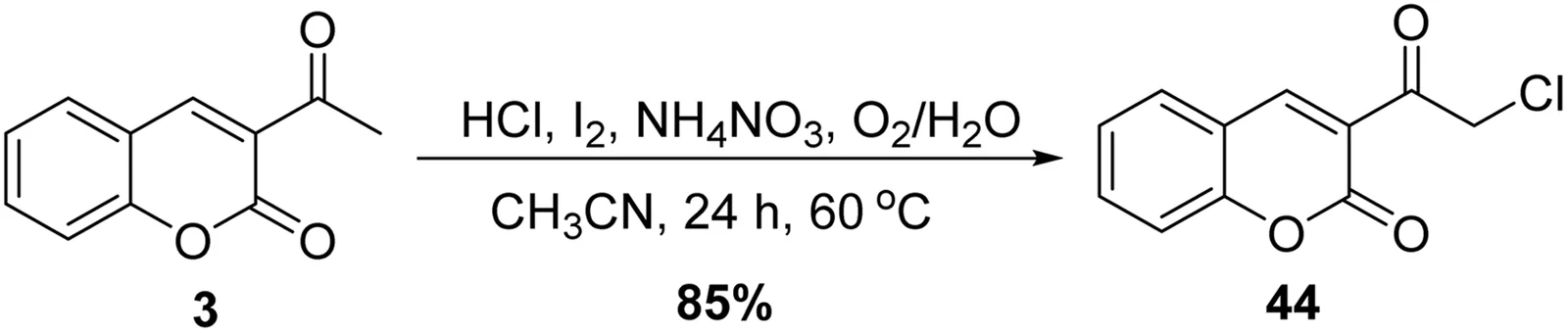
Chlorination of 3-acetylcoumarin 3.

#### 4.11.3. Bromination reaction

The synthesis of 3-bromoacetylcoumarin derivatives 45a–o through the reaction of 3-acetylcoumarins 3 with various brominating agents like tetrabutylammonium tribromide (TBATB), bromine in chloroform or acetic acid, CuBr_2_, phenyltrimethylammonium tribromide, tetra-*n*-butylammonium tribromide (TBABr_3_) have been reported (Scheme 27).^147,192–208^

**Scheme 27:**
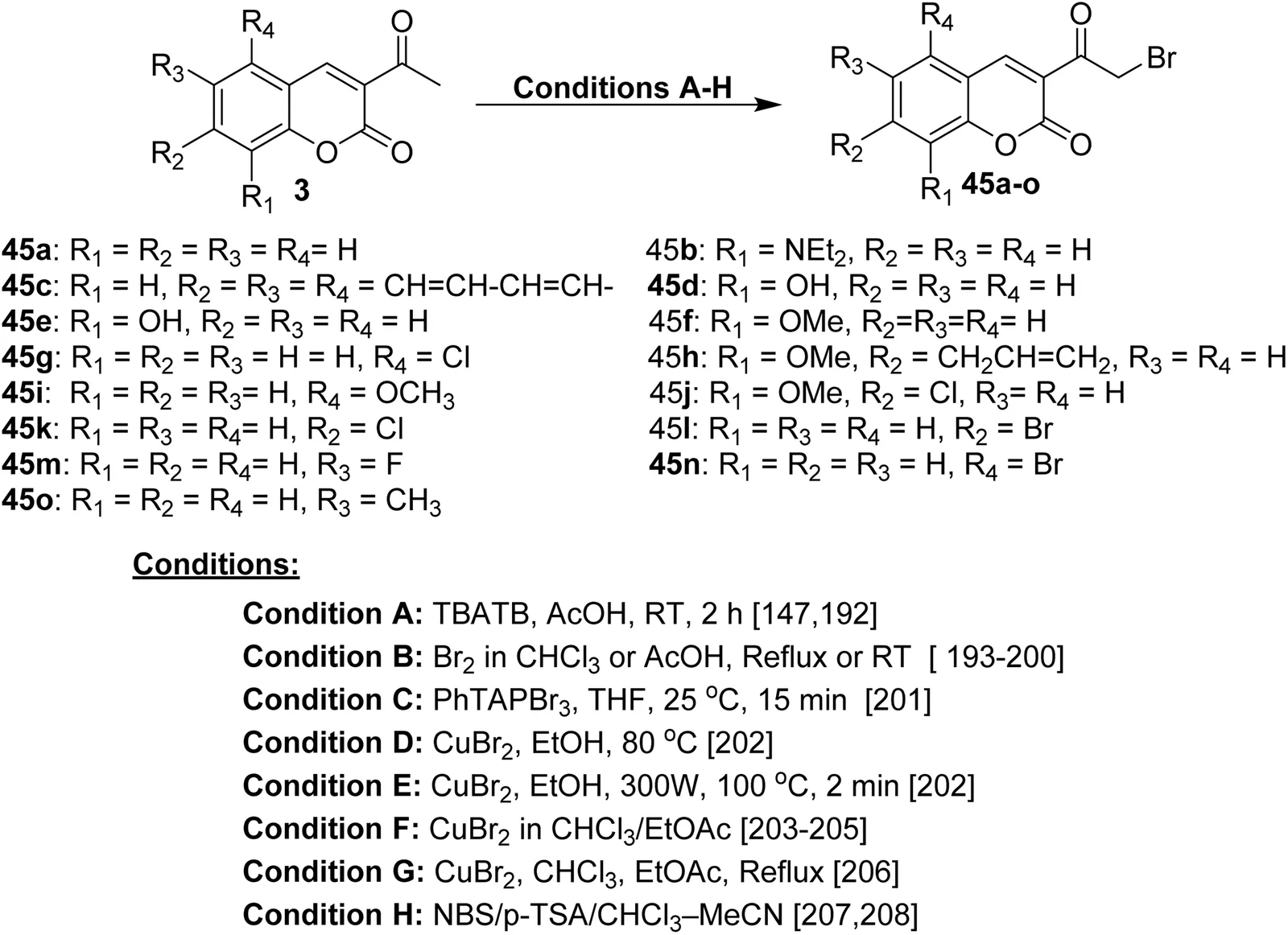
Bromination of 3-acetylcoumarins 3.

### 4.12. Reduction reaction

The synthesis of the dihydro dimer 3,3′-diacetyl-4,4′-bichroman-2,2′-dione 46 from 3-acetylcoumarin 3 was achieved *via* ultrasound-assisted metal-mediated homodimerization (Scheme 28).^206^

**Scheme 28:**
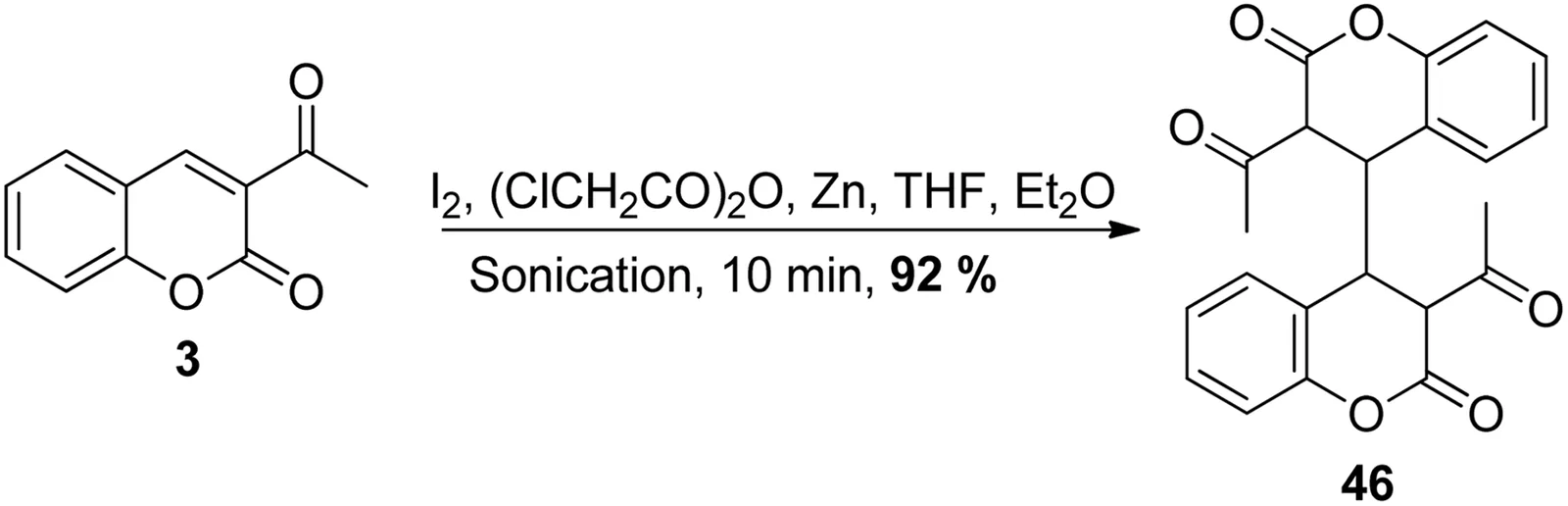
Dimerization of 3-acetylcoumarin 3.

In contrast, selective hydrogenation of the endocyclic double bond in 3-acetylcoumarin 3 using Pd/C in methanol afforded 3,4-dihydrocoumarin 47 (Scheme 29).^209^

**Scheme 29:**
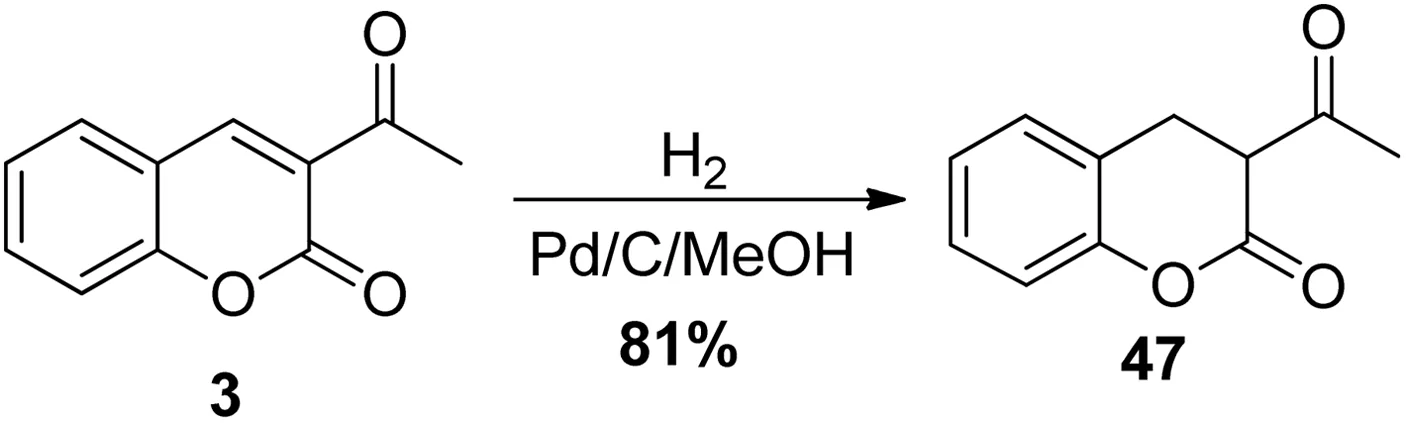
Pd/C-catalyzed reduction of 3 to 3,4-dihydrocoumarin derivative 47.

### 4.13. Thionation and sulfur-functionalisation reaction

The one-pot reaction of 3 and different heterylthiols 48 was performed independently in polyethylene glycol (PEG-600) *via* using tetrabutylammonium tribromide (TBATB) as an effective reagent to afford the corresponding products 49a–c.^210^ The transformation also proceeded under iodine catalysis in isopropyl alcohol at room temperature (Scheme 30).^211^

**Scheme 30:**
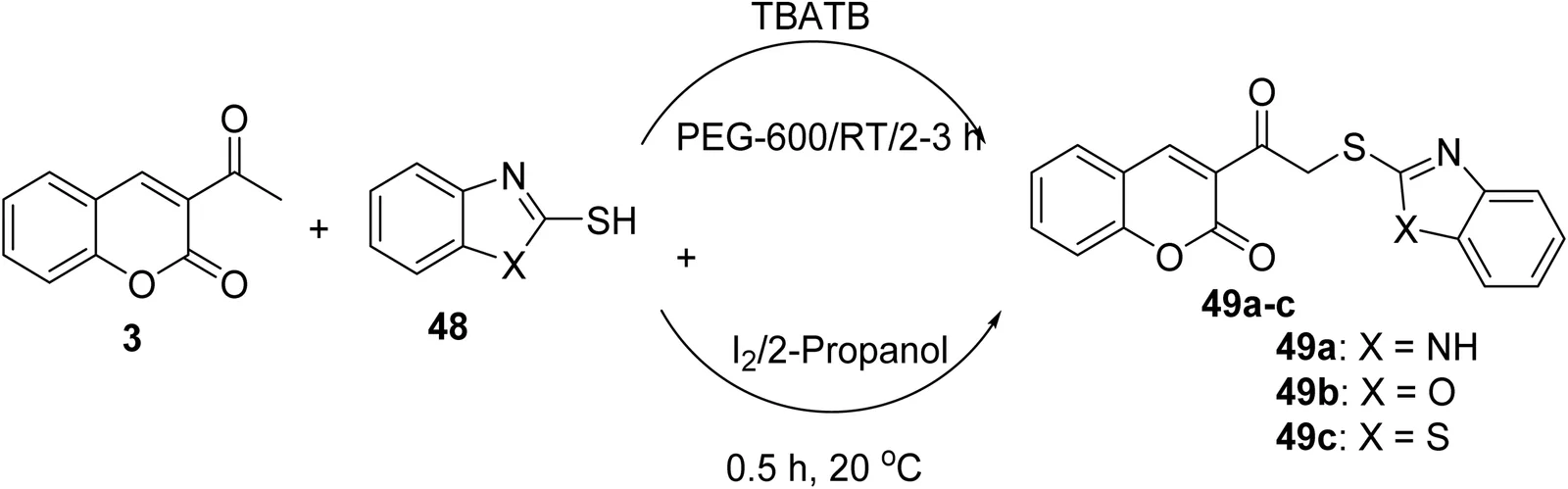
Thionation of 3-acetylcoumarin 3.

Polyethylene glycol (PEG-600) mediated one-pot reaction between 3 and axanthate salts 50 without using of phase transfer catalyst (PTC) or any base to obtain the series of thioesters 51a–c (Scheme 31).^147^

**Scheme 31:**
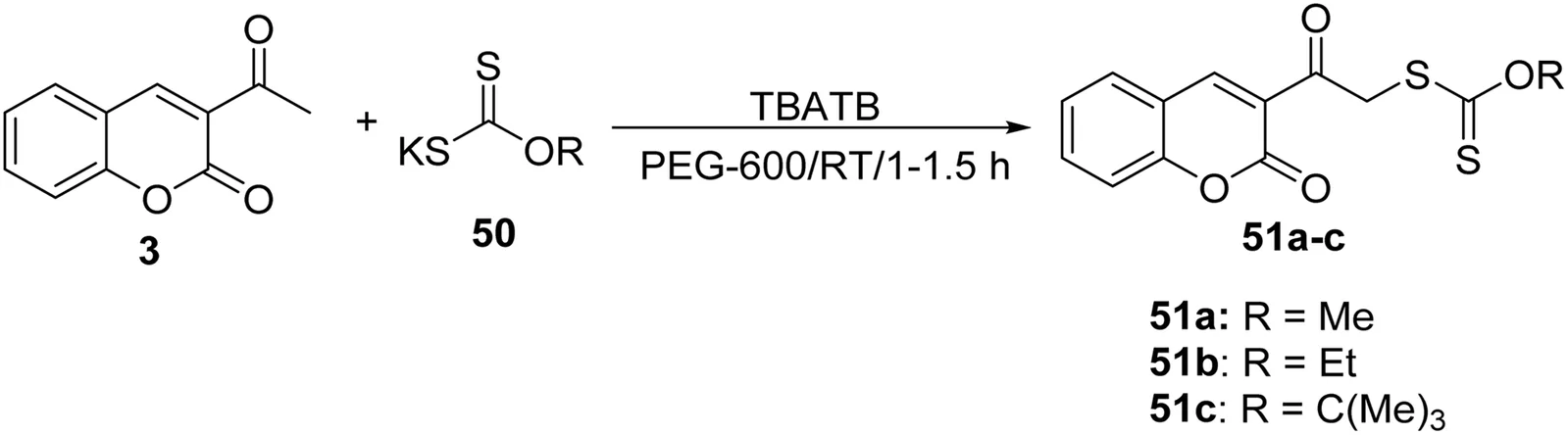
One-pot reaction of 3-acetylcoumarin 3, TBATB, and xanthate salts 50.

Treatment of coumarin derivative 3 with *N*-trifluoromethylthiosulfon amide 52 as acid-catalyzed trifluoromethylthiolation agent afforded 3-(2-((trifluoromethyl)sulfanyl)acetyl)-2*H*-chromen-2-one 53 (Scheme 32).^212^

**Scheme 32:**
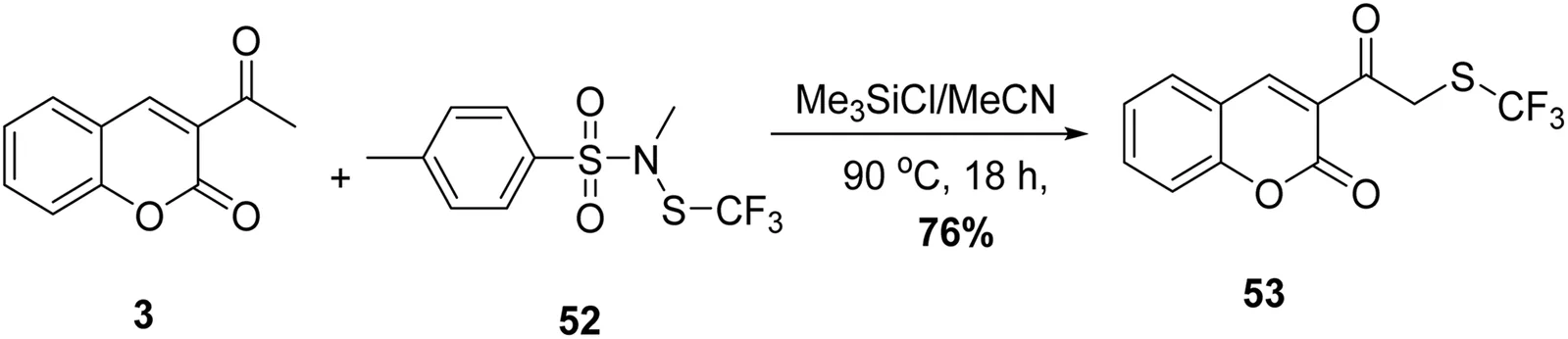
Preparation of 3-(2-((trifluoromethyl)sulfanyl)acetyl)-2*H*-chromenone 53.

### 4.14. Phosphorylation reaction

*N*-Substituted (3-(1-aminoethylidene)-2-oxochromon-4-yl)phosphonic acid diethyl ester derivatives 56a–y were synthesized *via* a one-pot reaction of 3-acetylcoumarin 3, substituted amines 54, and diethyl phosphite 55 under neat ultrasonication radiation conditions (Scheme 33).^213^

**Scheme 33:**
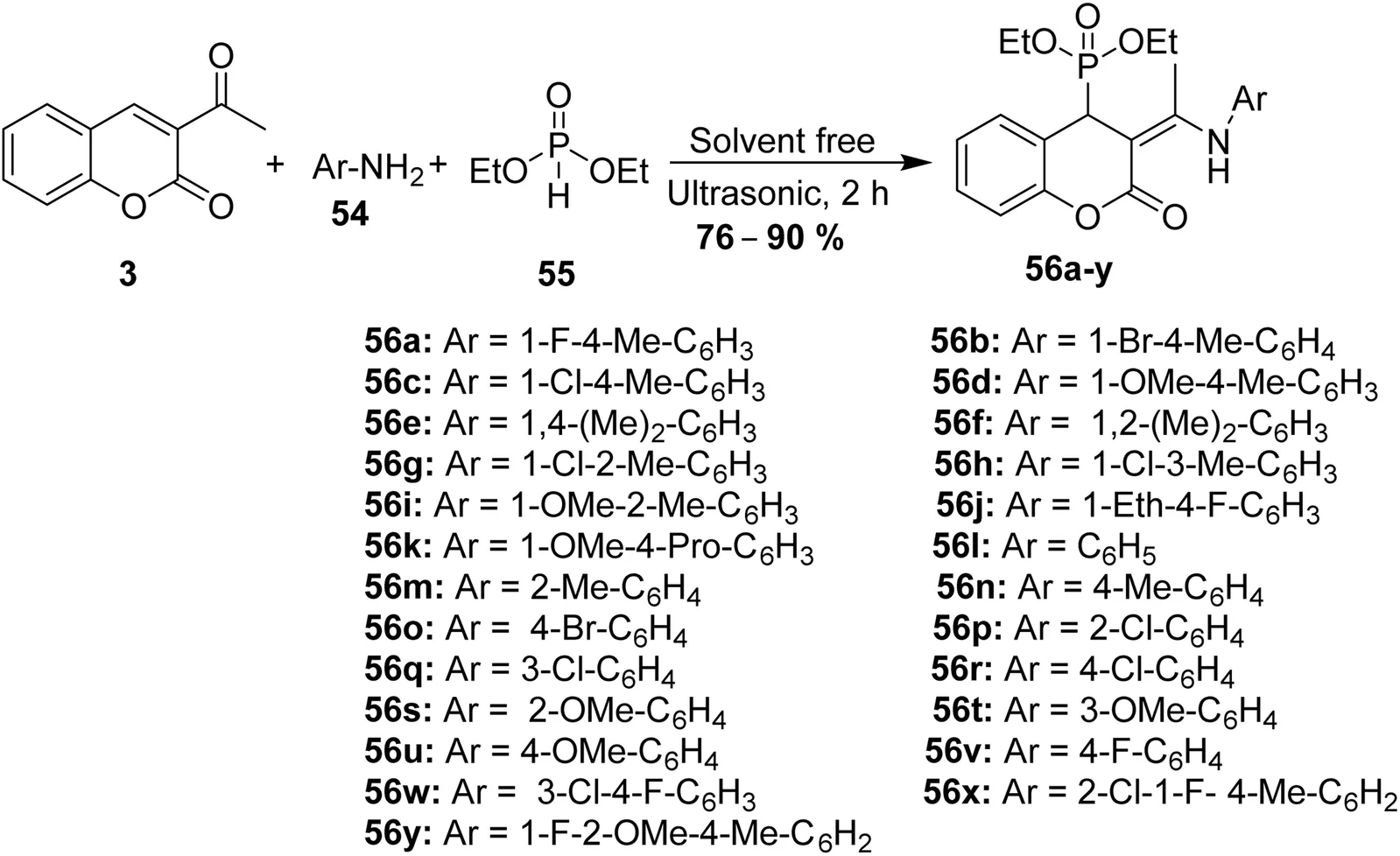
Phosphorylation of 3-acetylcoumarin 3.

Silver-catalyzed highly regioselective phosphorylation Csp^2^–H of coumarin 3 with trialkyl phosphites 55 a reaction of coumarin 3 with phosphate derivatives 55 in the presence AgNO_3_/Mg(NO_3_)_2_·6H_2_O as catalyst^214^ or *N*-heterocyclic carbene palladium complex (id)^215^ to afford selective coumarin-3-yl phosphonate 57 (Scheme 34).

**Scheme 34:**
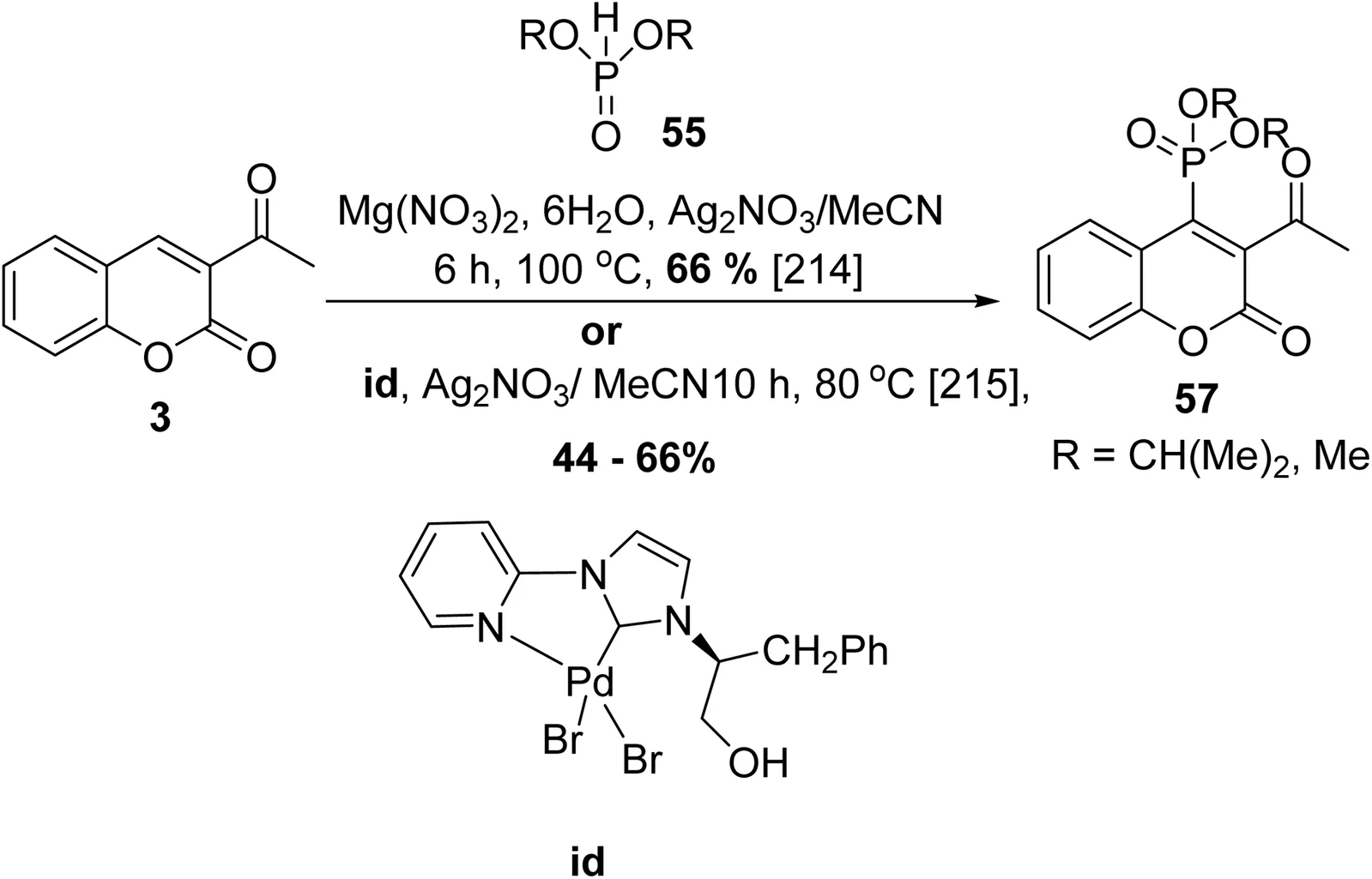
Preparation of phosphated based-coumarin scaffolds 57.

### 4.15. Reactions with nitrogen nucleophiles

#### 4.15.1. Reaction with amines

Both microwave-assisted^216^ and conventional synthesis^217,218^ have been employed as green and traditional approaches, respectively, for the synthesis of Schiff bases 58*via* the reaction of 3 with various amines 54 in ethanol. Additionally, a Schiff base derivative 60 was obtained through the condensation of 3-acetylcoumarin with diethylenetriamine 59 in a 2 : 1 molar ratio (Scheme 35).^218–228^

**Scheme 35:**
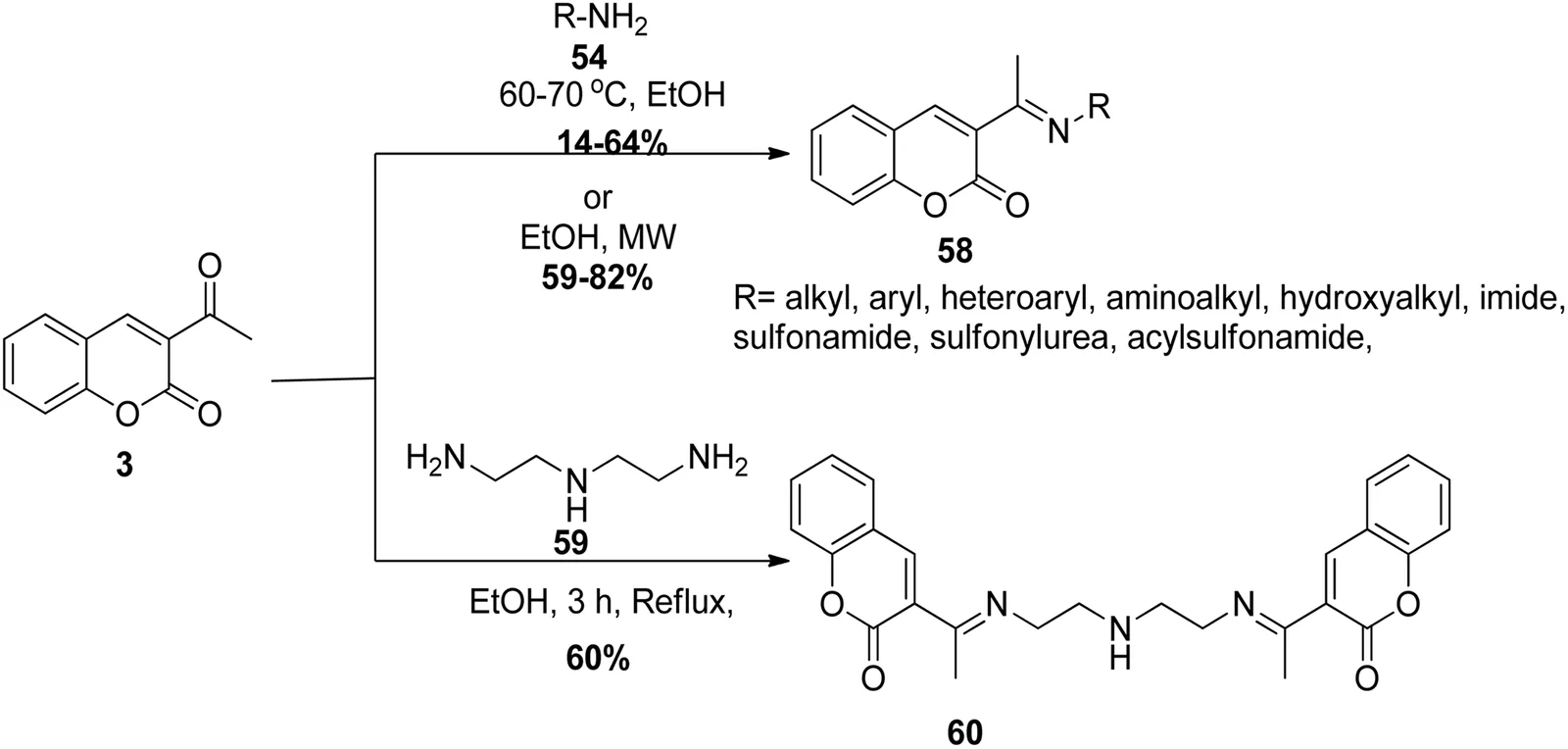
Treatment of 3-acetylcoumarin 3 with various amines 54.

#### 4.15.2. Reaction with hydroxylamines

Refluxing 3-acetylcoumarin 3 with an excess of hydroxylamine hydrochloride 61 in ethanol containing a catalytic amount of sodium acetate afforded oxime 62.^229,230^ On the contrary, the same reaction in the presence of thiamine hydrochloride through Beckmann rearrangement affording 3-acetamidocoumarin 63 in excellent yield (Scheme 36).^231^

**Scheme 36:**
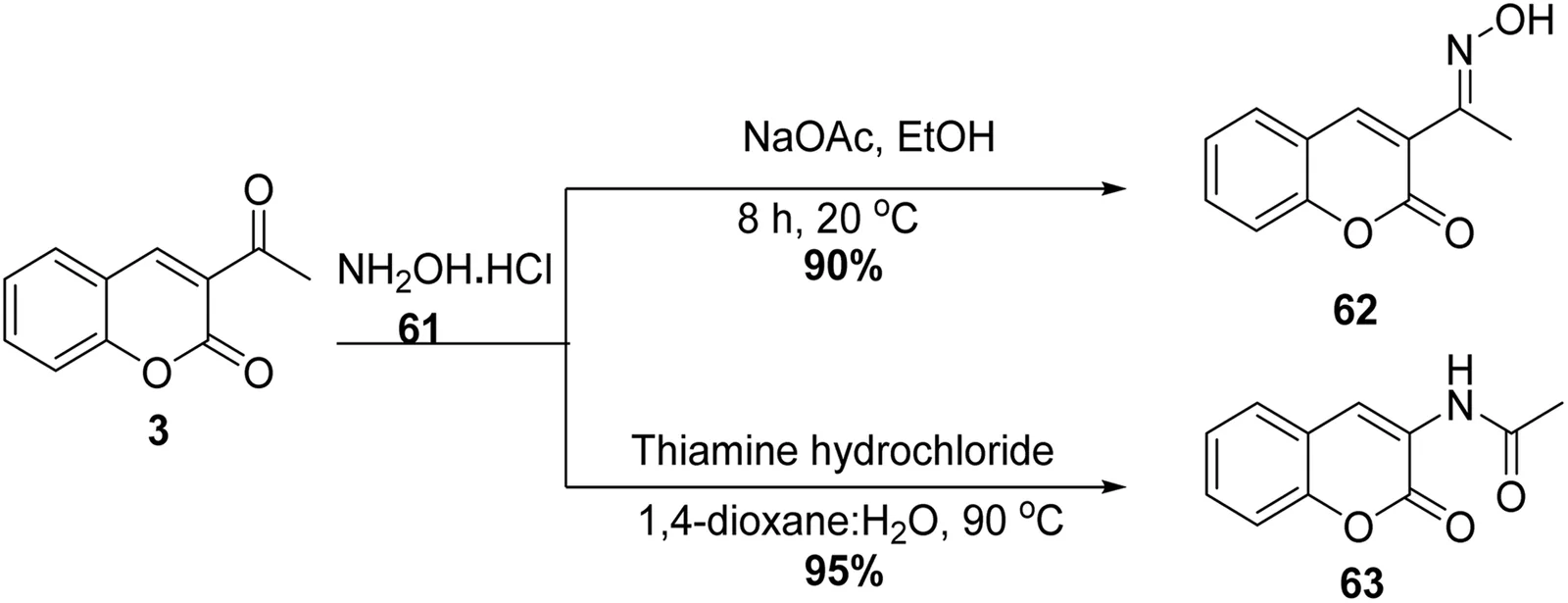
Reaction of 3 with hydroxylamine derivatives 61.

Coumarin-oxime ether derivatives 65 were prepared through a simple and efficient reflux procedure involving 3-acetylcoumarin 3 and *o*-substituted benzylhydroxylamines 64 in pyridinium *p*-toluenesulfonate/dichloromethane (PPTS/DCM) (Scheme 37).^232,233^

**Scheme 37:**
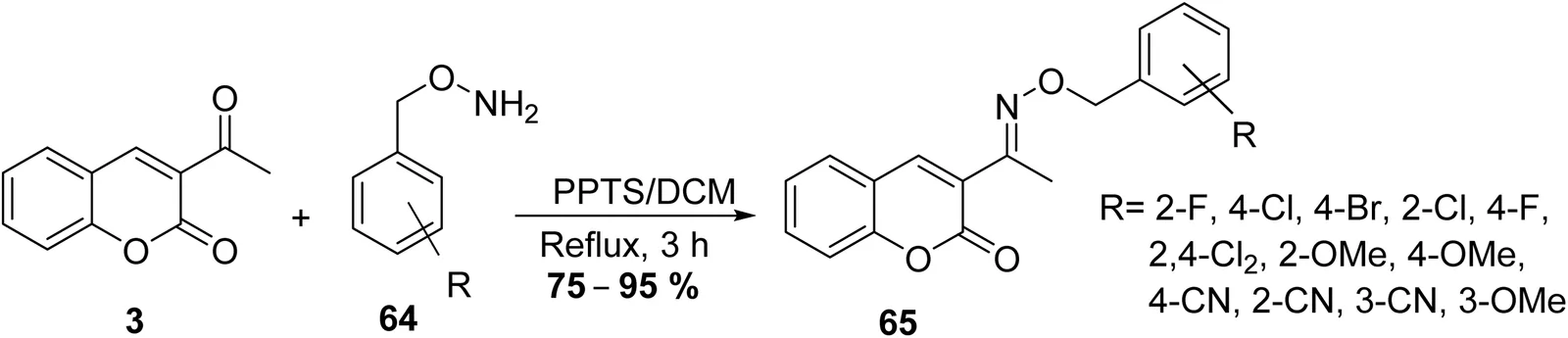
Formation of coumarin-oxime ethers 65.

#### 4.15.3. Mannich-type reactions

The Mannich base 66 of 3-acetylcoumarin was synthesized by condensing acetylcoumarin 3 with paraformaldehyde 8 and dimethylamine 54 in the presence of concentrated hydrochloric acid (Scheme 38).^234,235^

**Scheme 38:**
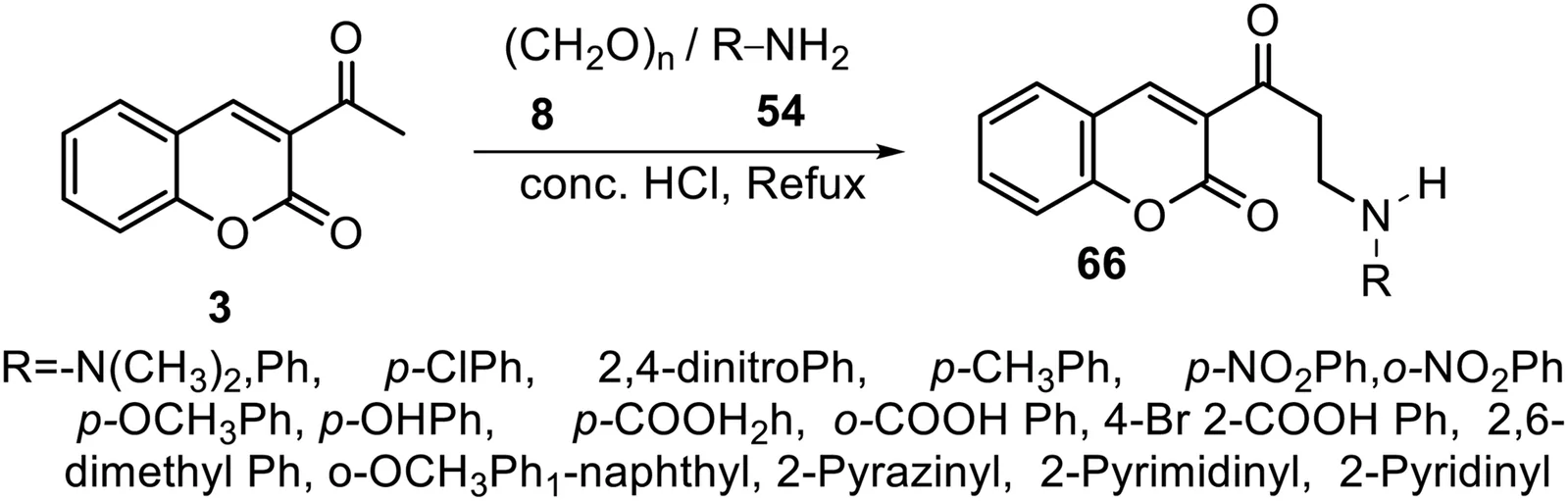
Synthesis of Mannich base 66.

Mannich type four-component reaction of 3-acetylcoumarin 3 with aldehyde derivatives 8, acetyl chloride 67 and bromo-acetonitrile 68 in the presence of various catalysts such as phenylboronic acid, copper(ii) sulfate,^236^ or boron trifluoride diethyl etherate^237^ yielded different coumarin alkyl amides 69 (Scheme 39).

**Scheme 39:**
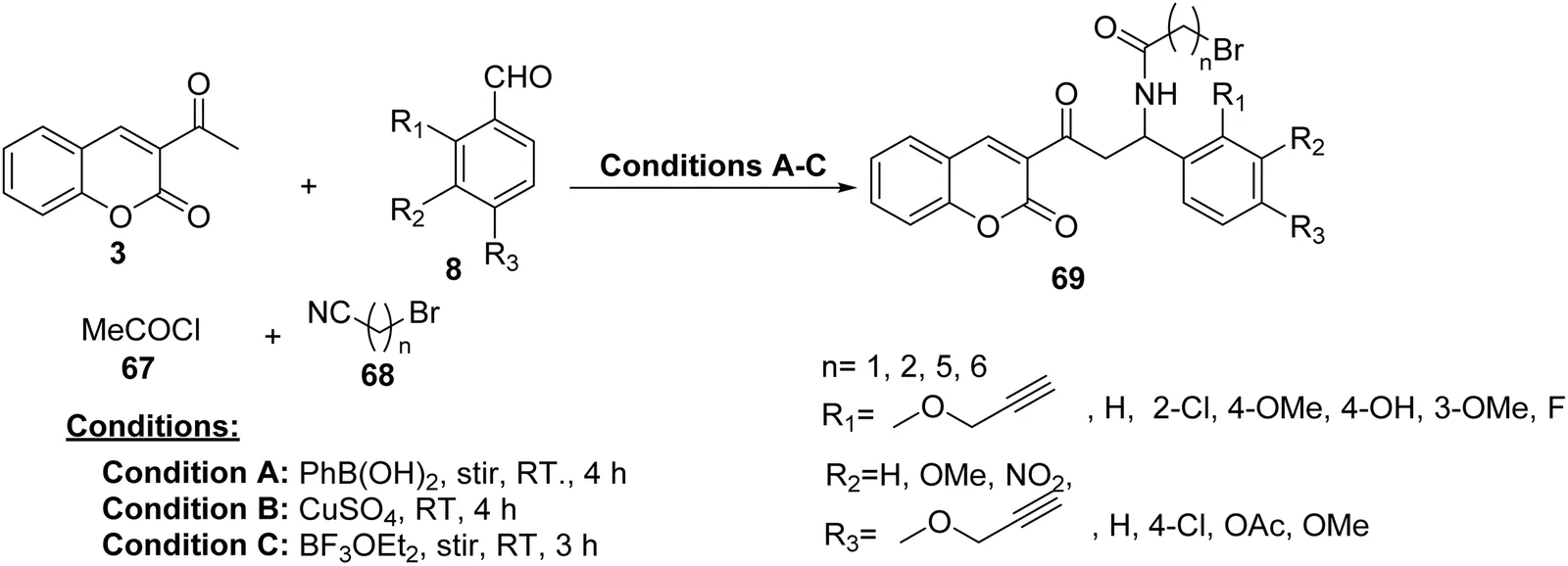
Four-component Mannich reaction to afford coumarin alkyl amides 69.

#### 4.15.4. Formation of enaminones

Enaminone 71 was synthesized from 3-acetylcoumarin 3 and DMF-DMA 70 under various conditions: l-proline (80 °C, 1.5 h, 92% yield), guanidine acetic acid (100 °C, 1.5 h, 85%), reflux in 1,4-dioxane, and xylene (6 h, 75%), with l-proline catalysis being the most efficient (Scheme 40).^238–243^

**Scheme 40:**
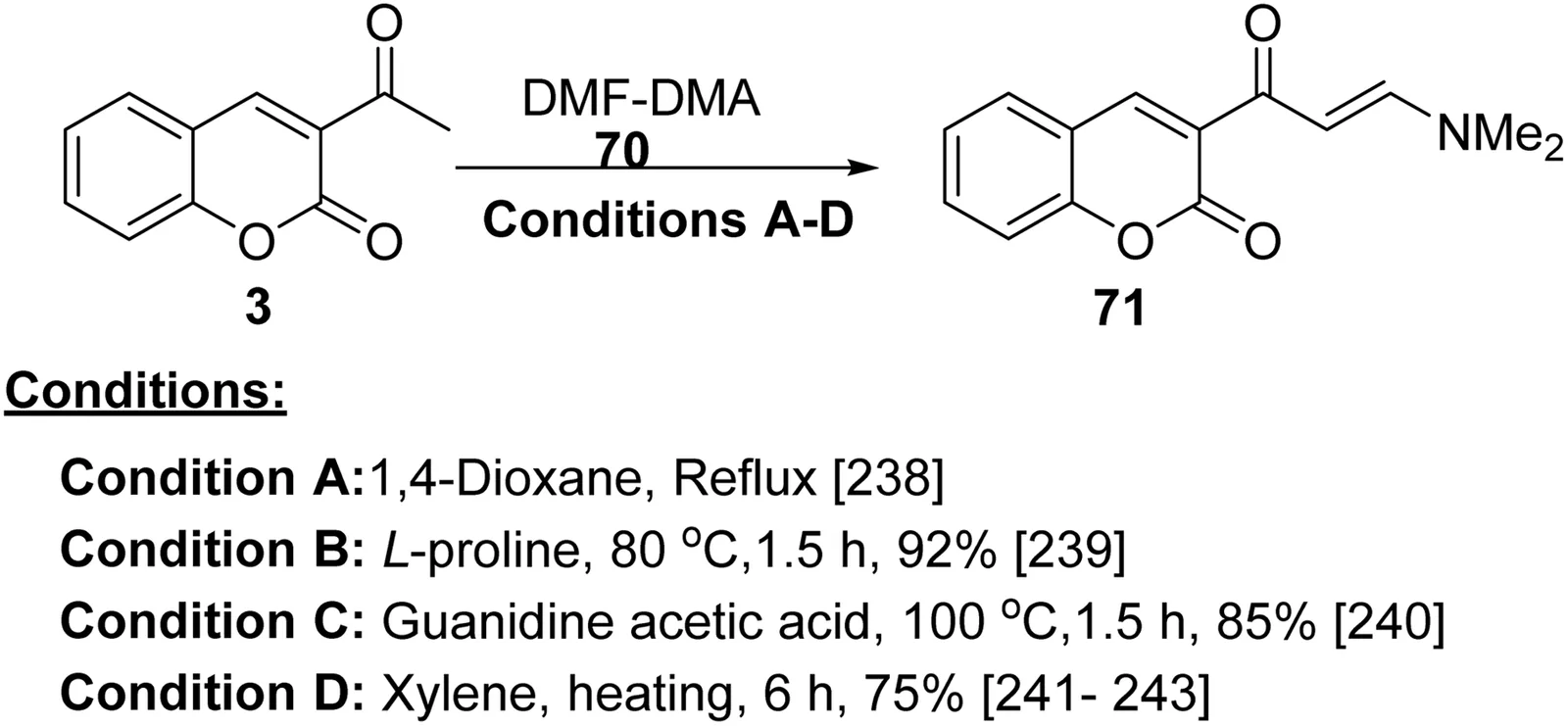
Synthesis of enaminone 71 from coumarin 3.

#### 4.15.5. Condensation with hydrazine and hydrazine derivatives

Substituted 3-acetylcoumarins 3 were converted into 3-acetyl-1-aminoquinolin-2-one derivatives 73 by refluxing them with hydrazine hydrate 72. The reaction was carried out in different solvents and could be promoted using catalysts such as sodium acetate, hydrochloric acid, or acetic acid (Scheme 41).^244,245^

**Scheme 41:**
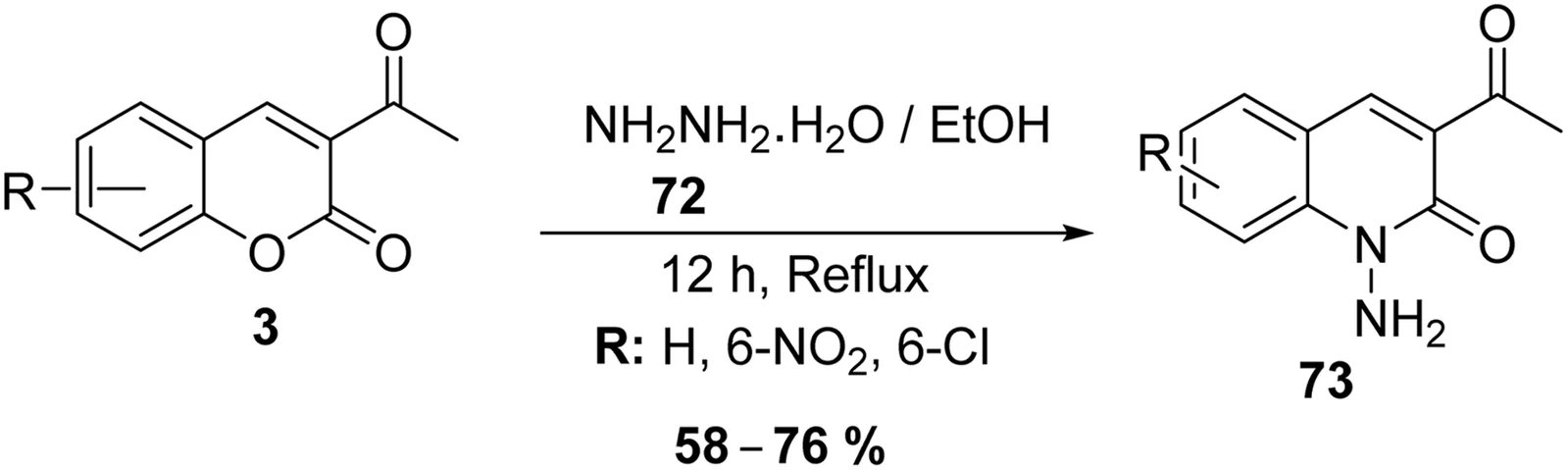
Treatment of 3-acetylcoumarin 3 with hydrazine hydrate 72.

Meanwhile, 3-acetylcoumarin 3 was treated with hydrazines 74 to afford the corresponding Schiff bases 75 under conventional heating or microwave irradiation in different solvents and in the presence of catalysts such as sodium acetate, hydrochloric acid, or acetic acid (Scheme 42).^168,246–282^

**Scheme 42:**
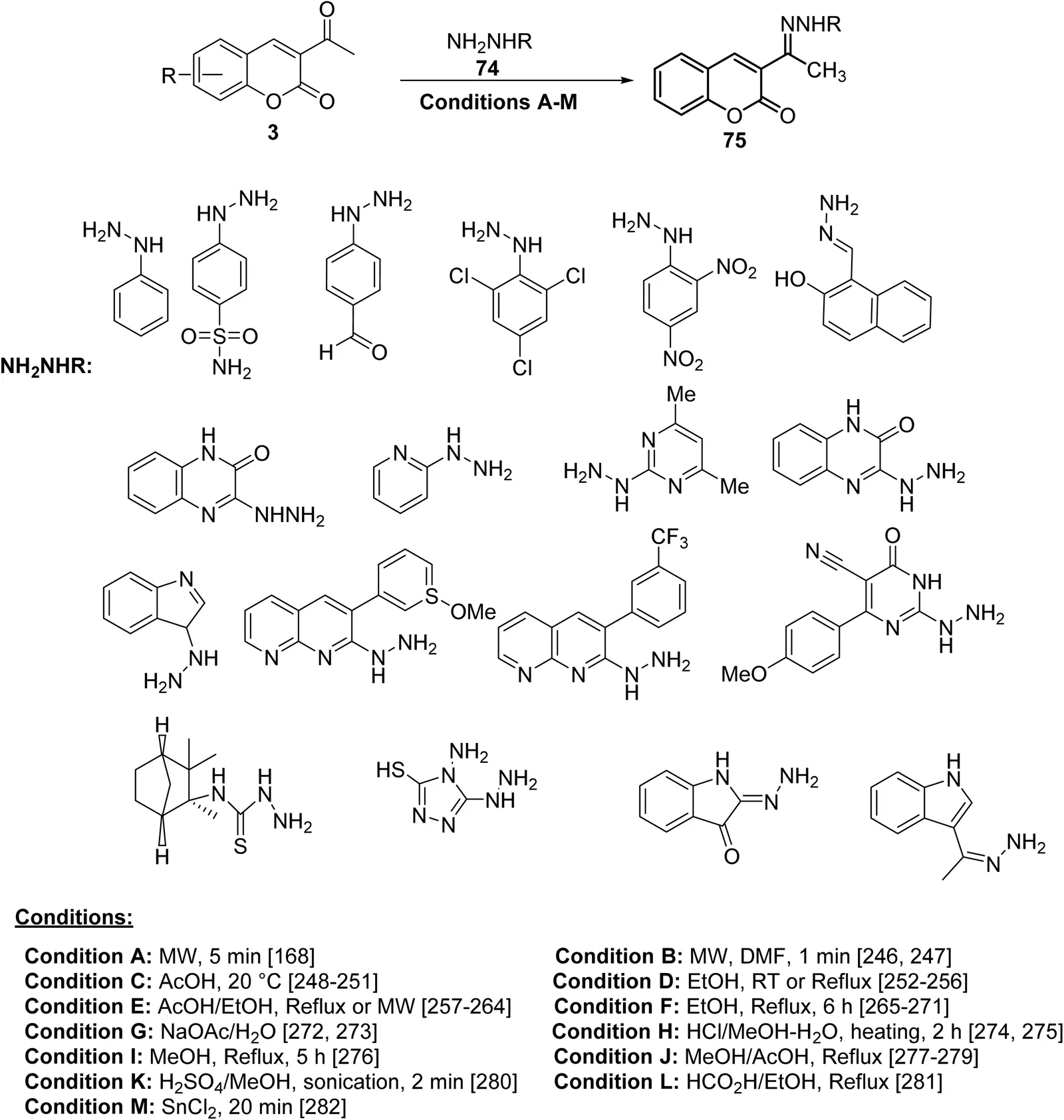
Treatment of 3-acetylcoumarin 3 with hydrazines.

Refluxing 3-acetylcoumarin 3 with isothiocyanates 76 and hydrazine hydrate 72 in ethanol containing drops of glacial acetic acid as a catalyst gave the target products 77 in 84–94% yield (Scheme 43).^283^

**Scheme 43:**
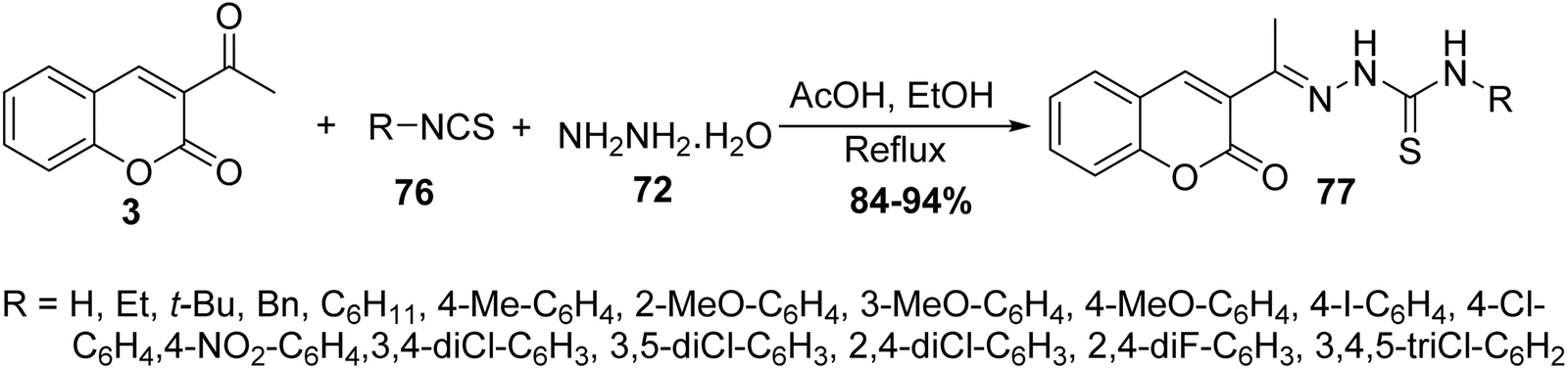
Synthesis of *N*-phenylhydrazine-1-carbothioamide derivatives 77.

#### 4.15.6. Reaction with urea/thiourea

The reaction of 3-acetylcoumarin 3 with urea and thiourea 78 afforded the Schiff base 79 when irradiated in under microwave in methanol (Scheme 44).^150^

**Scheme 44:**
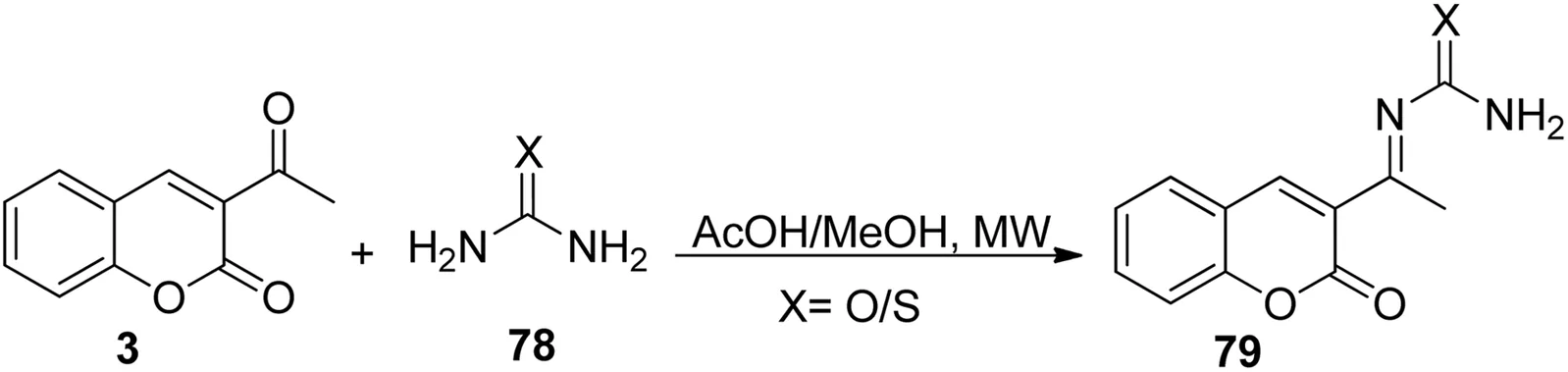
Condensation of 3-acetylcoumarin 3 with urea derivatives 78.

#### 4.15.7. Reaction with hydrazide/thiohydrazide

The Schiff base 81 was achieved readily *via* the treatment of 3-acetylcoumarins 3 with hydrazide/thiohydrazide 80 in various mono or mixture refluxing solvents such as ethanol, acetic acid, and isopropanol under different conditions A–M (Scheme 45).^284–331^

**Scheme 45:**
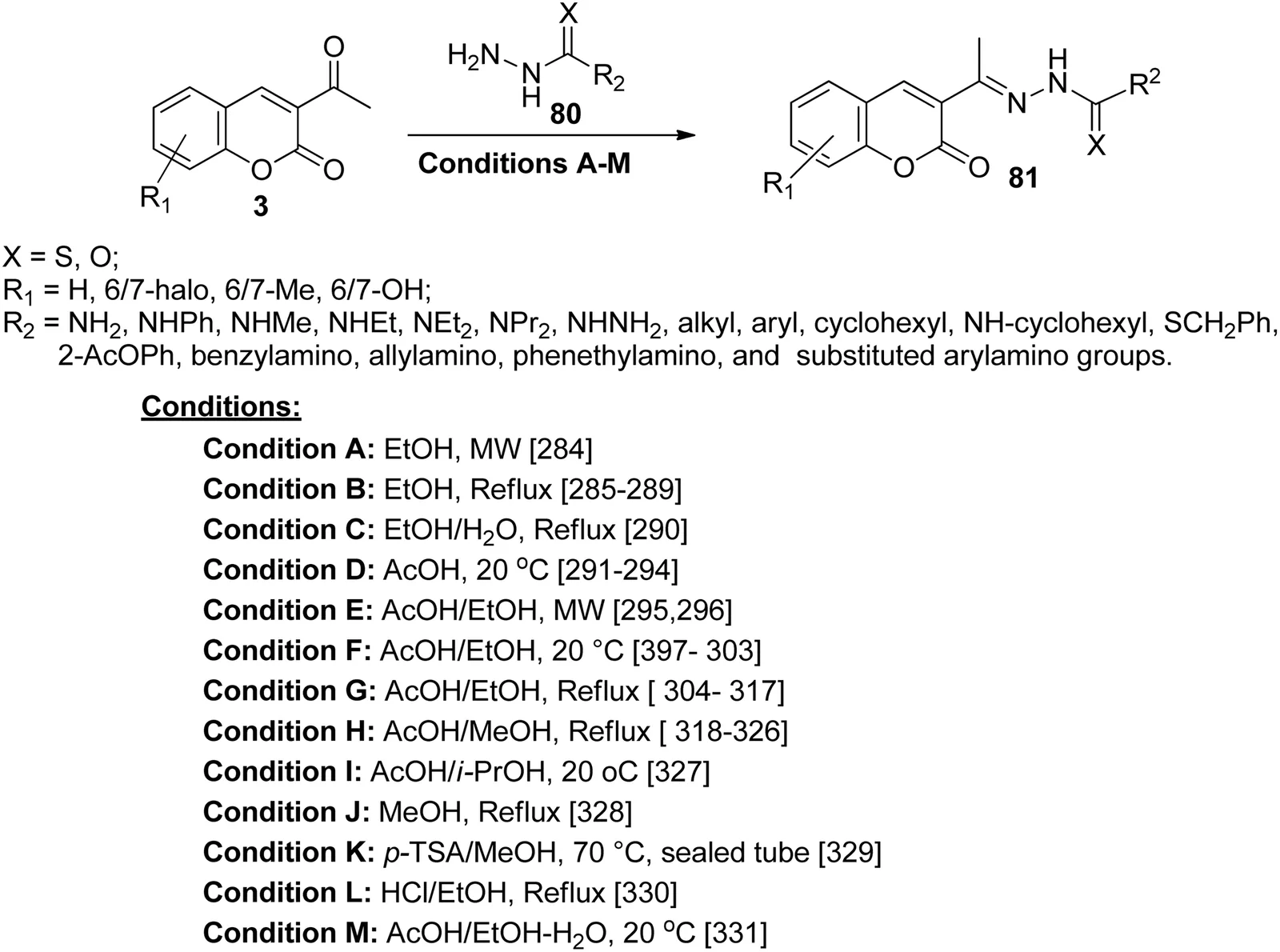
Condensation of 3 with 80 to afford Schiff base 81.

#### 4.15.8. Reaction with acid hydrazide

The condensation reaction between 3 with various hydrazides 80 was smoothly achieved to afford the corresponding hydrazine derivatives 82 (Scheme 46) under convenient or ultrasonic conditions.^332–351^

**Scheme 46:**
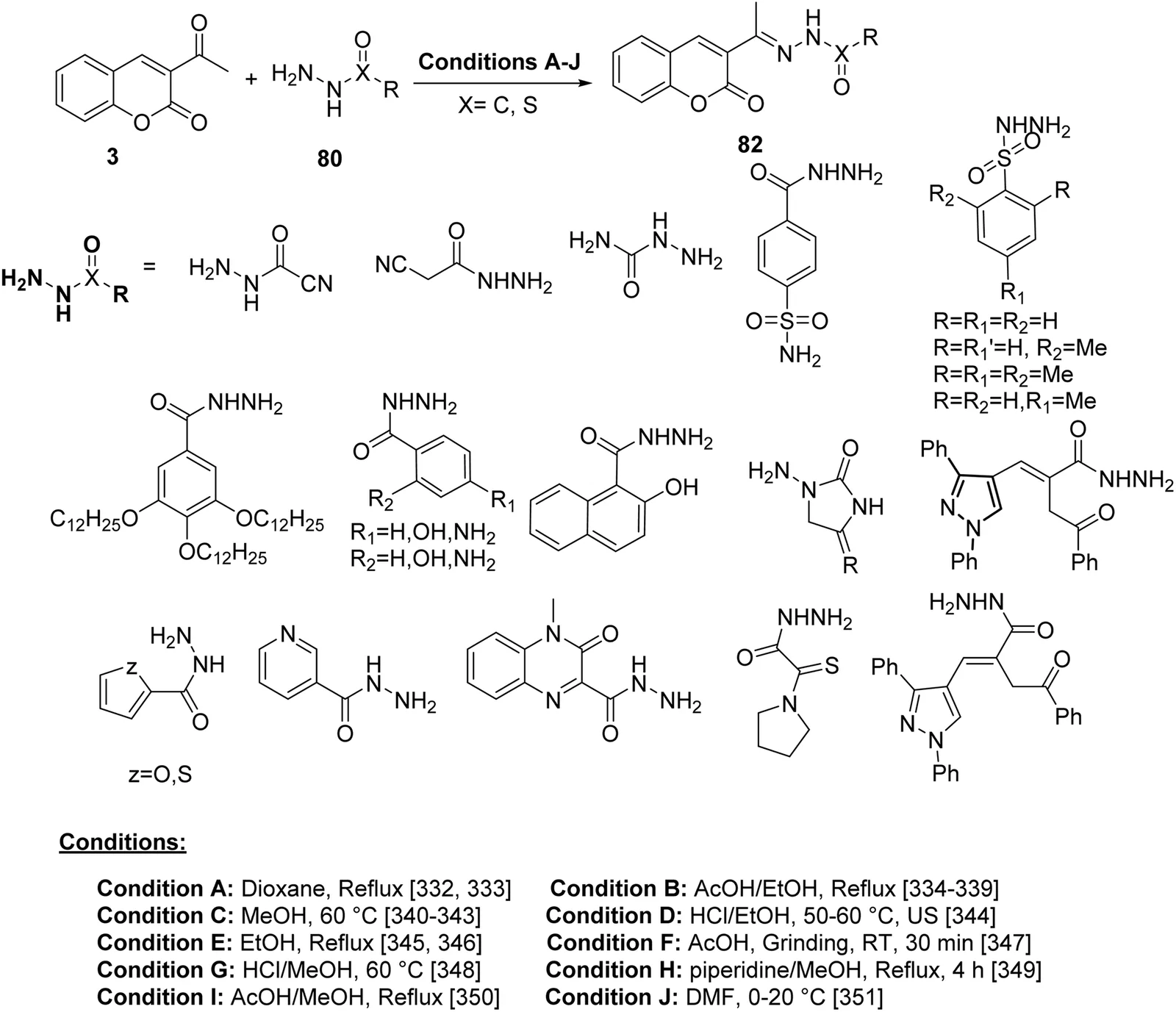
Condensation of 3-acetylcoumarin 3 with hydrazide derivatives 80.

Condensation of 3-acetylcoumarins 3 with thiocarbohydrazide 83 in ethanol/acetic acid gave the corresponding hydrazone derivatives 84 (Scheme 47).^314^

**Scheme 47:**
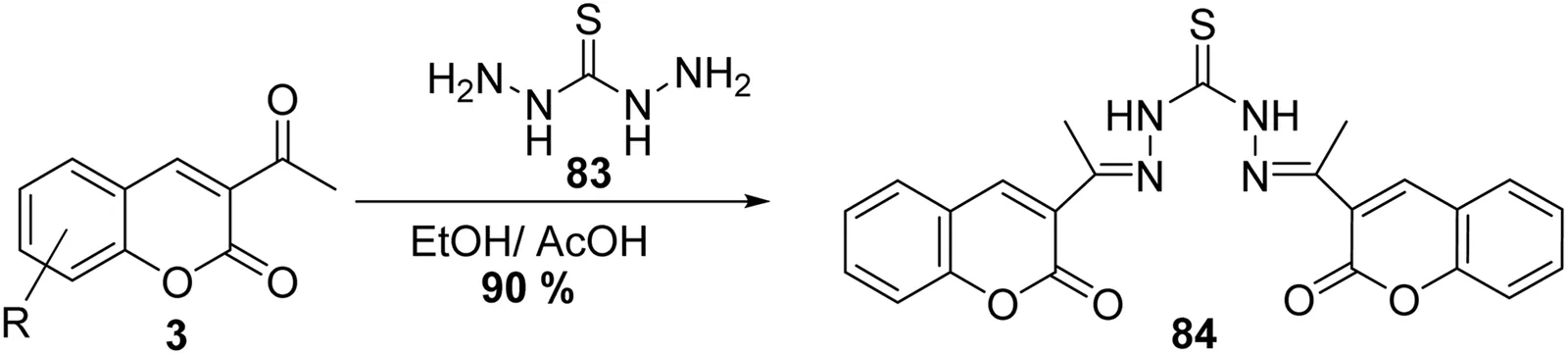
Synthesis of 3-acetylcoumarin-hydrazone 84.

### 4.16. Condensation with aldehydes and ketones

3-Acetylcoumarin 3 reacted with aldehydes 8 employing various solvents (*n*-butanol, dichloromethane, chloroform, acetic acid) and catalysts (piperidine, pyrrolidine, piperazine, triethylamine, potassium hydroxide, sodium hydroxide, sulfuric acid, silica sulfuric acid (SSA), TsOH, amberlyst 26A, cellulose sulphonic acid (CSA), bismuth(iii)nitrate/zinc(ii)chloride/alumina), bismuth(iii)trifluoromethanesulfonate (Bi(OTf)_3_) and Cs-doped tungstophosphoric acid (Cs-HPW) to give the corresponding α,β-unsaturated ketonic derivatives 85 (Scheme 48).^155,210,352–430^

**Scheme 48:**
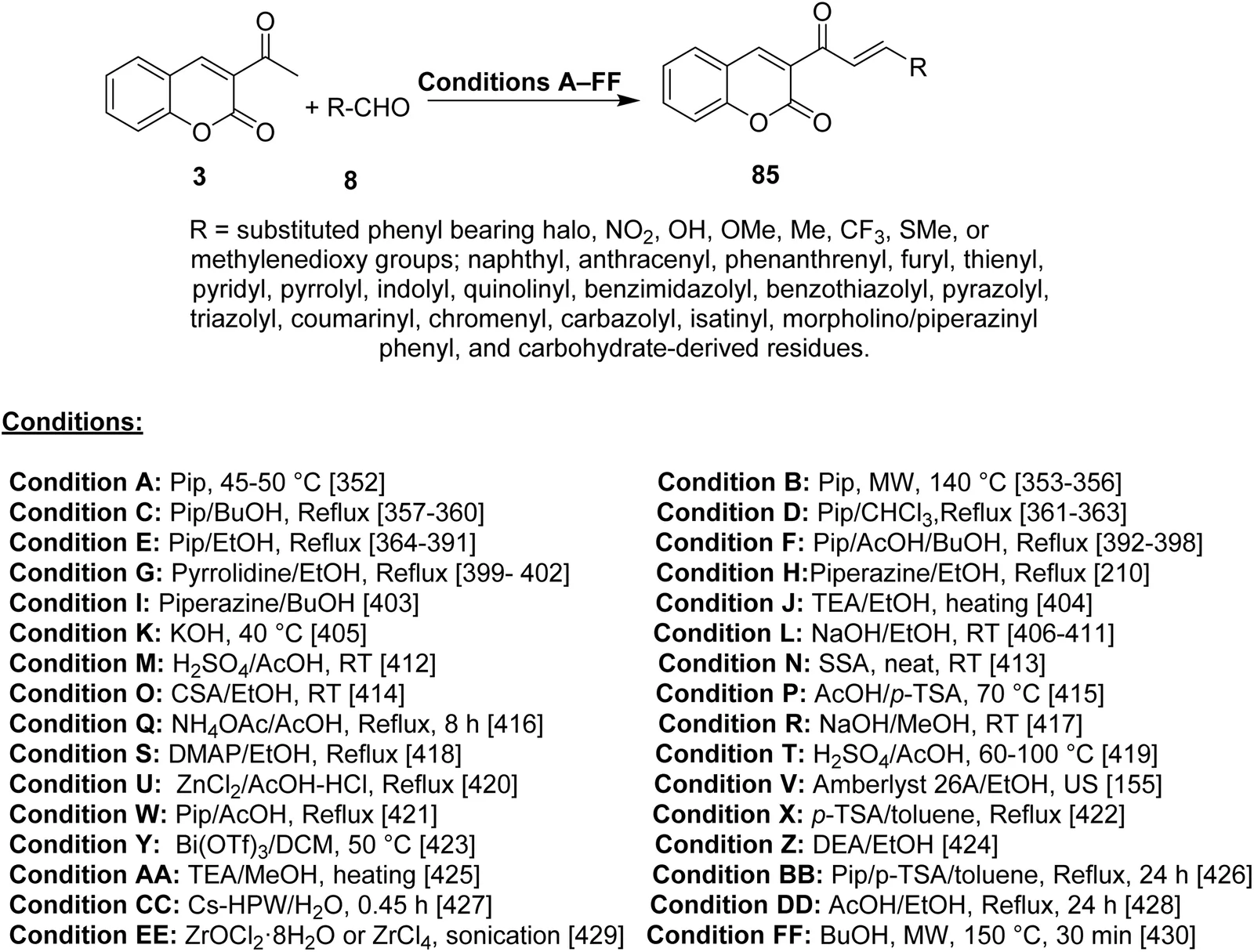
Substrate scope for preparation of α,β-unsaturated ketonic-coumarin derivatives 85.

## 5. Heterocyclic diversification of 3-acetylcoumarins

### 5.1. Five-membered heterocycles

#### 5.1.1. 5-Membered rings with one heteroatom

##### 5.1.1.1. Furo[3,4-*c*]coumarins

The reaction of 3 with isocyanide 86 was conducted in toluene under sealed-vessel conditions to minimize photooxidation or autoxidation by molecular oxygen. Under microwave irradiation, the process afforded the corresponding 2-imino-5-hydroperoxy-furo[3,4-*c*]coumarin derivatives 87.^431^ However, this under refluxing conditions in toluene yielded 5-hydroxy-*N*-substituted-2*H*-dihydrochromeno[3,4-*c*]pyrrol-2-ones 88. Whereby, using ethanol as a solvent instead of toluene, the fused chromeno-pyrrolone 89 was yielded under open vessel conditions as described by Ghandi^432^ nor the otherwise possible amino-substituted furo[3,4-*c*]coumarins 87 that was reported as the main products by Bazgir, the formation of 89 is based on the formation of quinone methides as a critical intermediate, followed by a [4 + 1]cycloaddition reaction with isocyanide 86 (Scheme 49).^431,433^

**Scheme 49:**
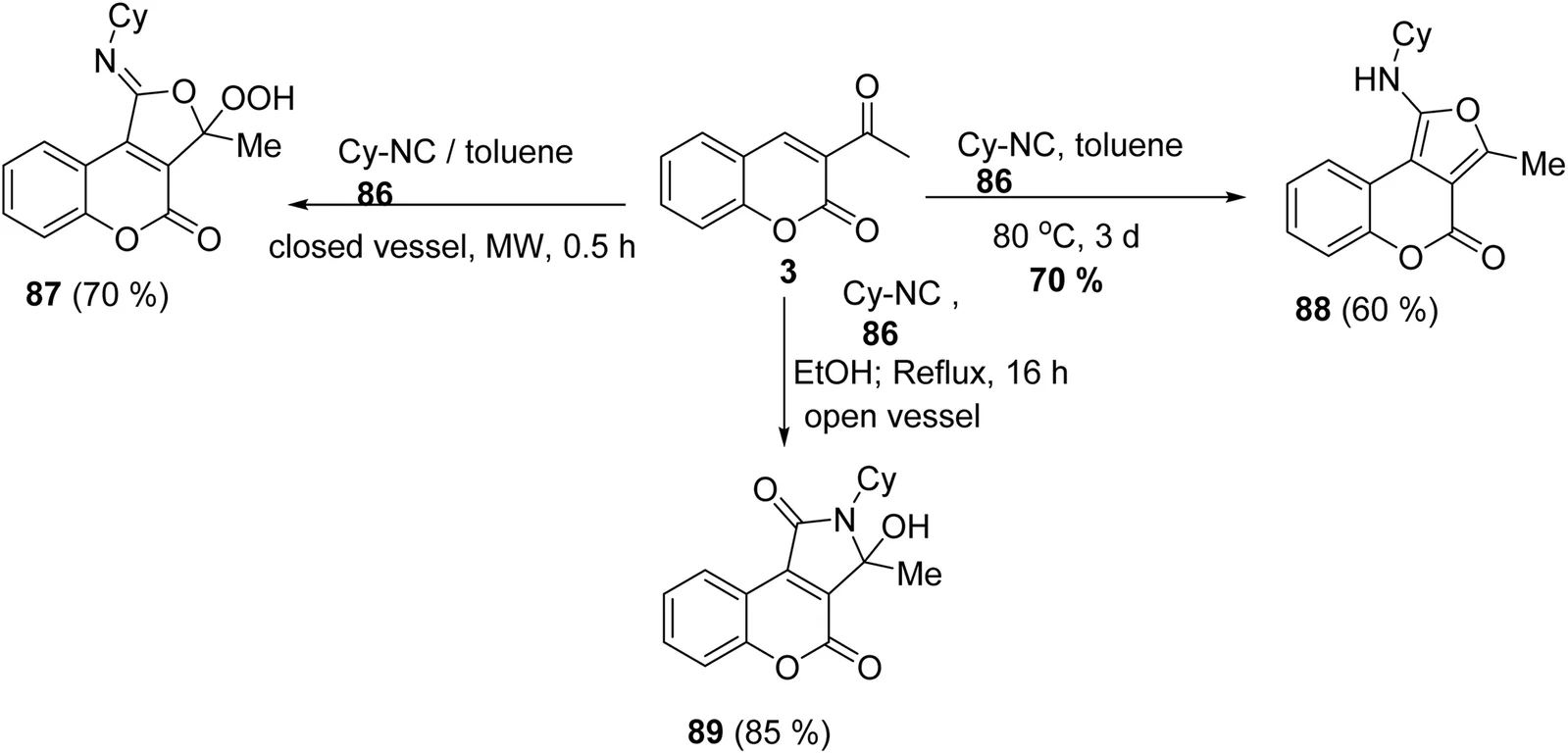
Heterocyclization of 3-acetylcoumarin 3 with isocyanide 86.

##### 5.1.1.2. Chromeno[3,4-*c*]pyrroles

Chromeno[3,4-*c*]pyrrole 90 was synthesized using a greener cycloaddition reaction of 3-acetylcoumarin 3 with van Leusen's reagent *p*-tolylmethyl isocyanide 86 in Van Leusen type [3 + 2] under catalyst-free conditions (Scheme 50).^434^

**Scheme 50:**
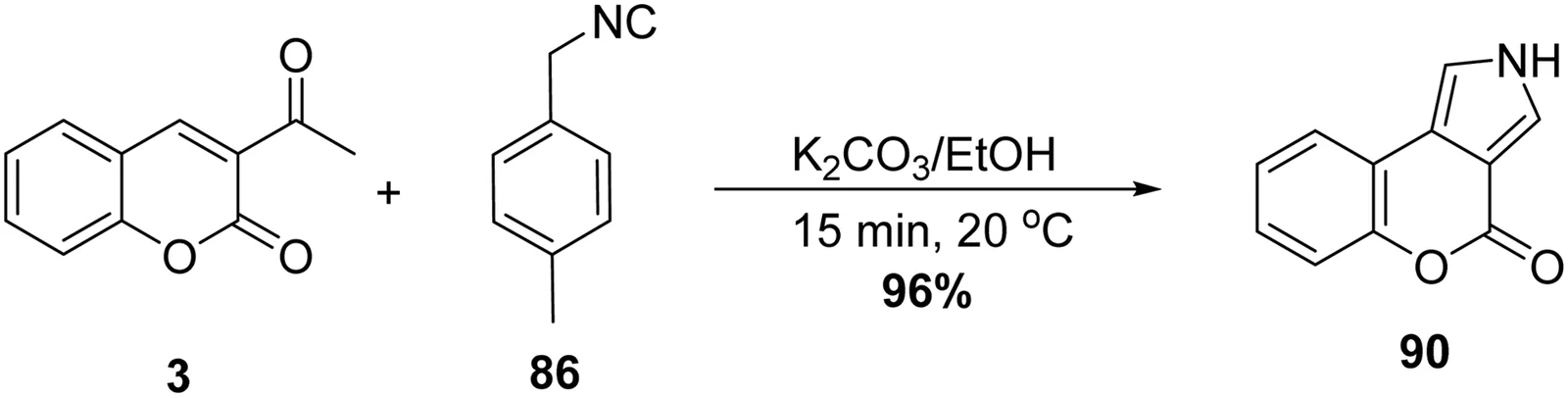
Synthesis of chromeno[3,4-*c*]pyrrole 90.

##### 5.1.1.3. Carbazole derivatives

A formal [1 + 2 + 3] annulation protocol of *o*-alkenyl aryl isocyanide 86 was disclosed under metal base or acid-free conditions with 3-acetylcoumarin 3 for synthesizing a wide range of carbazole derivative 91 (Scheme 51).^435^

**Scheme 51:**
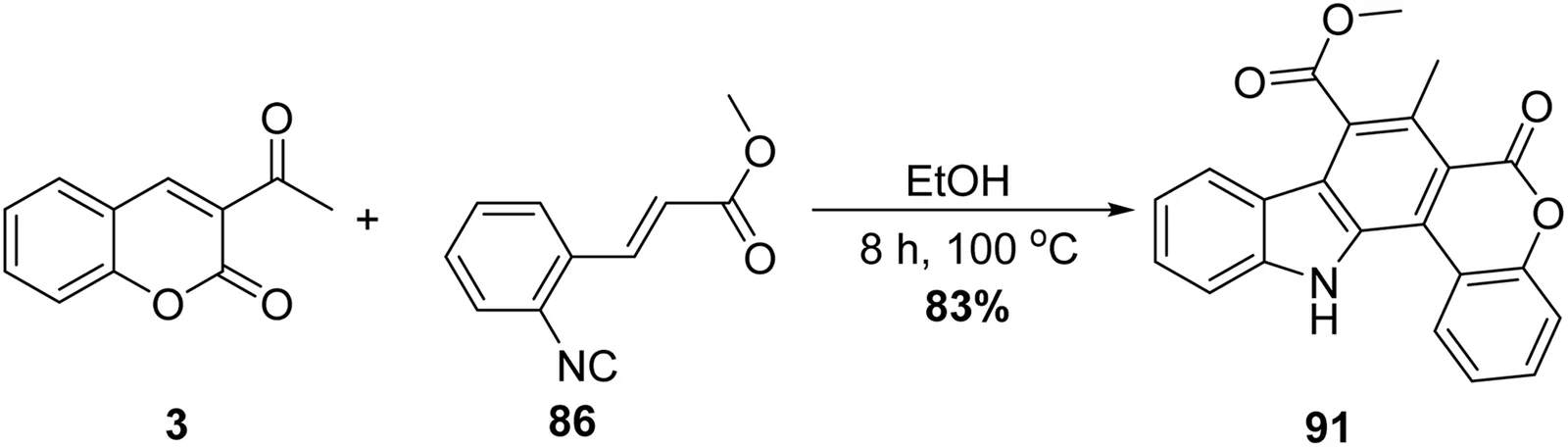
Synthesis of carbazole derivative 91.

##### 5.1.1.4. Coumarin–thiophene hybrids

Elemental sulfur (S_8_)^436,437^ or diethylamine^438^ were reported as the ideal condition for the one-pot, three-component reaction 3 and malononitrile 40 for the synthesis of coumarin–thiophene hybrids 92 as optical dyes with high thermal stability (Scheme 52).

**Scheme 52:**
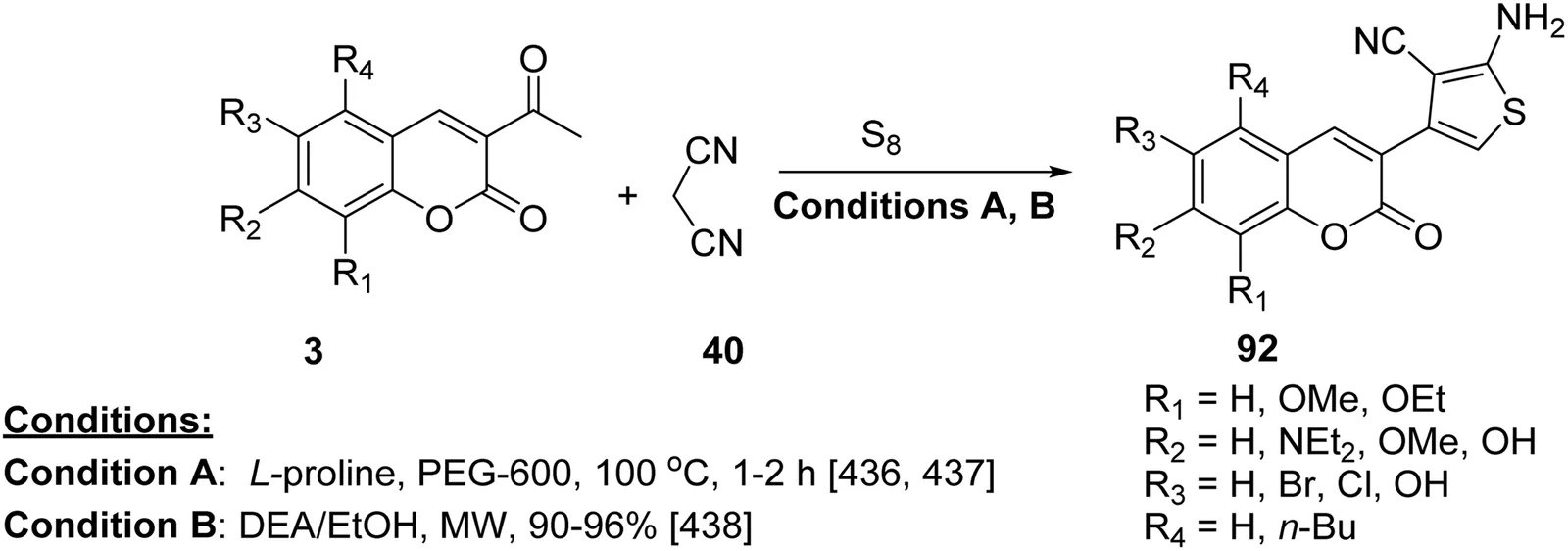
Synthetic route for coumarin-thiophene hybrids 92.

Novel 3-amino-4*H*-thieno[3,2-*c*]coumarins 93a–k were synthesized in 62–86% yields through a one-pot, catalyst-free three-component reaction of 3, amines 54, and elemental sulfur, using S_8_ as an efficient sulfur source under simple heating conditions (Scheme 53).^439^

**Scheme 53:**
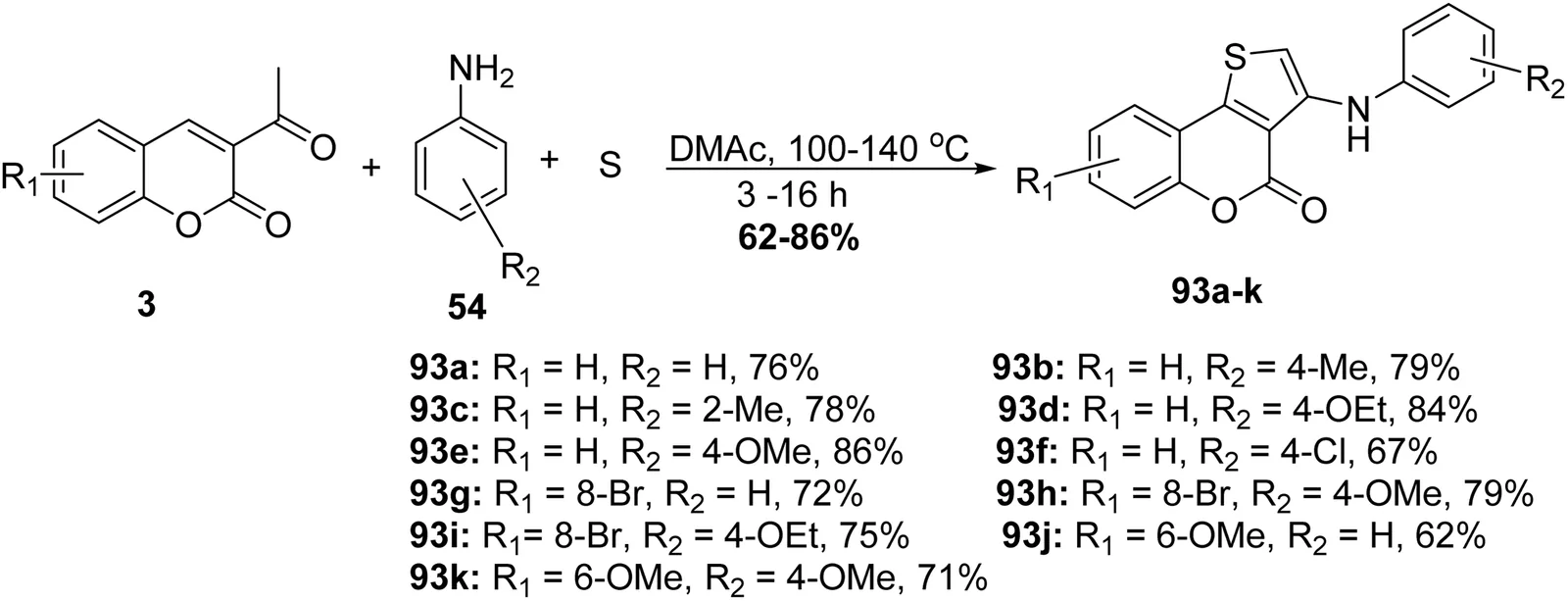
One-pot synthesis of 3-amino-4*H*-thieno[3,2-*c*]coumarins 93a–k.

##### 5.1.1.5. Selenophenes

One-pot synthesis of selenophenes 94a–c was achieved in reasonable yields and in a short time *via* the treatment of 3 with malononitrile 40 and selenium in ethanol in the presence of diethylamine (DEA) at ambient temperature (Scheme 54).^187^

**Scheme 54:**
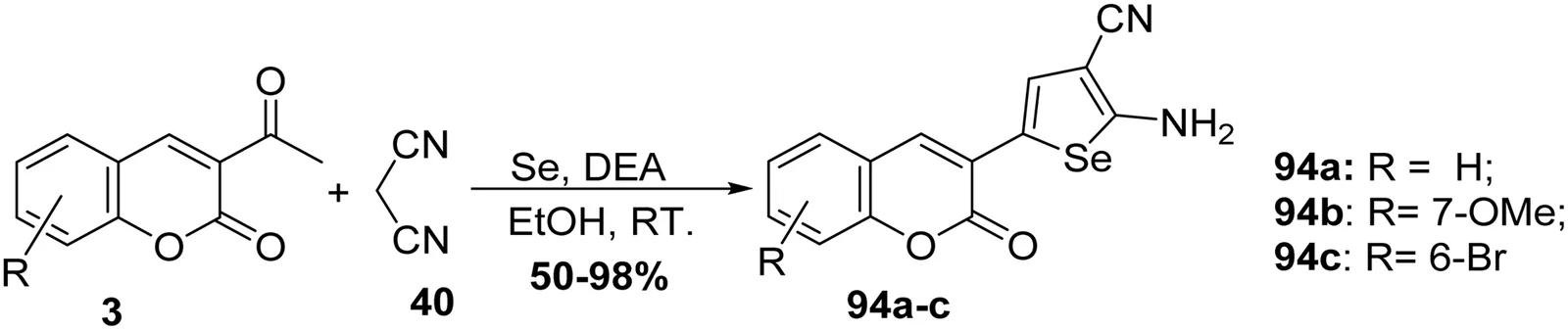
Synthesis of substituted selenophene-3-carbonitrile 94a–c.

#### 5.1.2. 5-Membered rings with two heteroatoms

##### 5.1.2.1. Pyrazole derivatives

Shaikh *et al.*^440^ reported a multicomponent reaction of 3, aldehydes 8, and hydrazines 74 in the presence of a morpholinium glycolate as a catalyst to yield pyrazole derivatives 95 (Scheme 55).

**Scheme 55:**
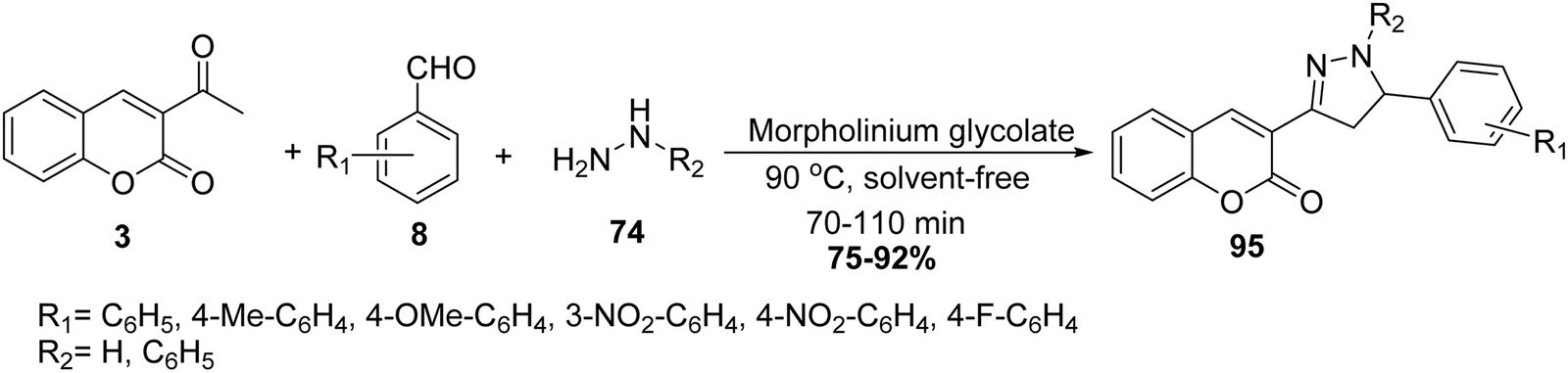
Synthesis of pyrazole derivatives 95.

Singla and Paul^441^ developed a one-pot domino strategy for synthesizing chromeno[4,3-*c*]pyrazol-4-ones 96, in which 3-acetylcoumarin 3, phenylhydrazine 74, and acrylates undergo a Ru(ii)-catalyzed regioselective *ortho*-alkenylation directed by a weak imine, using [RuCl_2_(*p*-cymene)]_2_ in combination with Cu(OAc)_2_·H_2_O in 1,2-dichloroethane at 120 °C; this approach allows the sequential formation of the pyrazole-fused chromene framework 96 in a single reaction vessel (Scheme 56).

**Scheme 56:**
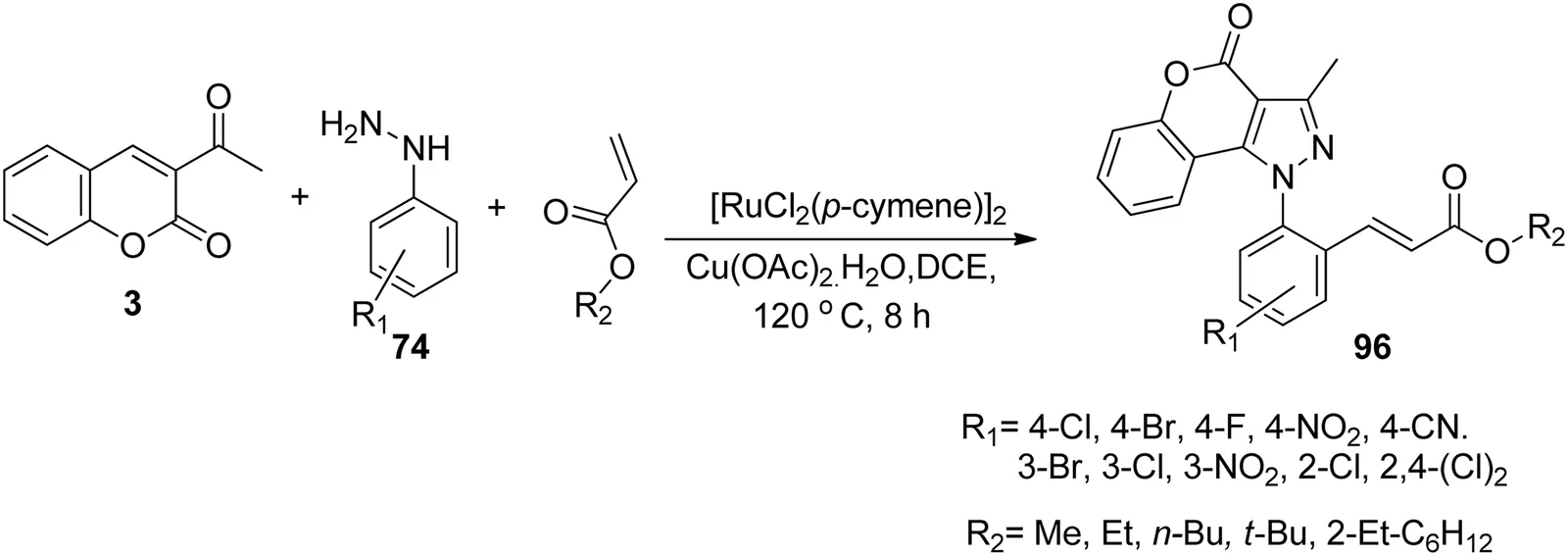
One-pot domino strategy for synthesizing chromeno[4,3-*c*]pyrazol-4-ones 96.

In another approach, pyrazole derivatives 97a–n were efficiently synthesized under solvent-free conditions using Fe_3_O_4_@MCM-41-SO_3_H as a magnetically reusable Lewis acid catalyst. Here, 3-acylcoumarins reacted with arylhydrazine derivatives 74 at 120 °C, providing high yields with a simple work-up and easy recovery of the catalyst (Scheme 57).^442^

**Scheme 57:**
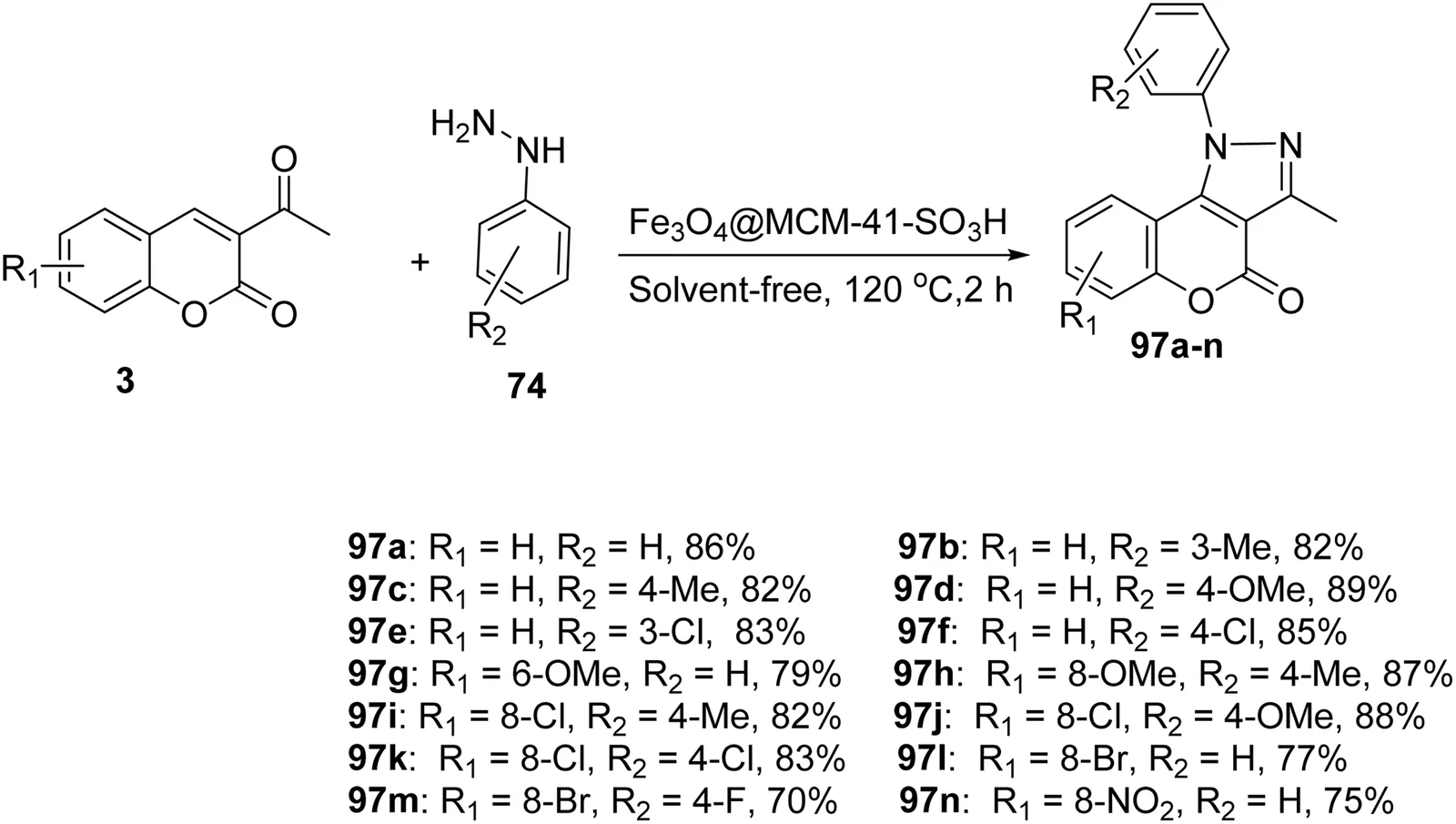
Solvent-free pyrazole synthesis using reusable Fe_3_O_4_@MCM-41-SO_3_H nanoparticles.

Additionally, reacting compound 3 with 3-amino-1*H*-pyrazol-5(4*H*)-one 98 in acetic acid afforded 2,3-dihydrochromeno[4,3-*d*]pyrazolo[3,4-*b*]pyridin-1,6-dione 99 in a similarly good yield (Scheme 58).^443^

**Scheme 58:**
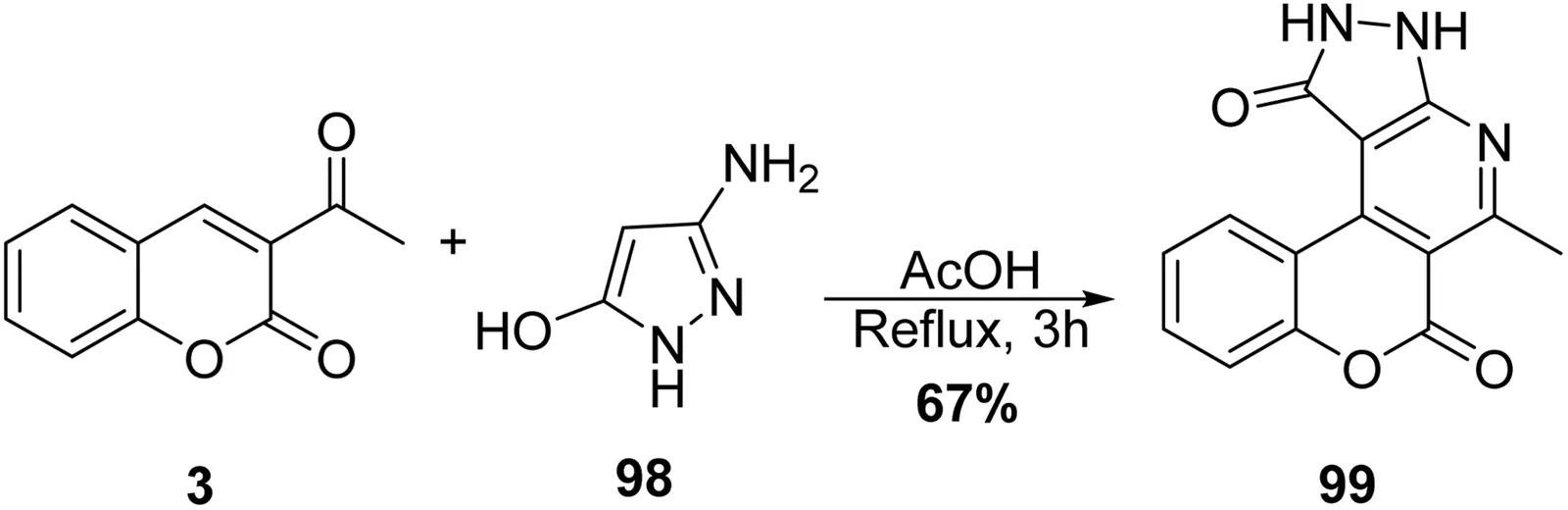
Reaction of acetylcoumarin 3 with 3-amino-pyrazol-5-one 98.

##### 5.1.2.2. Imidazolyl coumarin

3-Imidazolyl coumarin 101 was synthesized through [3 + 2] cycloaddition reaction of 3 and 2-aminopyridine 100 using iodine^444,445^ or iodine-ammonium acetate system^446^ as a catalyst. Compound 101 exhibited efficient dual luminescence behavior, showing strong blue fluorescence with the highest quantum yield as well as favorable yellow fluorescence in the solid state (Scheme 59).^447^

**Scheme 59:**
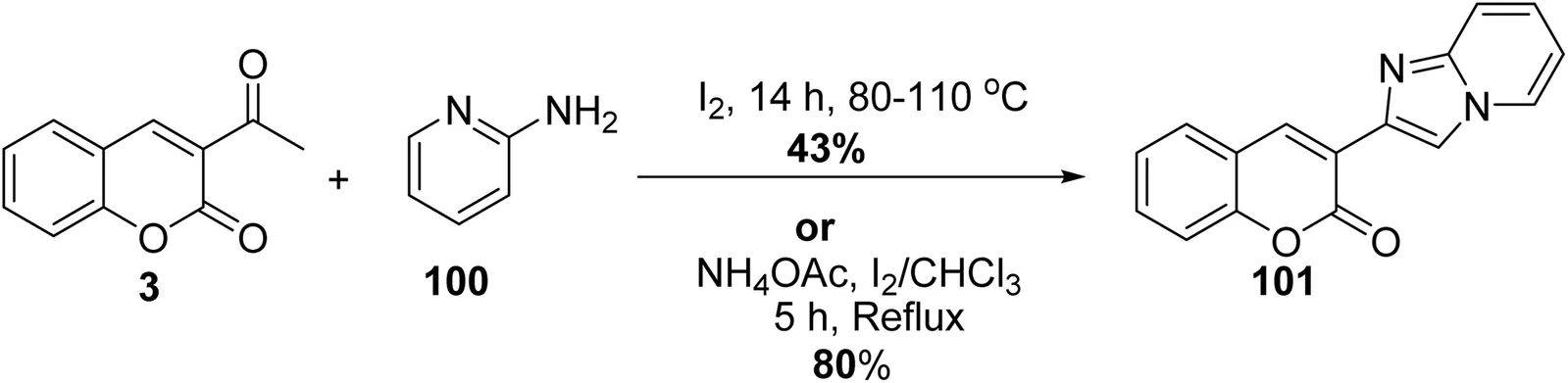
The formation of 3-imidazolyl coumarin system 101.

##### 5.1.2.3. Coumarin-oxazole/isoxazole derivatives

Wang *et al.*^448^ reported the synthesis of pyrrolo[3,4-*d*]isoxazoline 104 under metal-free conditions *via* a tandem radical functionalization/cycloaddition of 3-acetylcoumarin 3, *tert*-butyl nitrite 102, and maleimide 103 (Scheme 60).

**Scheme 60:**
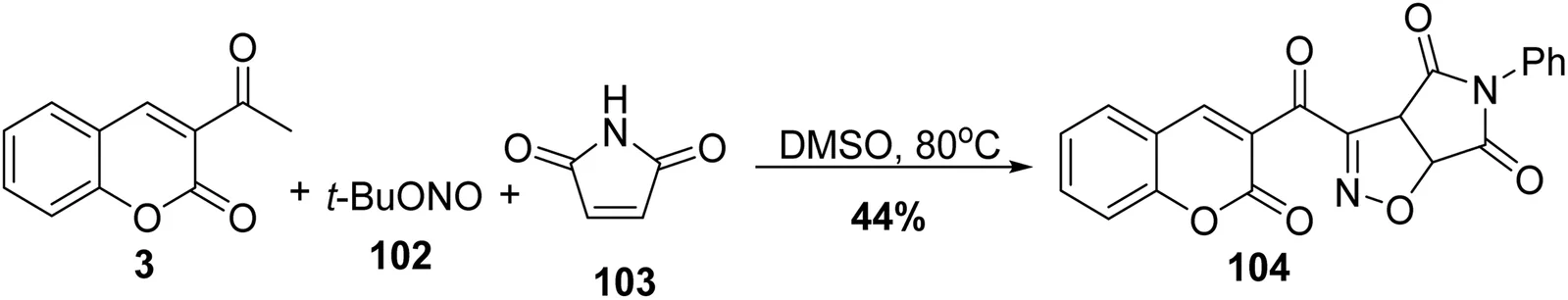
Synthesis of pyrrolo[3,4-*d*]isoxazoline 104.

##### 5.1.2.4. Thiazoles

Iodine catalyzed one-pot synthesis of substituted aminothiazoles 105*via* the reaction of 3-acetylcoumarins 3 with *N*-substituted thioureas 78 in absence of cupric oxide (Scheme 61).^449^

**Scheme 61:**
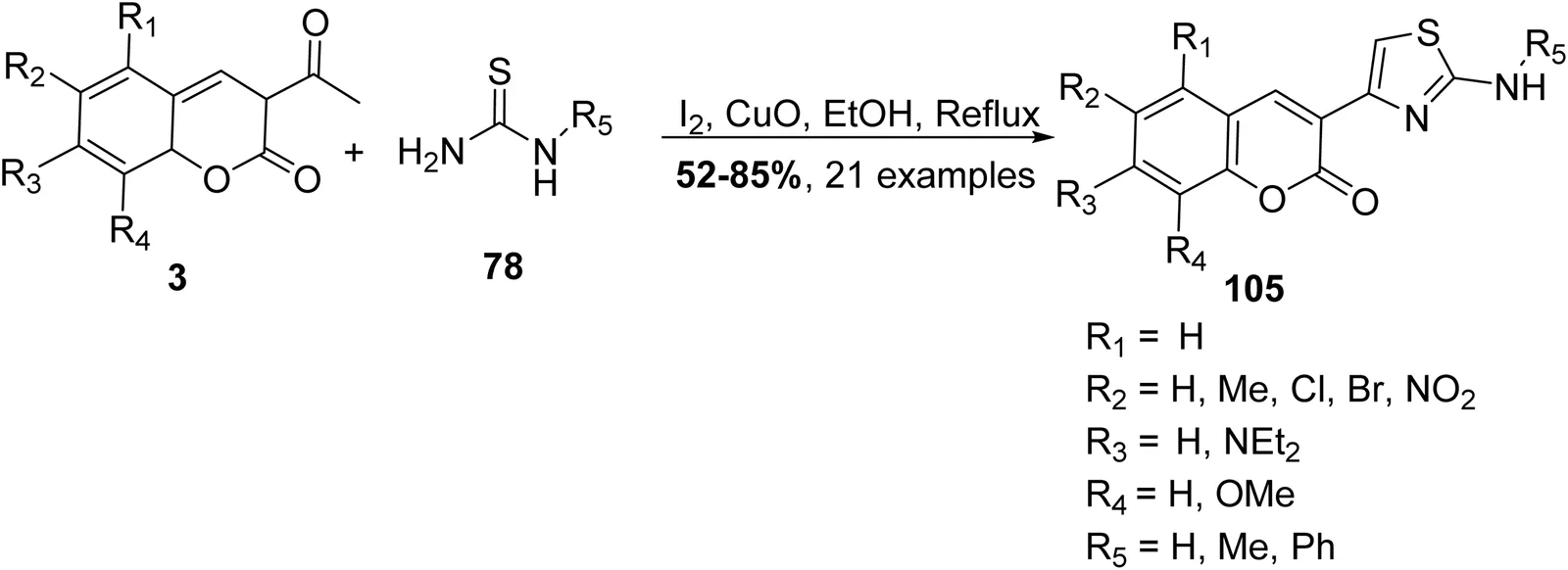
Preparation of 3-(2-arylamino-4-thiazolyl)-2-1-benzopyran-2-ones 105.

Coumarin-appended thiazole hybrids 106 were synthesized through the reaction of 3 with thiosemicarbazide 80 and substituted phenacyl bromides 34, affording 106 (Scheme 62).^450–454^ In some cases, methylimidazole propanesulfonic acid trifluoroacetic acid (MIMPS-CF_3_CO_2_H) was employed to promote the reaction.^455^

**Scheme 62:**
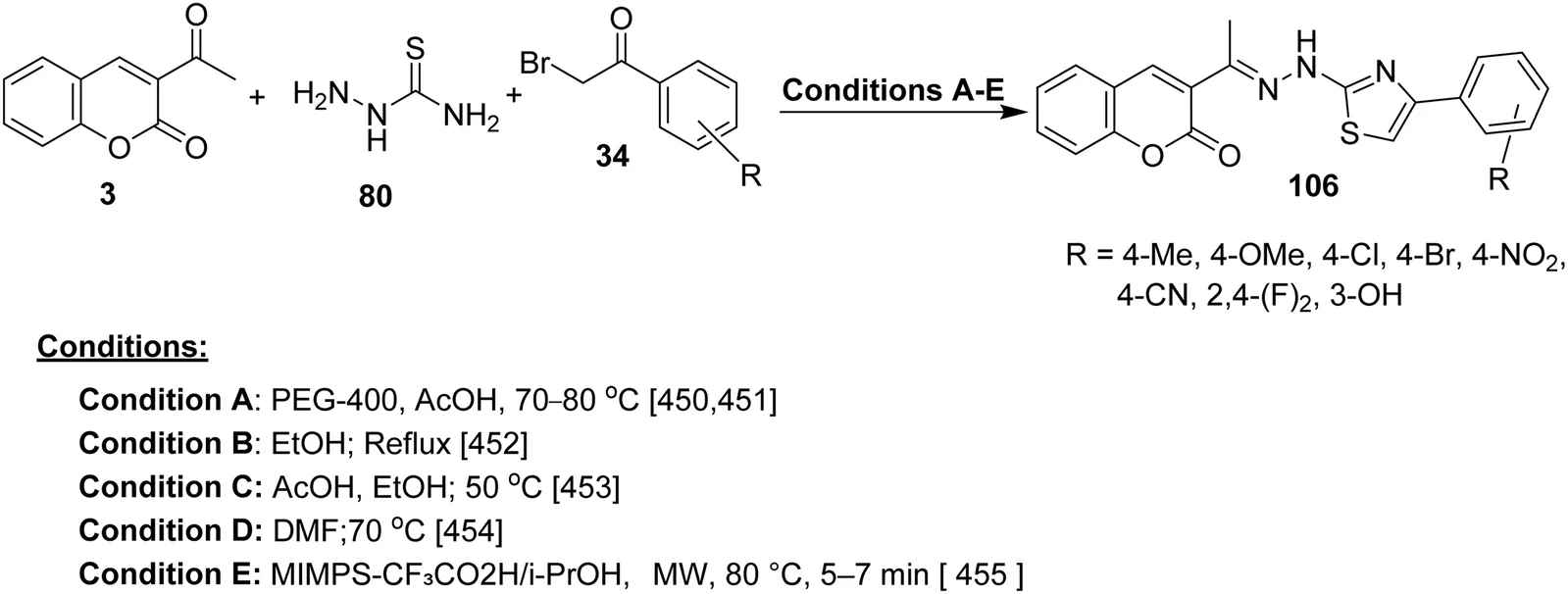
Synthesis of coumarin-appended thiazole hybrids 106.

In another related approach, Varluvothu *et al.* described a microwave-assisted one-pot multicomponent synthesis of hydrazonothiazolidinyl acetic acid derivatives 108 from substituted 3-acetylcoumarins 3, thiosemicarbazide 80, and maleic anhydride 107 using sodium acetate in acetic acid (Scheme 63).^456^

**Scheme 63:**
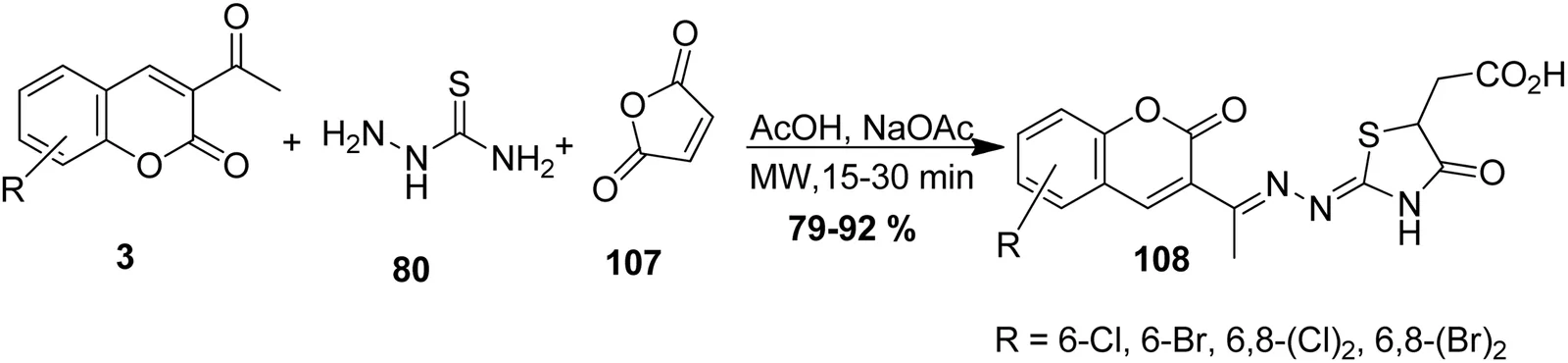
Microwave-assisted synthesis of hydrazonothiazolidinyl acetic acid derivatives 108.

An efficient one-pot three-component cyclization of 3, thiosemicarbazide 80, and dialkyl acetylenedicarboxylates 23 in acetic acid afforded alkyl 4-oxo-coumarinyl hydrazono-thiazolidin-5-ylidene acetates 109 in high yields (Scheme 64).^457^

**Scheme 64:**
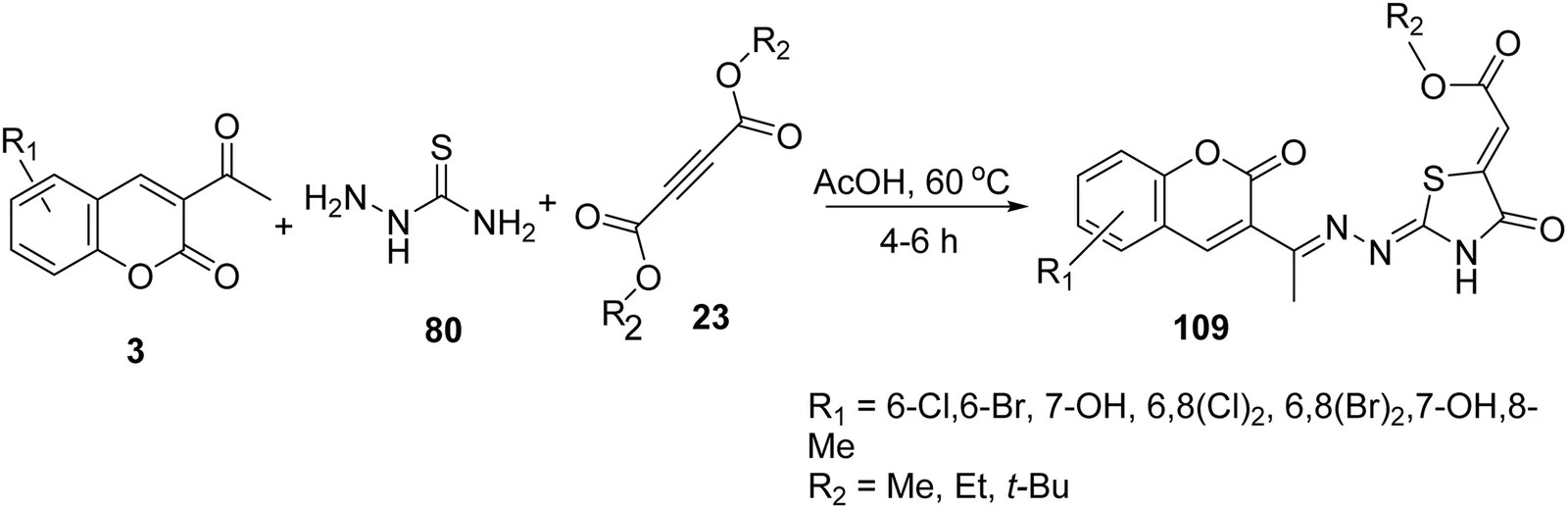
One-pot synthesis of 109.

#### 5.1.3. Five-membered rings with three heteroatoms

A one-pot, three-component route for the straightforward synthesis of 1,3,4-thiadiazole derivative 111 using 3-acetylcoumarin 3, thiocarbohydrazide 83, and a ketene *S*,*S*-acetal 110 in choline chloride-based deep eutectic solvent (Scheme 65).^458^

**Scheme 65:**
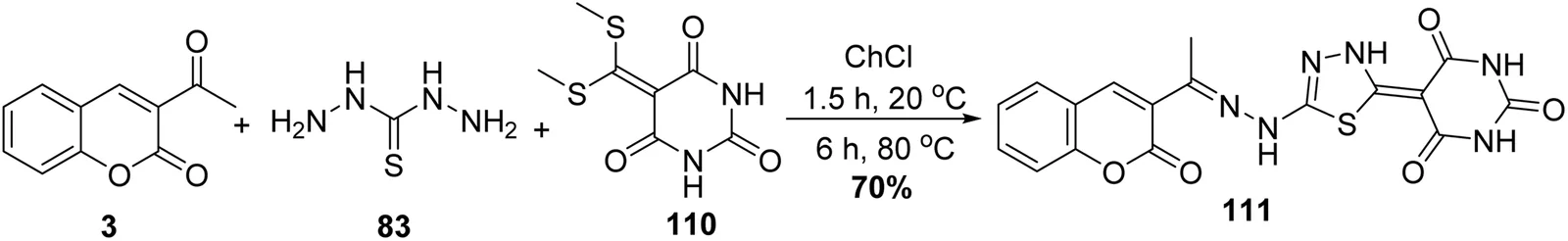
Synthesis of 1,3,4-thiadiazole derivative 111.

Channabasappa *et al.*^459^ reported a Cu(ii)-catalyzed oxidative coupling to access a series of coumarin-tethered 1,2,3-triazoles 113a–i from substituted azidobenzenes 112 and 3-acetylcoumarin 3. The transformation employs K_2_S_2_O_8_ as the oxidant and Cu(NO_3_)_2_ as the catalyst in DMF at 100–120 °C, with DMF (Scheme 66).

**Scheme 66:**
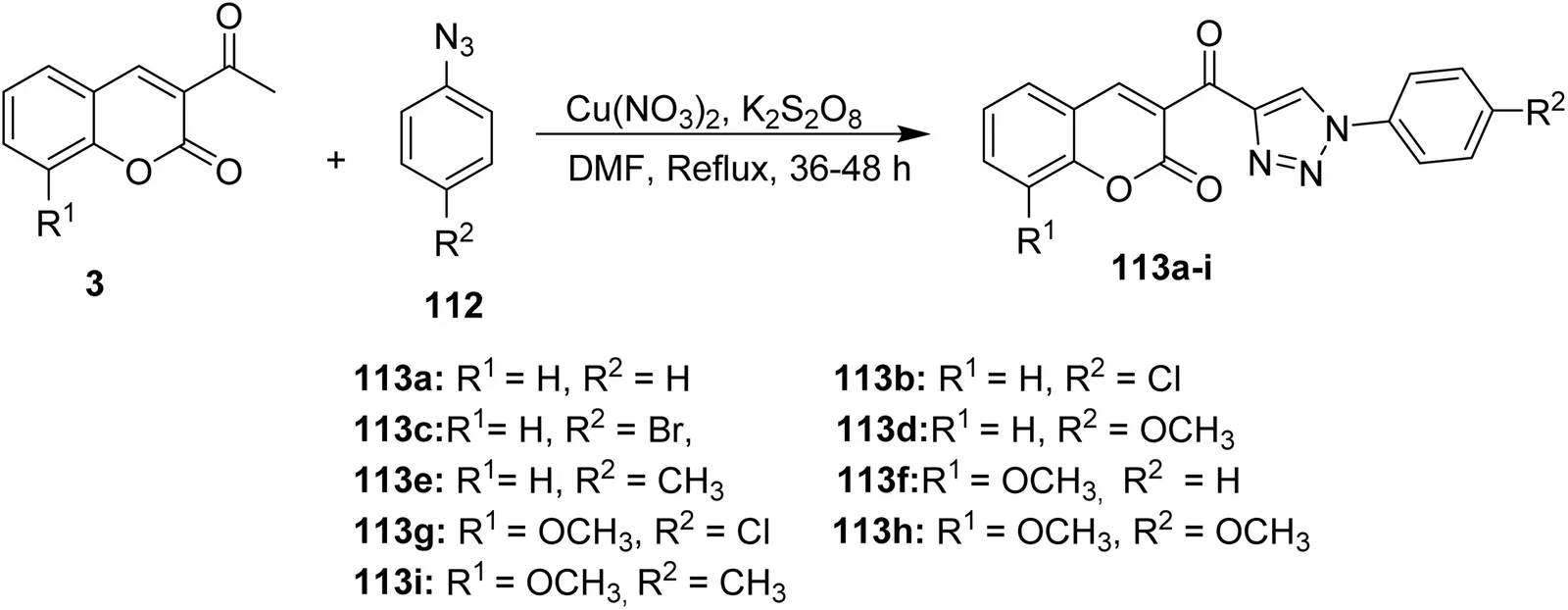
Cu(ii)-catalyzed oxidative coupling of azidobenzenes 113 with 3-acetylcoumarins 3.

### 5.2. Six-membered rings with one heteroatom

#### 5.2.1. Pyridocoumarins

Microwave-assisted cyclization of 3-acetylcoumarins 3 with ammonium acetate 41 in acetic acid afforded the fused pyridocoumarin derivative 114. This protocol provides an efficient route to (oxobenzopyranyl)benzopyranopyridinone derivatives under accelerated reaction conditions (Scheme 67).^460^

**Scheme 67:**
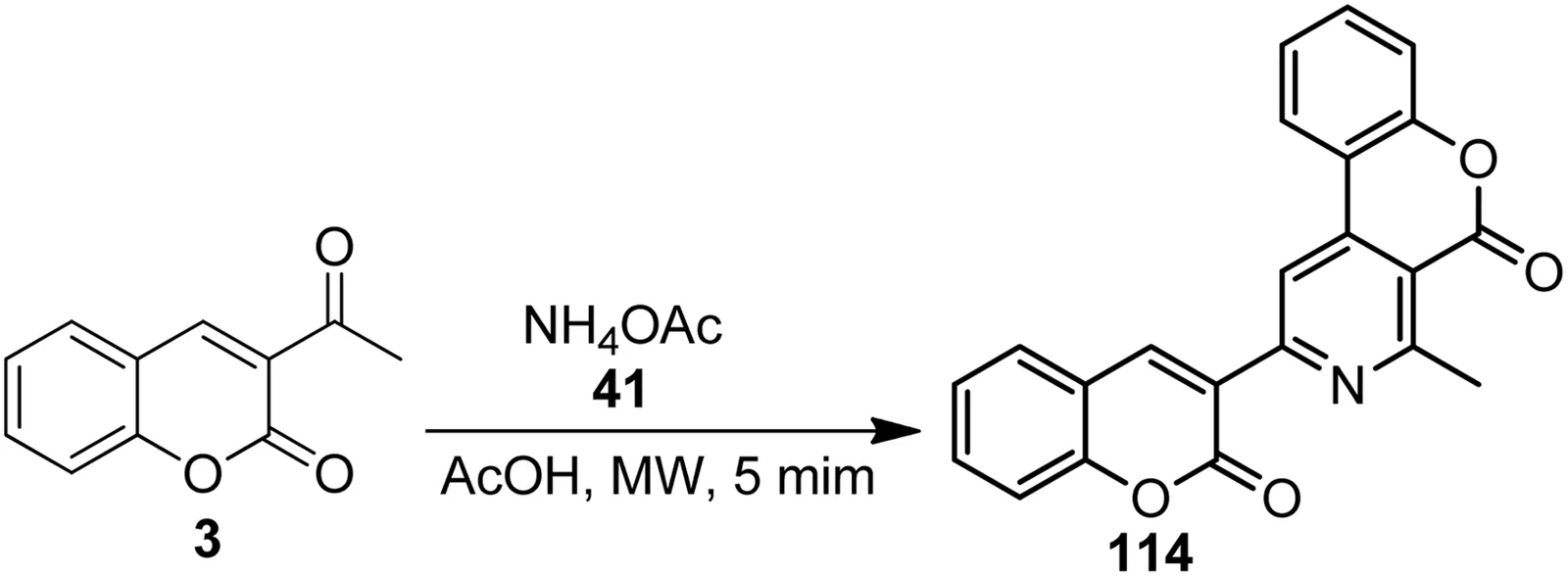
Synthesis of annulated pyridocoumarin 114.

The reactions of 3-acetylcoumarin 3 and ethyl cyanoacetate 115a, used as reagent and solvent, respectively, in the presence of cyanoacetamide 115b as an ammonia source at 170 °C, afforded 4-methyl-2,5-dioxo-3,5-dihydro-2*H*-chromeno[3,4-*c*]pyridine-1-carbonitrile 115b (Scheme 68).^461^

**Scheme 68:**
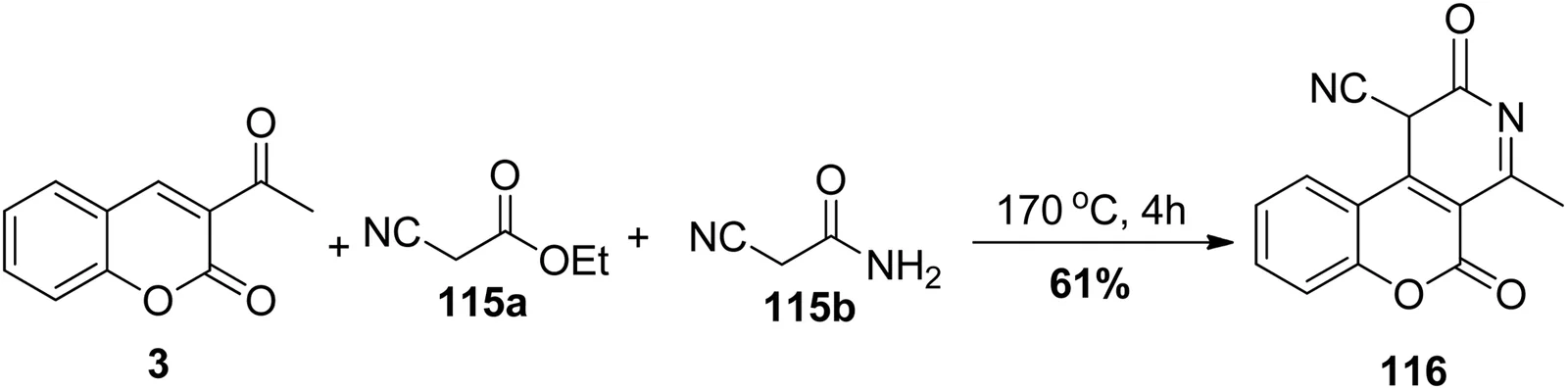
Synthesis of benzopyrano[3,4-*c*]pyridine scaffolds 116.

The reaction of substituted 3-acetylcoumarins 3 with 3-aminocrotonirile 117 in acetic acid under reflux led to the formation of 5-oxo-5*H*-chromeno[3,4-*c*]pyridine-1-carbonitriles 118 (Scheme 69).^462^

**Scheme 69:**
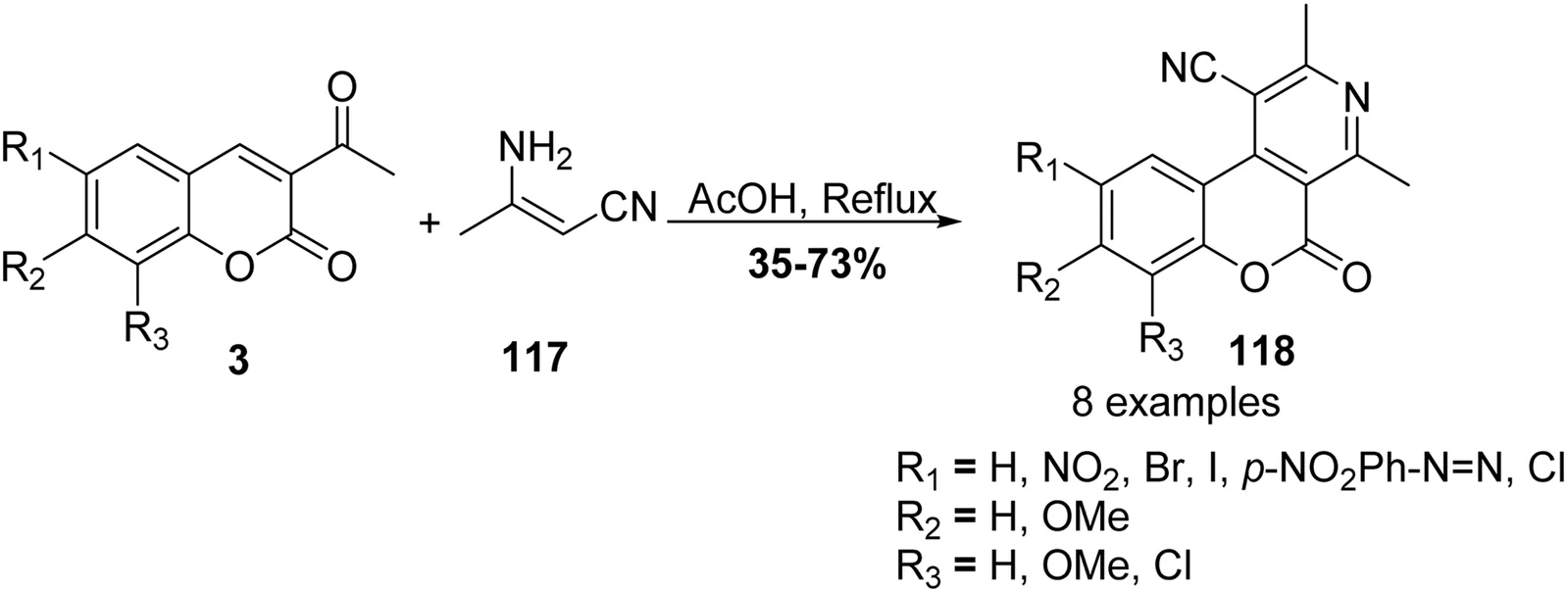
Synthesis of fused coumarin-pyridine scaffolds 118.

A simple and expeditious synthesis of series of 2,6-bis(1-coumarin-2-yl)-4-(4-substituted phenyl) pyridine derivatives 119 as a novel class of pH meters as well as organic electronics, antimicrobial and antioxidant agents has been achieved by one-pot, multicomponent reaction of 3-acetylcoumarins 3 with aryl aldehydes 8 in the presence of excess ammonium acetate 41 in refluxing acetic acid (Scheme 70).^463,464^

**Scheme 70:**
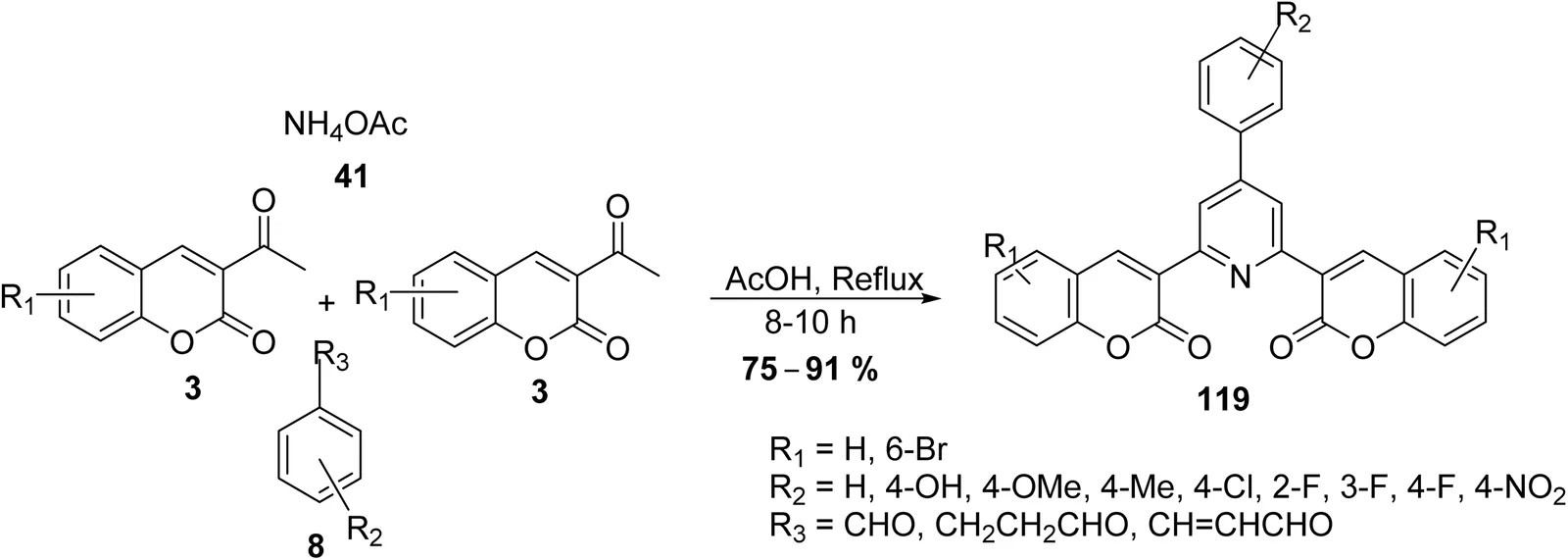
Multicomponent synthesis of coumarin-pyridines 119.

An efficient synthesis of novel tricoumarin-pyridines 121 has been described through the reaction of 3-acetylcoumarin 3 and 4-formyl coumarins 120 with ammonium acetate 41 for their usage in fluorescent dyes as well as their applications in organic electronics (Scheme 71).^465^

**Scheme 71:**
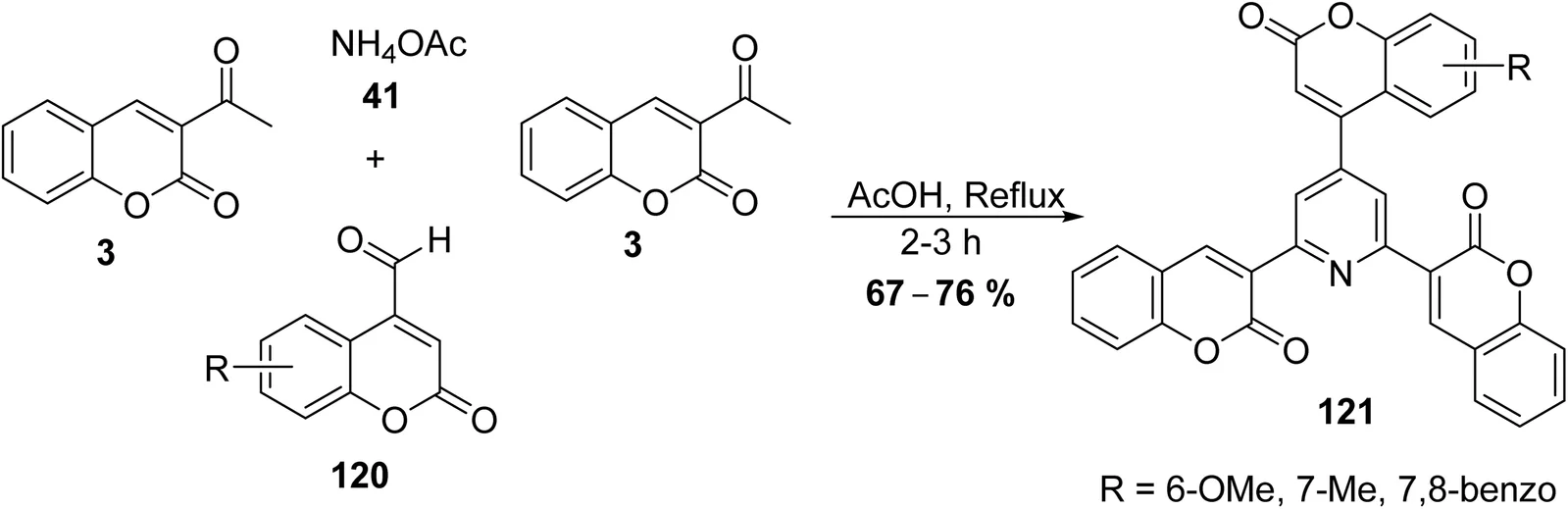
Synthesis route for 2,4,6-tricoumarin pyridines 121.

An effective strategy for the synthesis of a novel class of multifunctional indole and coumarin containing pyridine-3-carbonitriles 123 has been reported by Krishnammagari and co-workers^466^ through a one-pot four-component condensation reaction of 3-acetylcoumarin 3, 3-(1*H*-indol-3-yl)-3-oxopropanenitrile 122, aldehydes 8 and ammonium acetate 41 in refluxing acetic acid (Scheme 72).

**Scheme 72:**
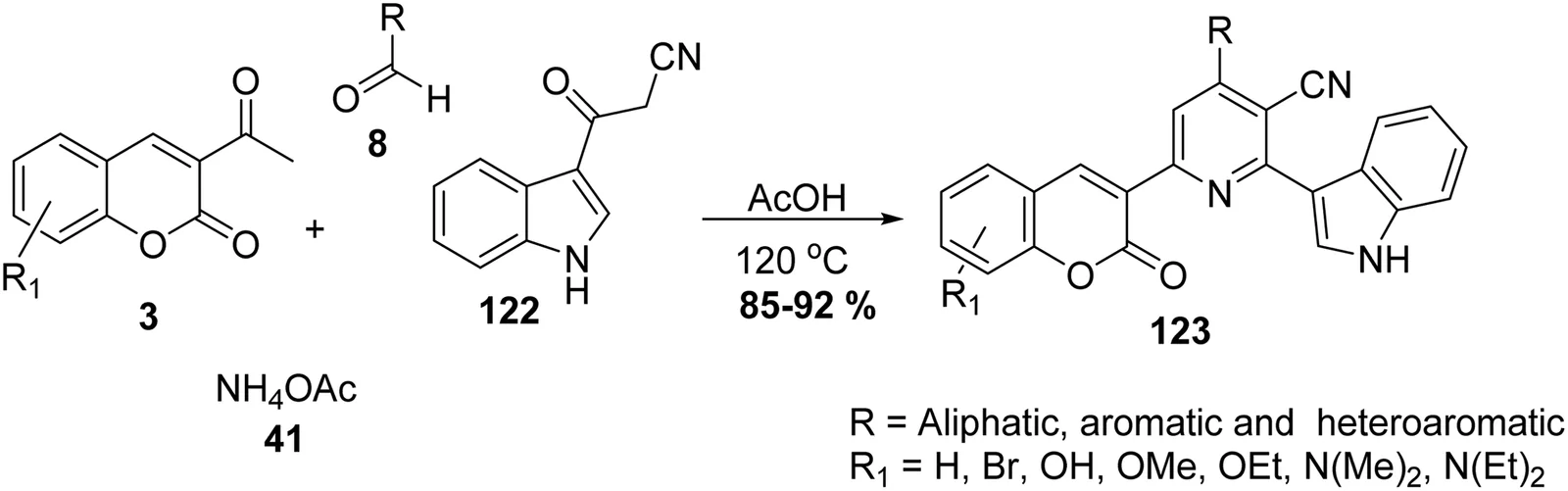
Synthesis of poly functionalized pyridine-3-carbonitrile derivatives 123.

The one-pot multicomponent condensation reaction of 3, various aldehydes 8, malononitrile 40, and ammonium acetate 41 under microwave-assisted and conventional heating afforded a facile entry into 3-(2′-amino-3′-cyano-4′-arylpyrid-6′-yl)coumarins 124 (Scheme 73).^467–473^ Many green and recyclable catalysts such as magnetic cellulose/Ag,^468^ zinc oxide-decorated superparamagnetic silica attached to graphene oxide (Fe_3_O_4_/SiO_2_/PTS-GO-ZnO),^474^ silica-supported perchloric acid (HClO_4_–SiO_2_)^475^ and iron(III) phosphate.^476^

**Scheme 73:**
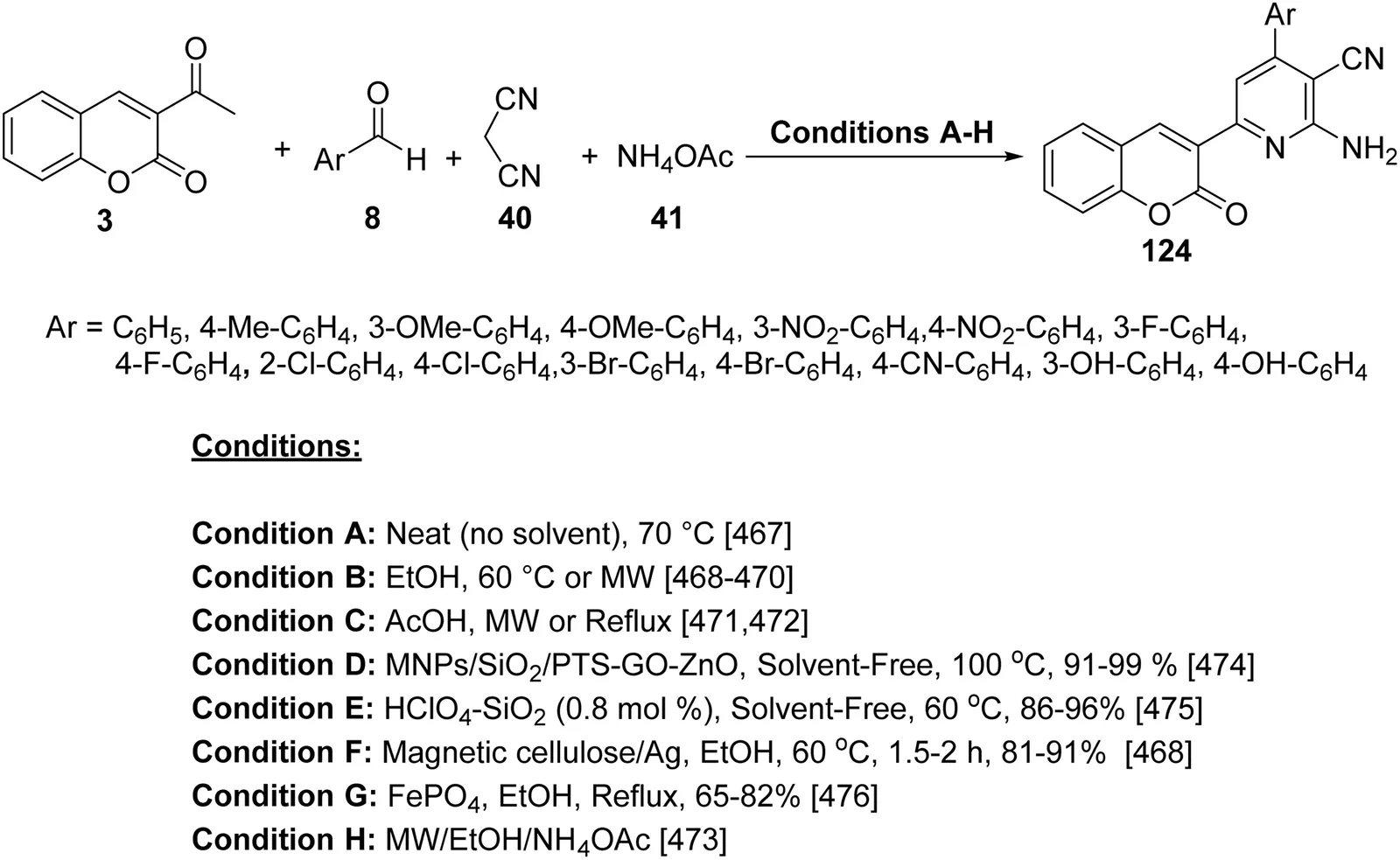
Synthesis of coumarin-linked nicotinonitriles 124.

One-pot multicomponent reactions (MCRs) of 3, various aromatic aldehydes 8, dimedone 125, and ammonium acetate 41 in the presence of chitosan sulfonic acid (CS-SO_3_H) as a biodegradable green catalyst under neat condition led to the formation of 7,7-dimethyl-2-(2-oxo-2*H*-chromen-3-yl)-4-aryl-7,8-dihydroquinolin-5(6*H*)-ones 126 in excellent yields (Scheme 74).^477^

**Scheme 74:**
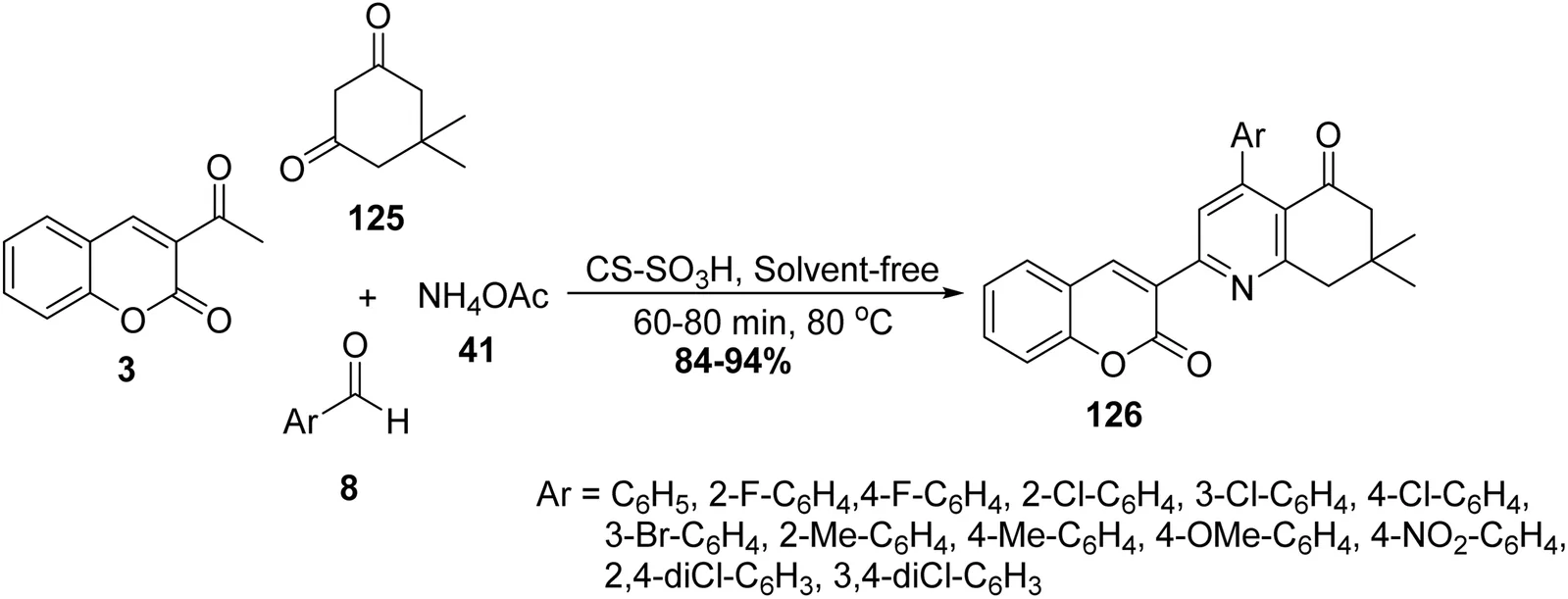
MCRs for the synthesis of 4-aryl-7,8-dihydroquinolin-5-one derivatives 126.

Convenient preparation series of chromeno[4,3-*d*]isoxazolo[5,4-*b*]pyridin-6-one systems 128a–d was achieved by the refluxing 3-acetylcoumarins 3 with 5-amino-3-methylisoxazole 127 in acetic acid (Scheme 75).^478^

**Scheme 75:**
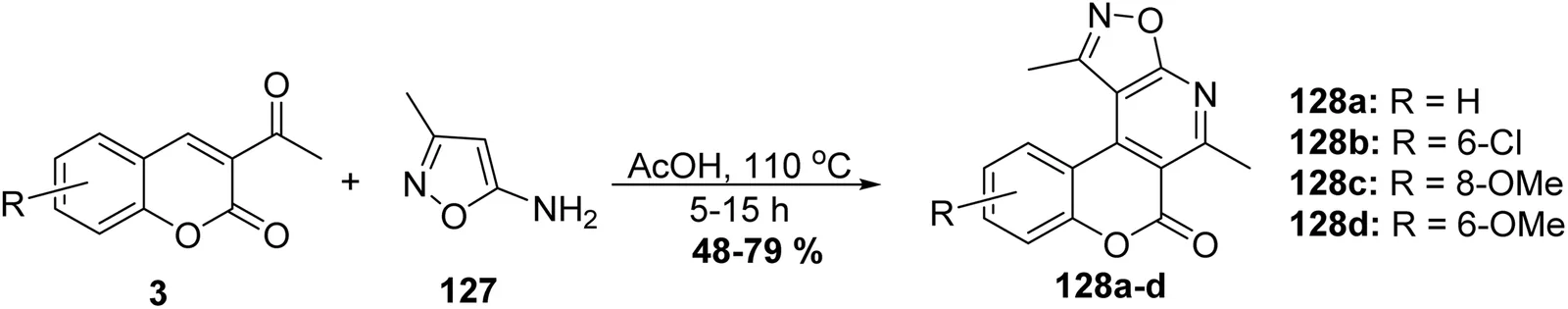
The synthesis of coumarin annulated by isoxazolo[5,4]pyridinones 128a–e.

Fused pyrido[4,3-*b*]coumarin derivatives 130 were efficiently prepared by condensation of 3-acetylcoumarins 3 with the 5-amino-pyrazoles 129 catalyzed by CuCl_2_·2H_2_O in ethanol^479^ or piperidine in refluxing acetic acid,^443^ affording a diverse series of pyrazolopyridochromene scaffolds 130 (Scheme 76).^479^

**Scheme 76:**
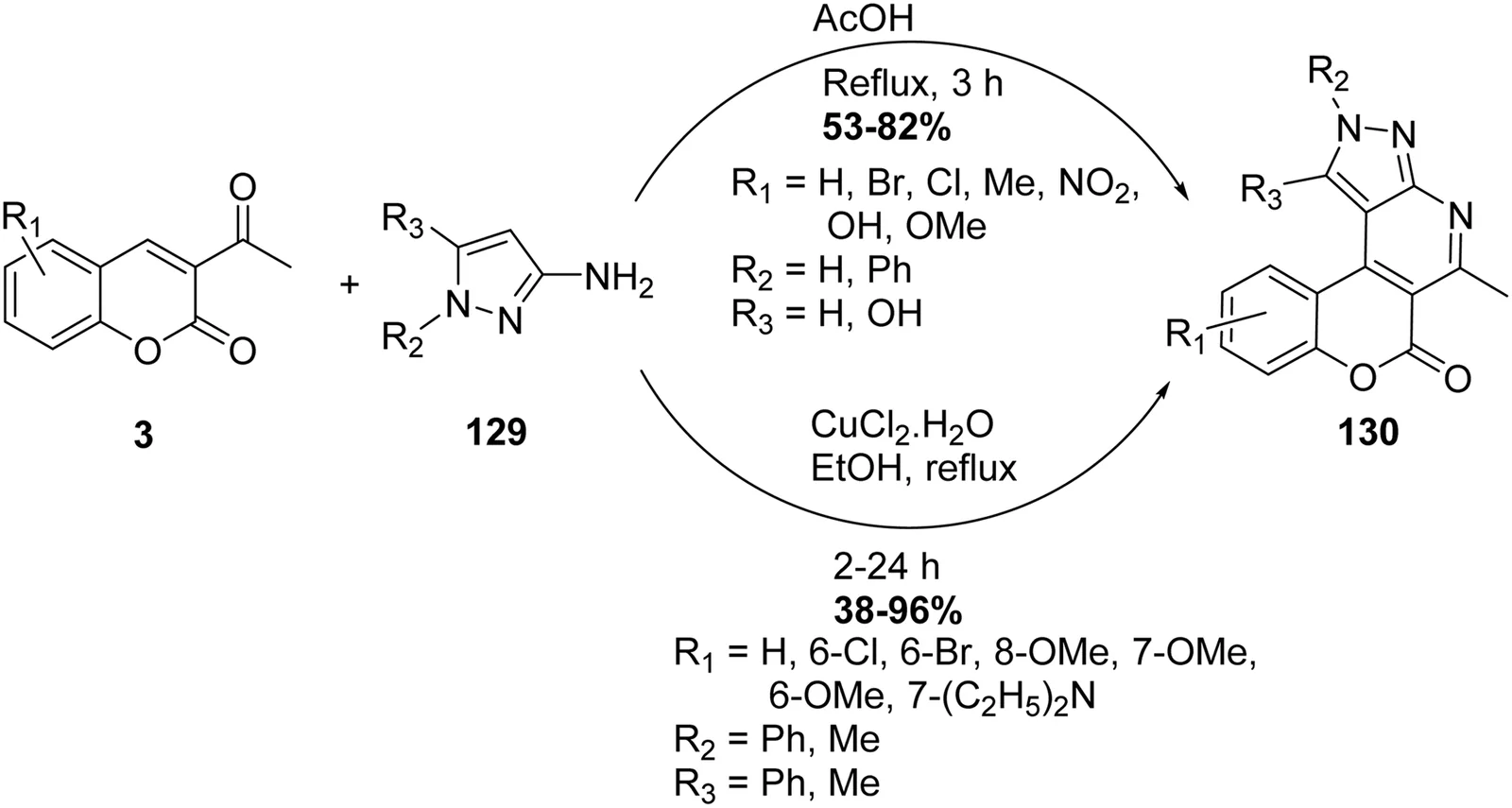
Synthetic routes for the preparation of pyrazolopyridochromene 130.

Diastereoselective for the three-component synthesis of fused pentacyclic compounds, chromeno[3′,4′:3,4]pyrido[2,1-*a*]isoquinoline derivatives 132, *via* a dipolar cycloaddition reaction of 3-acetylcoumarins 3, isoquinoline 131, and dialkyl acetylene dicarboxylates 23 (Scheme 77).^480^

**Scheme 77:**
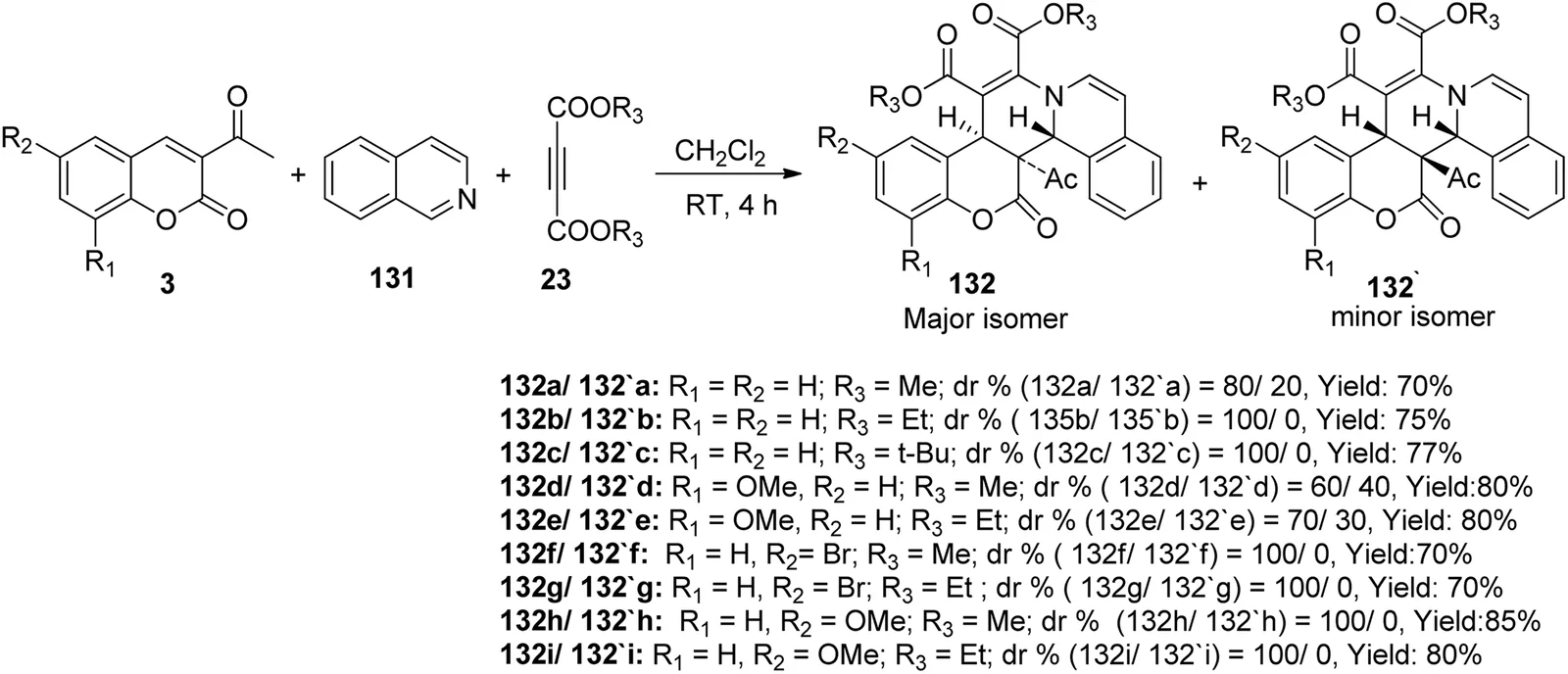
Synthesis of pentacyclic chromeno[3′,4′:3,4]pyrido[2,1-*a*]isoquinolines 132.

#### 5.2.2. Pyrimidines

Multicomponent reaction of 3 with 6-amino-2-thioxo-2,3-dihydropyrimidin-4(1*H*)-one 133 in refluxing acetic acid afforded 7-methyl-10-thioxo-chromeno[4′,3′:4,5]pyrido[2,3-*d*]pyrimidine-6,12-dione 134 (Scheme 78).^481^

**Scheme 78:**
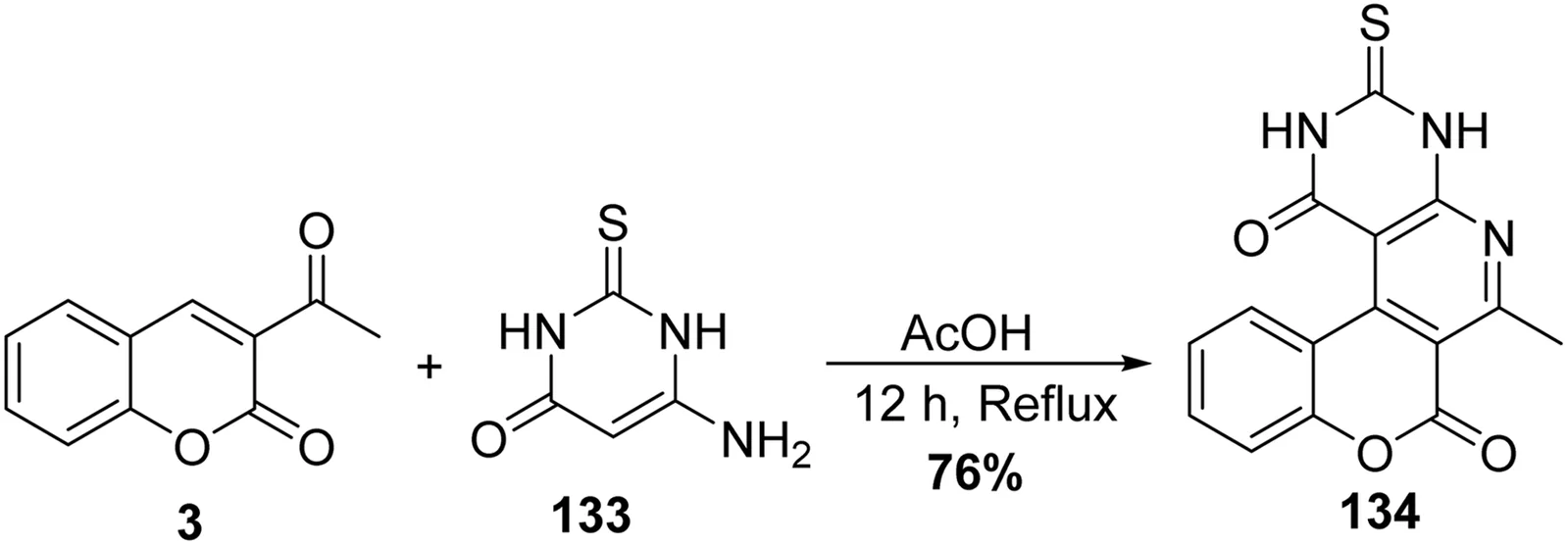
Synthesis of angular tetracyclic heterocyclic system 134.

Suresh *et al.*^482^ showed the ionic liquid-promoted multicomponent synthesis of fused tetrazolo[1,5-*a*]pyrimidine 136 as a promising α-glucosidase inhibitor *via* the multicomponent reaction of 3, 5-aminotetrazole 135 and DMF-DMA 70 in the presence of 1-butyl-3-methylimidazolium hydrogen sulfate [Bmim]HSO_4_ (Scheme 79).

**Scheme 79:**
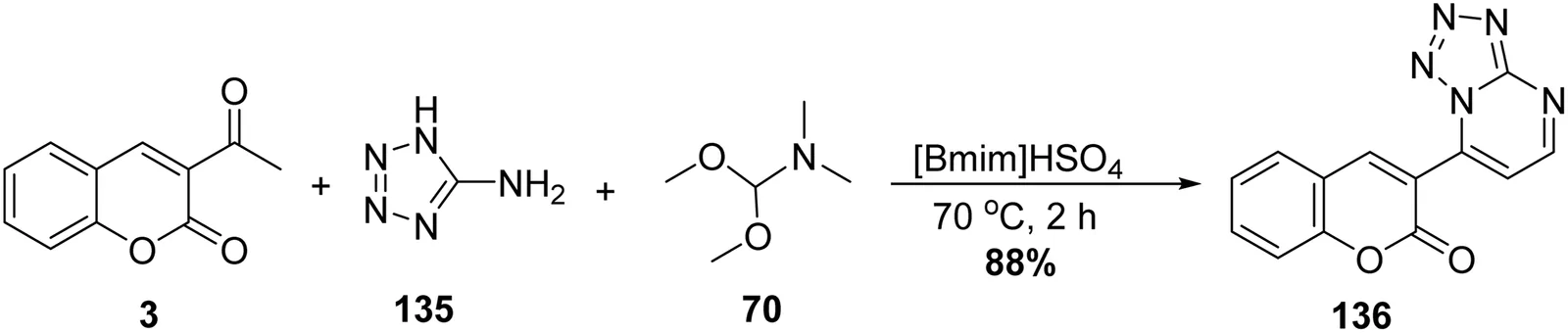
Synthesis of tetrazolo[1,5-*a*]pyrimidine 136.

#### 5.2.3. Pyranocoumarin derivatives

Grinding of 3 with ethyl-2-cyano-3,3-bismethylthioacrylate 137 in presence of potassium hydroxide under free solvent condition afforded 4-methylsulfonyl-2-oxo-6-chromene-3-pyran-3-carbonitrile 138 (Scheme 80).^483^

**Scheme 80:**
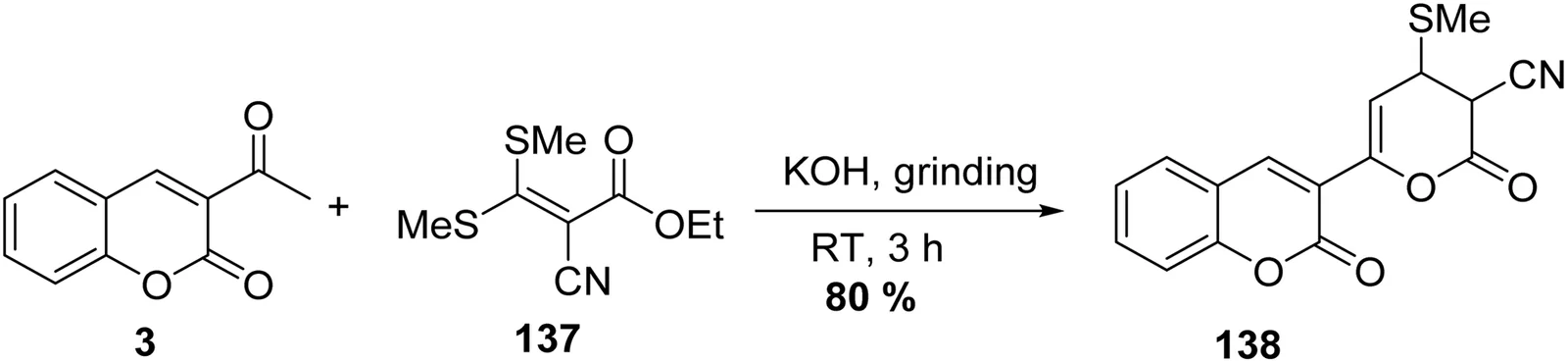
Treatment of 3 with 3,3-bismethylthioacrylate 137.

A facile and one-pot synthesis of series of (2-oxo-2*H*-chromen-3-yl)-4*H*-pyran derivatives 140 through a catalytic condensation between 3, malononitrile 40 or ethyl cyanoacetate 139, and aldehydes 8 using trimethylamine^484^ or tungstate sulfuric acid (TSA)^226^ as a catalyst (Scheme 81).

**Scheme 81:**
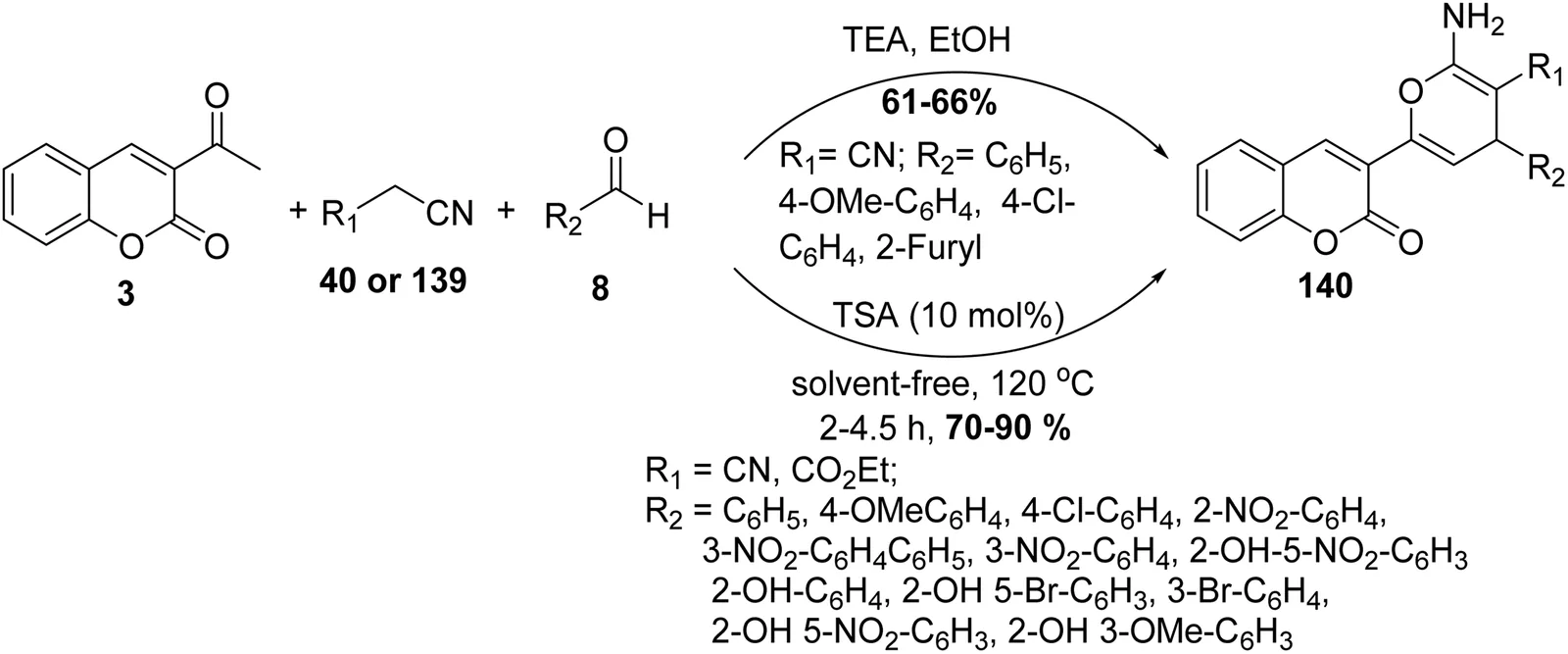
Synthesis of isolated chromen-pyran system 140.

#### 5.2.4. 1,8-Naphthyridine

In 2015, Vadagaonkar *et al.*^485^ reported the synthesis of 4-methyl-2-phenyl-5*H*-chromeno[4,3-*d*]pyrimidin-5-one 142*via* the reaction of 3 with benzamidine hydrochloride 141 using recyclable and greener choline hydroxide (ChOH)^486^ or triethylamine (Scheme 82).

**Scheme 82:**
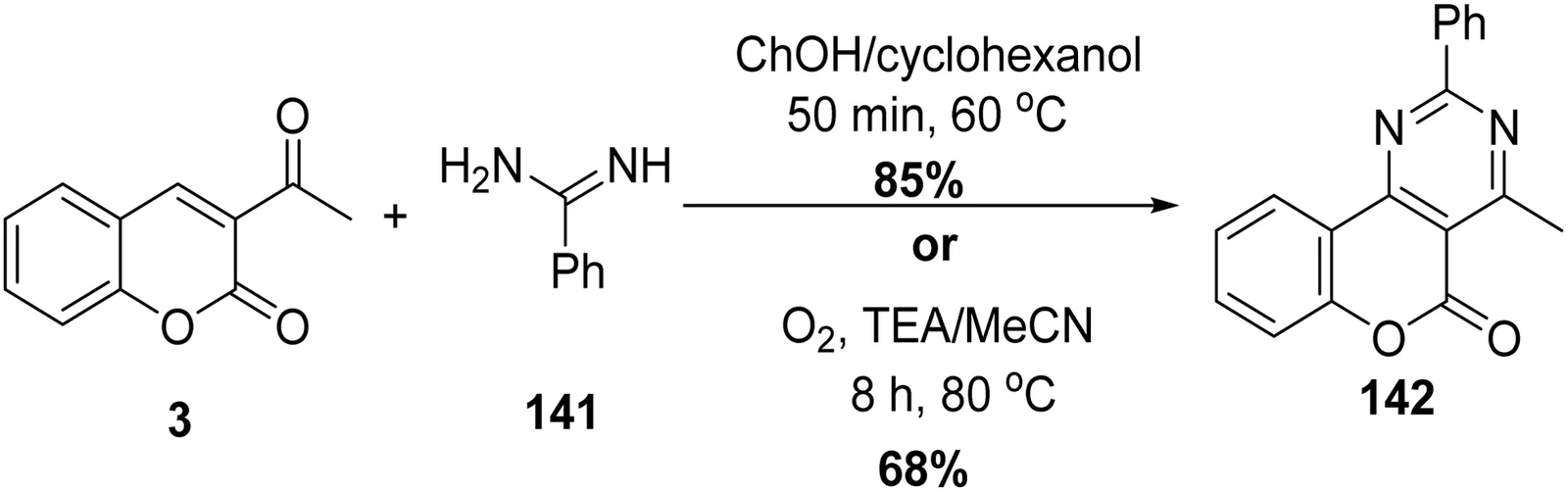
Treatment of 3-acetylcoumarin 3 with benzamidine hydrochloride 141.

#### 5.2.5. Imidazo[1,2-α]pyridine

Heteropolyacid-containing imidazolium-based ionic liquid was applied in multicomponent synthesis of hexahydroimidazo[1,2-α]pyridine derivatives 144*via* the reaction of 3, various aldehydes 8 and ethylenediamine derivatives 143 (Scheme 83).^487^

**Scheme 83:**
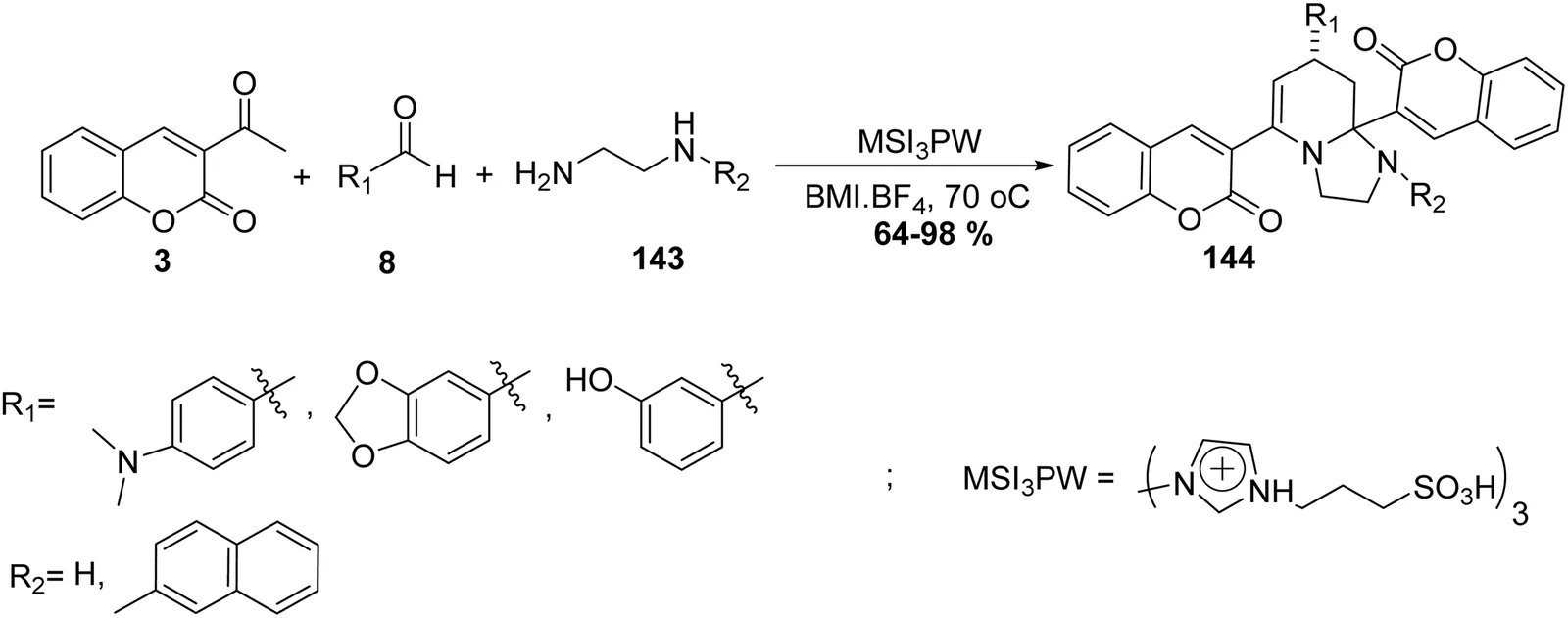
MCRs for the preparation of hexahydroimidazo[1,2-α]pyridine derivatives 144.

#### 5.2.6. Quinoxalines

One-pot synthesis of coumarinyl quinoxalines 146a–j from the reaction of 3-acetylcoumarins 3 and *o*-phenylenediamine derivatives 145 in the presence of tetrabutylammonium tribromide (TBATB) and PEG-600 as a solvent (Scheme 84).^488^

**Scheme 84:**
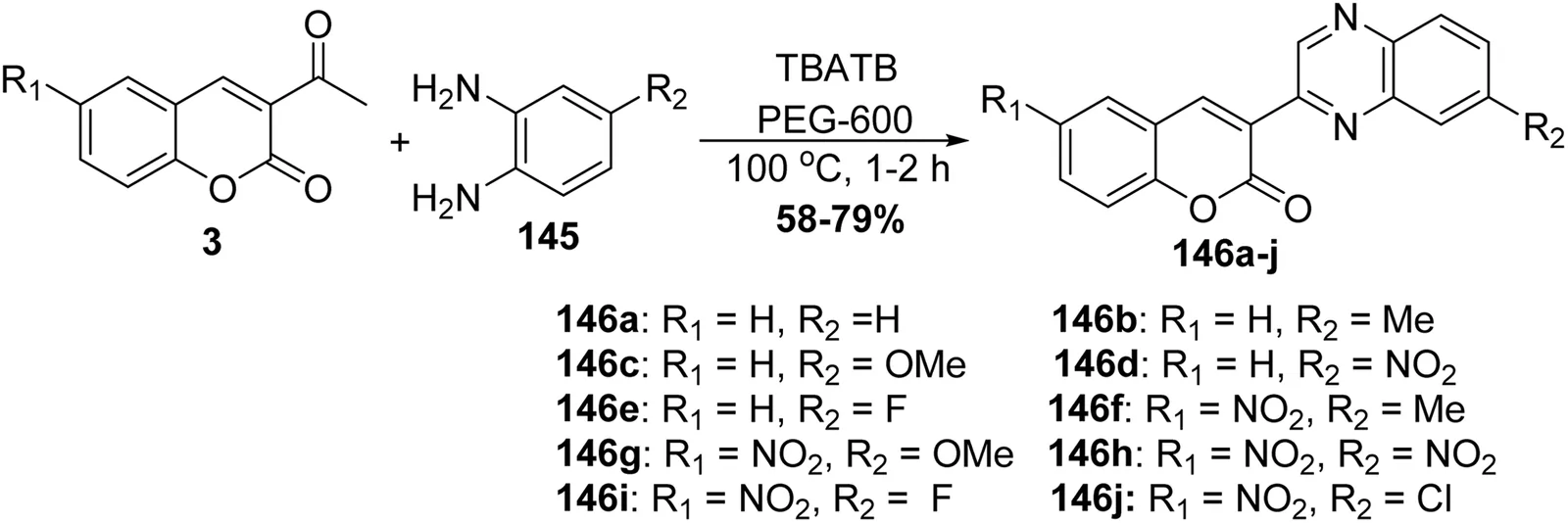
Synthesis of coumarinyl quinoxalines 146a–j.

### 5.3. Seven-membered and higher fused heterocyclic systems

Nanosheet graphene oxide (GO) is found to be an effective recyclable carbon catalyst for the synthesis of multifunctional dibenzo[1,4]diazepine 147 starting from 3, *o*-dimedone 125, and phenylenediamine 145 in an aqueous medium (Scheme 85).^489^

**Scheme 85:**
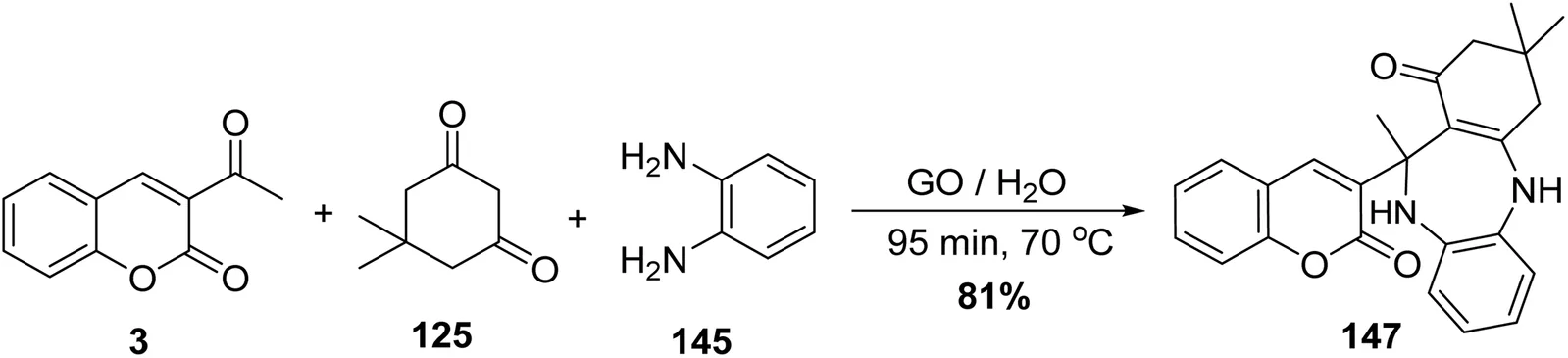
Synthesis of dibenzo[1,4]diazepine 147.

## 6. Biological, analytical and photophysical applications

### 6.1. Biological activities

3-Acetylcoumarins serve as versatile pharmacophoric scaffolds for the synthesis of several bioactive heterocyclic compounds with promising biological profiles, including antibacterial,^247,283,443,490–492^ antiproliferative,^478,493,494^ anti-alzheimer,^404,495–500^ antiprotozoal,^501^ antioxidant,^502,503^ anti-tumor,^187,504,505^ antidiabetic,^506^ and anti-dengue^507^ agents (Fig. 4). Moreover, they paved the way for construction of many coumarin bearing metal complexes promising in many biological applications, especially antioxidant,^326^ antifungal,^343^ antibacterial,^219,343^ and anticancer^508^ studies (Fig. 5).

**Fig. 4:**
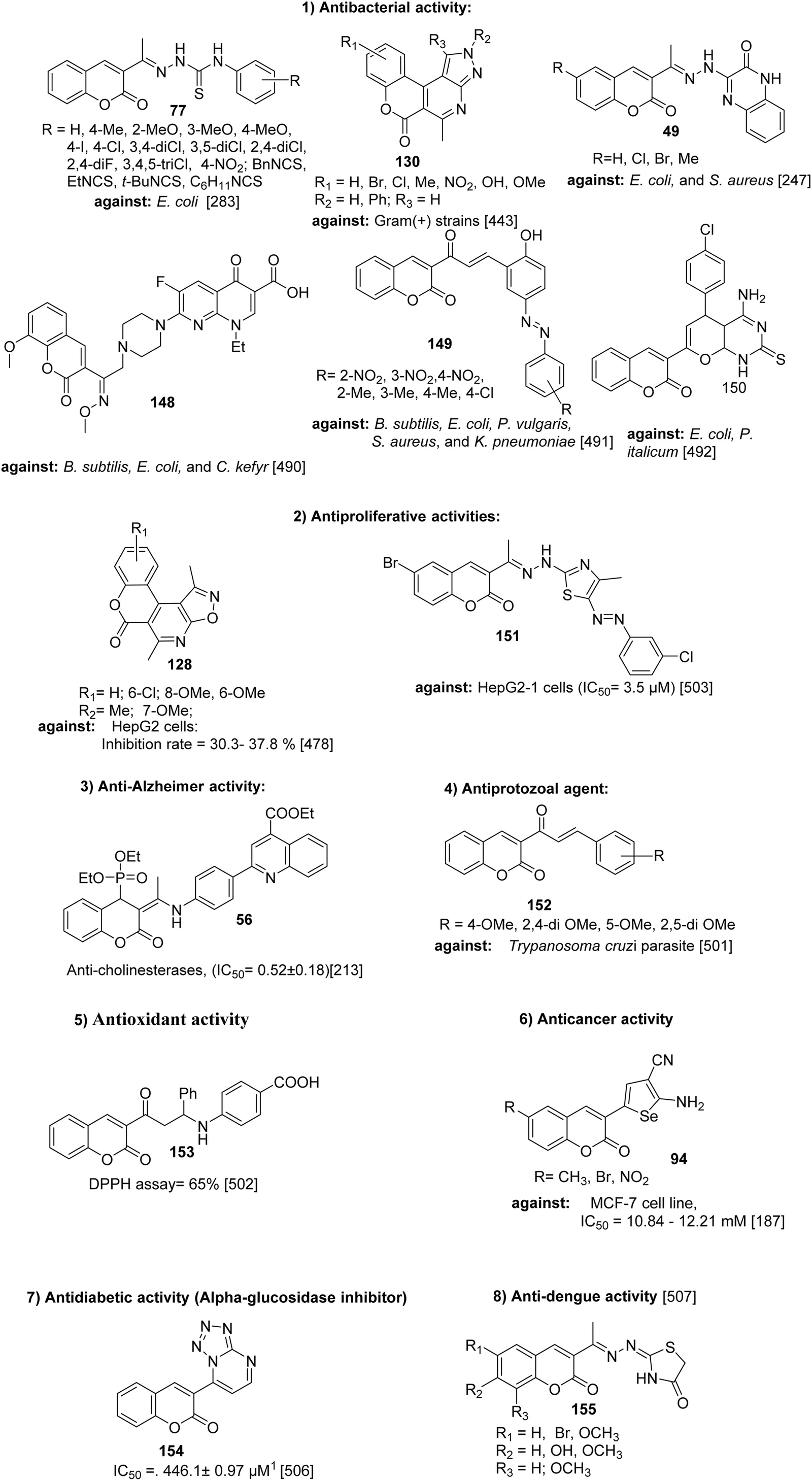
Representative examples of biologically active compounds with potential activity against a wide range of diseases.

**Fig. 5:**
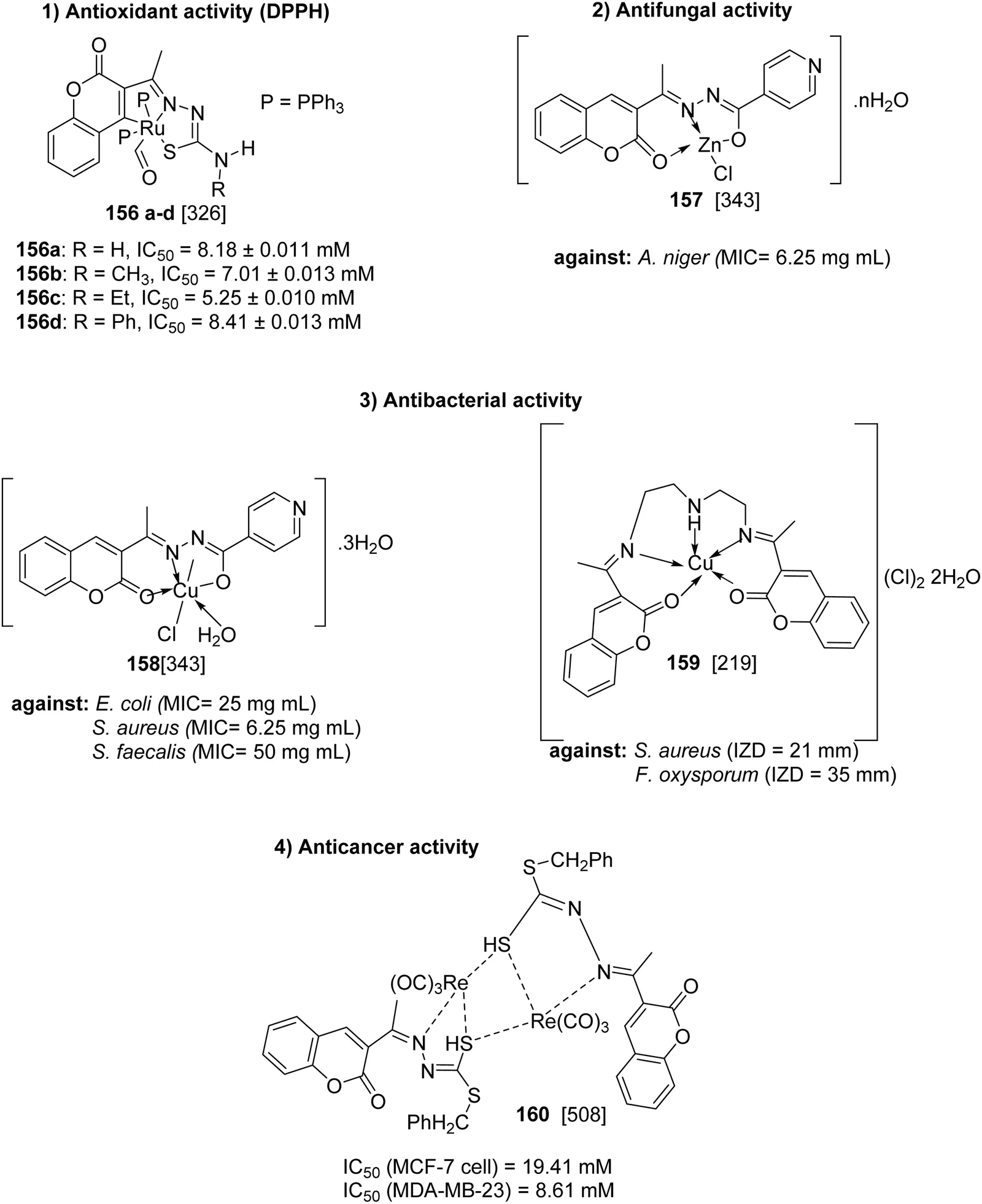
Representative, diverse pharmacological effects of many coumarins bearing metal complexes.

Structure–activity relationship (SAR) considerations of C4-unsubstituted 3-acetylcoumarin derivatives indicate that their biological activities are influenced by the nature, position, and electronic characteristics of substituents introduced into the coumarin scaffold. Modifications at the aromatic ring, particularly the introduction of electron-withdrawing groups such as halogen and nitro substituents, have been associated with enhanced biological activity in several reported derivatives, possibly due to changes in lipophilicity and molecular interactions with biological targets. In contrast, electron-donating groups can modulate activity through alterations in electronic distribution and hydrogen-bonding properties. Furthermore, transformation of the C3-acetyl group into diverse heterocyclic systems, including pyrazole, thiazole, triazole, and other nitrogen- or sulfur-containing derivatives, can improve pharmacological profiles by increasing structural diversity and providing additional interaction sites. Metal coordination of coumarin-based ligands has also been reported to influence biological performance by modifying redox properties and enhancing antimicrobial or anticancer effects. Overall, these SAR considerations highlight the potential of rational modification of the C4-unsubstituted 3-acetylcoumarin framework for optimizing biological activity and developing promising bioactive molecules (Fig. 4 and 5).

### 6.2. Fluorescent chemosensors and analytical applications

3-Acetylcoumarins as privileged building blocks have various applications in the field of fluorescent chemosensors towards anions (Fig. 6A–C),^255,509–513^ metal cations (Fig. 6D and E),^514–516^ and biomolecules (Fig. 6F).^517–521^ Moreover, π-extended coumarin derivatives derived from acetylcoumarins, particularly styryl coumarins, benzocoumarin–styryl hybrids, thiazole–coumarin hybrids, and quadrupolar dye systems, have attracted considerable attention as deep-red/NIR emitters, aggregation-induced emission luminogens, viscosity-sensitive fluorescent molecular rotors, and cyanine-type laser dyes.^522–528^

**Fig. 6:**
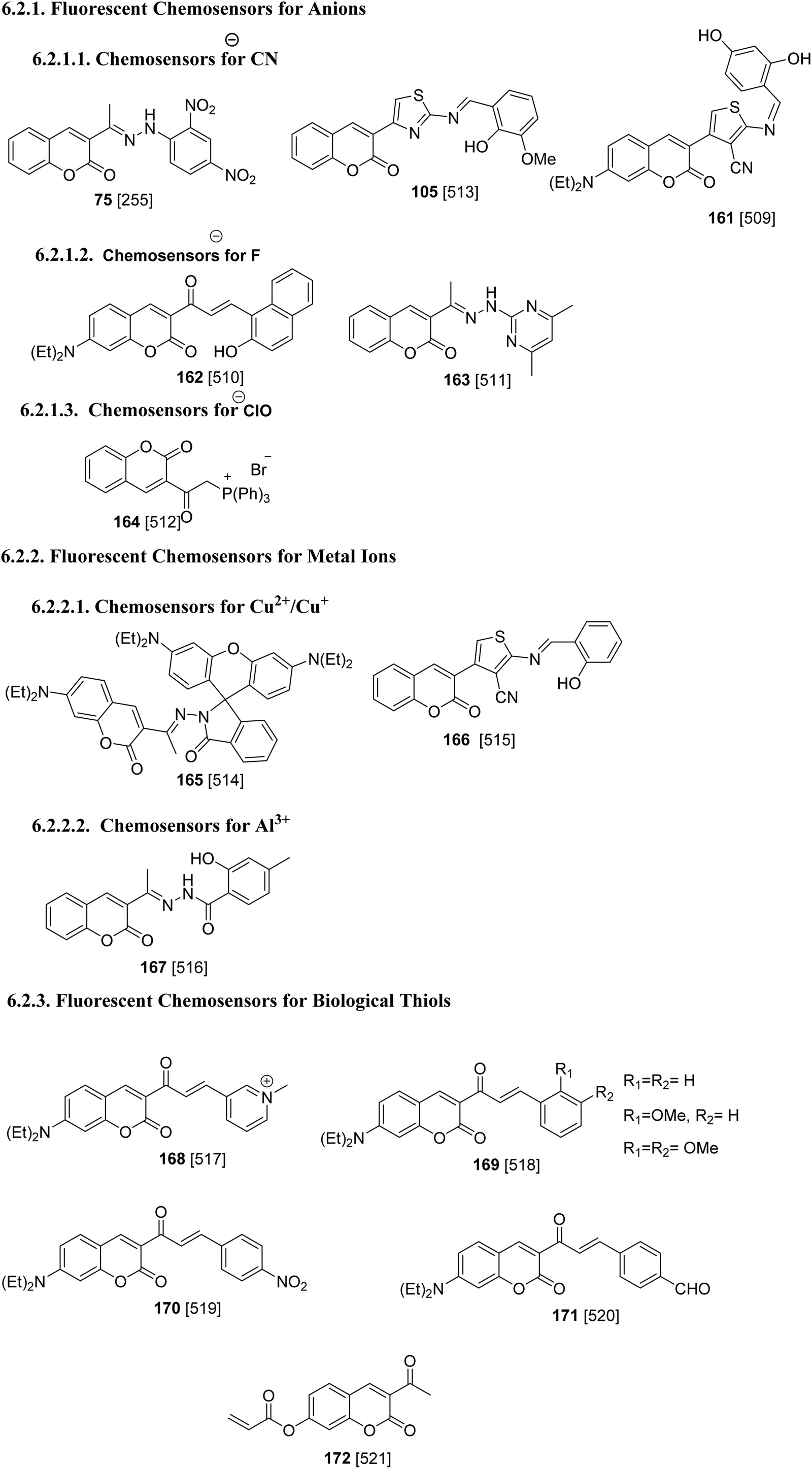
Representative 3-acetylcoumarin-derived fluorescent chemosensors: (A) cyanide; (B) fluoride; (C) hypochlorite; (D) copper ions; (E) aluminium ions; and (F) biological thiols.

The chromophore plays a crucial role in determining the fluorescent properties of 3-acetylcoumarin derivatives. The coumarin core acts as an intrinsic π-conjugated fluorophore, where the extended conjugated system facilitates electronic excitation and emission processes through π–π transitions. Structural modification of this chromophoric system, particularly through π-extension, introduction of electron-donating or electron-withdrawing substituents, and incorporation of heterocyclic moieties, can effectively tune the absorption and emission wavelengths, fluorescence intensity, and sensing performance. In fluorescent chemosensors, the coumarin chromophore generally serves as the signal-generating unit, while attached recognition groups regulate fluorescence responses through mechanisms such as photoinduced electron transfer (PET), intramolecular charge transfer (ICT), or aggregation-induced emission (AIE). Therefore, rational design of the chromophore structure is essential for developing highly sensitive and selective fluorescent probes based on 3-acetylcoumarin scaffolds.

#### 6.2.1. Fluorescent chemosensors for anions

Fluorescent probes derived from 3-acetylcoumarins have been investigated for the recognition of several anionic analytes, particularly cyanide (CN^−^), fluoride (F^−^), and hypochlorite (ClO^−^). The representative structures in Fig. 6A–C illustrate how different recognition fragments can be combined with the coumarin chromophore to generate analyte-responsive optical signals.^255,509–513^

##### 6.2.1.1. Chemosensors for CN^−^

Compounds 75,^255^105,^513^ and 161 (ref. 509) are representative 3-acetylcoumarin-derived chemosensors reported for CN^−^. As illustrated in Fig. 6A, their structures differ in the groups attached to the coumarin fluorophore, including hydrazone- and thiazole-containing motifs. These examples demonstrate the structural diversity of cyanide-responsive coumarin probes and the role of molecular modification in designing selective anion-sensing systems.

##### 6.2.1.2. Chemosensors for F^−^

The fluoride-sensing examples shown in Fig. 6B include compounds 162 (ref. 510) and 163 (ref. 511). Compound 162 incorporates an extended conjugated fragment and a phenolic hydroxyl group, whereas compound 163 contains a nitrogen-rich recognition fragment linked to the coumarin core. These different molecular arrangements highlight the use of substituent and receptor design to modulate fluorescence responses toward F^−^.

##### 6.2.1.3. Chemosensors for ClO^−^

Compound 164 (ref. 512) is presented in Fig. 6C as a representative coumarin-based chemosensor for hypochlorite (ClO^−^). Its structure contains a phosphonium-functionalized acetyl side chain, distinguishing it from the cyanide- and fluoride-responsive derivatives discussed above. This example extends the analytical scope of coumarin probes to reactive oxychlorine species.

#### 6.2.2. Fluorescent chemosensors for metal ions

Coumarin-based fluorescent chemosensors have also been developed for metal-ion recognition, including copper and aluminium species.^514–516^ In these systems, changes to the ligand or recognition fragment connected to the coumarin fluorophore can influence metal binding and the resulting optical response. Panels D and E of Fig. 6 show representative compounds directed toward Cu^2+^/Cu^+^ and Al^3+^.

##### 6.2.2.1. Chemosensors for Cu^2+^/Cu^+^

Compounds 165 (ref. 514) and 166 (ref. 515) illustrate two different structural approaches to copper-ion sensing (Fig. 6D). Compound 165 combines a coumarin-based unit with an additional heterocyclic dye framework, whereas compound 166 bears a thiazole-containing, phenolic recognition fragment. These examples emphasize the use of different donor-atom environments in the design of fluorescence-based probes for Cu^2+^/Cu^+^.

##### 6.2.2.2. Chemosensors for Al^3+^

The 3-acetylcoumarin-derived chemosensor 167 (ref. 516) is an example reported for Al^3+^ detection (Fig. 6E). Its structure features a hydrazone-type linkage together with oxygen-containing functional groups, illustrating a potential coordination environment for interaction with the metal ion. This example highlights the application of coumarin-based recognition units to aluminium sensing.

##### 6.2.3. Fluorescent chemosensors for biological thiols

3-Acetylcoumarin-derived fluorescent probes have also been used for the detection of biologically relevant thiols, as represented by compounds 168–172 (ref. 517–521) in Fig. 6F. The illustrated probes use structurally distinct responsive groups, including conjugated carbonyl-containing substituents and other electrophilic motifs, attached to a coumarin signalling unit. Their diversity demonstrates the adaptability of the 3-acetylcoumarin framework for thiol-responsive molecular design and related analytical applications.

## 7. Conclusion and future perspectives

C4-Unsubstituted 3-acetylcoumarins represent a distinctive and versatile class of coumarin derivatives that combine the intrinsic properties of the coumarin lactone framework with the unique reactivity of the C3-acetyl group and the accessible C4–H position. This review has provided a comprehensive overview of the progress achieved in the synthesis, reactivity, heterocyclic diversity, biological relevance, photophysical properties, and analytical applications of these valuable scaffolds from 2006 to 2026.

The synthetic chemistry of C4-unsubstituted 3-acetylcoumarins has witnessed significant development through diverse approaches, including condensation reactions, C–H functionalization, annulation processes, nucleophilic transformations, and metal-catalyzed methodologies. These strategies have enabled the preparation of a wide range of structurally diverse heterocyclic and fused heterocyclic systems, demonstrating the importance of 3-acetylcoumarins as strategic building blocks in modern organic synthesis. The coexistence of the reactive acetyl functionality and the free C4–H site provides complementary reaction pathways, allowing the construction of complex molecular architectures with potential applications in medicinal chemistry and functional materials.

Beyond their synthetic importance, C4-unsubstituted 3-acetylcoumarin derivatives have demonstrated promising biological activities, including antibacterial, antioxidant, anti-inflammatory, anticancer, enzyme-inhibitory, and neuroprotective effects. Structure–activity relationship studies indicate that biological performance is strongly influenced by substituent effects, electronic properties, and transformation of the acetyl group into diverse heterocyclic frameworks. Moreover, modification of the coumarin chromophore has enabled the development of fluorescent probes and chemosensors, highlighting the potential of these compounds in bioimaging and analytical applications.

Despite the considerable advances achieved, several important research challenges remain. First, the development of greener, more efficient, and scalable synthetic methodologies remains a major goal, particularly catalytic systems that minimize hazardous reagents, reduce energy consumption, and improve atom economy. Although numerous transformations have been reported, mechanistic understanding of several C4-functionalization and annulation reactions remains incomplete, requiring detailed experimental and computational investigations to clarify reaction pathways, regioselectivity, and catalyst–substrate interactions.

Second, biological investigations of C4-unsubstituted 3-acetylcoumarins are still largely based on preliminary activity screening. Future studies should focus on systematic structure–activity relationship (SAR) analyses supported by molecular docking, computational modeling, and target-based biological evaluation to identify the structural features responsible for enhanced activity and selectivity. In addition, the exploration of unexplored biological targets, pharmacokinetic properties, and *in vivo* evaluation remains necessary to translate promising *in vitro* findings into practical therapeutic applications.

Furthermore, although several 3-acetylcoumarin derivatives have shown interesting photophysical properties, the relationship between molecular structure, electronic distribution, chromophore modification, and fluorescence behavior requires deeper investigation. Rational design of donor–acceptor systems, aggregation-responsive materials, and multifunctional fluorescent probes represents an emerging direction for future research. Similarly, the incorporation of these scaffolds into advanced materials, sensing platforms, and optoelectronic systems remains relatively underexplored.

Overall, C4-unsubstituted 3-acetylcoumarins continue to represent an attractive research platform due to their unique structural features, broad chemical reactivity, and multifunctional applications. Future efforts integrating sustainable synthesis, mechanistic studies, computational approaches, advanced biological evaluation, and rational molecular design are expected to further expand the potential of this important class of coumarin derivatives in synthetic chemistry, medicinal chemistry, and materials science.

## Conflicts of interest

There are no conflicts to declare.

## Abbreviations

AcOH: Acetic acid
Ar: Aryl
DABCO: 1,4-Diazabicyclo[2.2.2]octane
DCE: 1,2-Dichloroethane
DEA: Diethylamine
DMF: *N*,*N*-Dimethylformamide
DMSO: Dimethyl sulfoxide
EtOAc: Ethyl acetate
GO: Graphene oxide
i-PrOH: Isopropanol
Me: Methyl
MeOH: Methanol
MCRs: Multicomponent reactions
NMR: Nuclear magnetic resonance
PEG: Polyethylene glycol
Pip: Piperidine
PPTS: Pyridinium *p*-toluenesulfonate
PTC: Phase-transfer catalyst
RT: Room temperature
TBABr_3_: Tetra-*n*-butylammonium tribromide
TBHP: *Tert*-butyl hydroperoxide
THF: Tetrahydrofuran
TUD: Thiourea dioxide
H_2_O_2_: Hydrogen peroxide
NH_4_OAc: Ammonium acetate
BF_3_·OEt_2_: Boron trifluoride diethyl etherate
CQDs: Carbon quantum dots
AcOK: Potassium acetate
Bu: Butyl
DBU: 1,8-Diazabicyclo[5.4.0]undec-7-ene
DCM: Dichloromethane
DMA: Dimethylacetamide
DMF-DMA: *N*,*N*-Dimethylformamide dimethyl acetal
Et: Ethyl
EtOH: Ethanol
h: Hour (s)
IR: Infrared spectroscopy
MeCN: Acetonitrile
min: Minute (s)
MW: Microwave irradiation
ORTEP: Oak Ridge Thermal Ellipsoid Plot
Ph: Phenyl
PivOH: Pivalic acid
*p*-TSA: *p*-Toluenesulfonic acid
R: Alkyl or aryl substituent
TBAB: Tetrabutylammonium bromide
TBATB: Tetrabutylammonium tribromide
TEA: Triethylamine
TMSOTf: Trimethylsilyl trifluoromethanesulfonate
°C: Degree Celsius
K_2_S_2_O_8_: Potassium persulfate
PhI(OAc)_2_: Iodobenzene diacetate
HClO_4_–SiO_2_: Silica-supported perchloric acid
PS-TBD: Polymer-supported triazabicyclodecene

## Data Availability

No new data were created or analysed in this review article. All information discussed and summarized in this manuscript was obtained from previously published studies and is available in the cited references.
